# Antithrombotic coating with sheltered positive charges prevents contact activation by controlling factor XII–biointerface binding

**DOI:** 10.1038/s41563-024-02046-0

**Published:** 2024-11-12

**Authors:** Haifeng Ji, Kai Yu, Srinivas Abbina, Lin Xu, Tao Xu, Shengjun Cheng, Sreeparna Vappala, S. M. Amin Arefi, Md Mohosin Rana, Irina Chafeeva, Matthew Drayton, Kevin Gonzalez, Yun Liu, Dana Grecov, Edward M. Conway, Weifeng Zhao, Changsheng Zhao, Jayachandran N. Kizhakkedathu

**Affiliations:** 1 Centre for Blood Research & Life Science Institute, University of British Columbia, Life Sciences Centre, Vancouver, British Columbia Canada; 2 Department of Pathology and Laboratory Medicine, University of British Columbia, Vancouver, British Columbia Canada; 3 College of Polymer Science and Engineering, State Key Laboratory of Polymer Materials Engineering, Sichuan University, Chengdu, People’s Republic of China; 4 School of Biomedical Engineering, University of British Columbia, Vancouver, British Columbia Canada; 5 Department of Mechanical Engineering, University of British Columbia, Vancouver, British Columbia Canada; 6 Chengdu First People’s Hospital, Chengdu, People’s Republic of China; 7 Department of Medicine, University of British Columbia, Vancouver, British Columbia Canada

**Keywords:** Biomaterials - proteins, Biomedical materials

## Abstract

Antithrombotic surfaces that prevent coagulation activation without interfering with haemostasis are required for blood-contacting devices. Such materials would restrain device-induced thrombogenesis and decrease the need for anticoagulant use, thereby reducing unwanted bleeding. Here, by optimizing the interactions with coagulation factor XII rather than preventing its surface adsorption, we develop a substrate-independent antithrombotic polymeric coating with sheltered positive charges. The antithrombic properties of the coating were demonstrated in vitro with human blood and in vivo using a carotid artery–jugular vein shunt model in rabbits. The coating exhibits a strong interaction with factor XII, but results in a low reciprocal activation of the contact pathway that triggers clot formation. These findings contradict the prevailing strategy of designing antithrombotic materials through protein-repelling surfaces. Overall, the polymeric coating we describe can benefit most blood-contacting devices and is a useful engineering guideline for designing surfaces with improved antithrombotic properties.

## Abstract (web summary)

A polymeric coating with sheltered positive charges establishes strong interactions with coagulation factor XII without inducing surface-induced coagulation activation, being a promising antithrombotic coating for biomedical devices.

## Main

Blood is a complex tissue consisting of a multitude of proteins and cells, with a wide range of regulators to achieve a balanced state between anticoagulation and coagulation, thereby ensuring physiological flow in vivo. When synthetic surfaces are exposed to blood, the contact pathway of coagulation is activated, initially triggered via the cleavage of the zymogen factor XII (FXII) to its active form FXIIa^1–3^. Indeed, antithrombotic surfaces that can totally prevent the contact activation of coagulation are currently not available^4^. Thus, anticoagulants are often administered in conjunction with artificial devices or implants to prevent device/implant-associated thrombogenesis^5^, but this inherently increases the bleeding risk^6,7^. A few different approaches have been investigated to prevent thrombus generation on surfaces with varying degrees of success, but they also have challenges^4,8–11^.

Antithrombic surfaces can reduce thrombotic risk by preventing thrombogenesis without affecting haemostasis and is thus the holy grail of any blood-contacting surface design. Since the surface-induced conformational change in FXII is recognized as the first event that initiates coagulation activation^2,3,12^, inhibiting FXII–surface interaction (for example, using antifouling surfaces) is intuitively a reasonable approach towards antithrombotic surfaces^13–16^. However, this strategy relies on the unproven premise that antifouling surfaces do not activate coagulation proteins that are desorbed from the surface. Although many surface designs based on protein repelling have claimed to possess the antithrombotic functionality^13–16^, the close link between coagulation and anticoagulation in blood complicates these assertions. Furthermore, the dislodged clot or clot initiation at distant sites from antifouling surfaces can lead to more serious consequences^17^. Besides, recent insights into the nature of loosely bound proteins have revealed that antifouling surfaces are unable to prevent the adsorption of many proteins in vivo^18–20^ and thus have the potential to trigger coagulation.

Despite these uncertainties, the predominant and possibly the sole design approach for antithrombotic surfaces is based on inhibiting protein–surface interactions. Therefore, conducting a thorough analysis on their limitations and proposing a more promising strategy could serve as a crucial turning point in the design of true antithrombotic surfaces. Building on the current knowledge of blood-contacting surfaces, this study aims to (1) analyse why antifouling or protein/cell-repelling surfaces are not fully protected against thrombus formation; (2) to propose a new strategy for the design of a universal substrate-independent antithrombotic surface coating for blood-contacting applications; and (3) to demonstrate how the new surface coating can prevent surface-initiated thrombogenesis in vitro in human plasma, whole blood and in a relevant animal model.

## Concept, design and synthesis of new antithrombotic coating

**Fig. 1:**
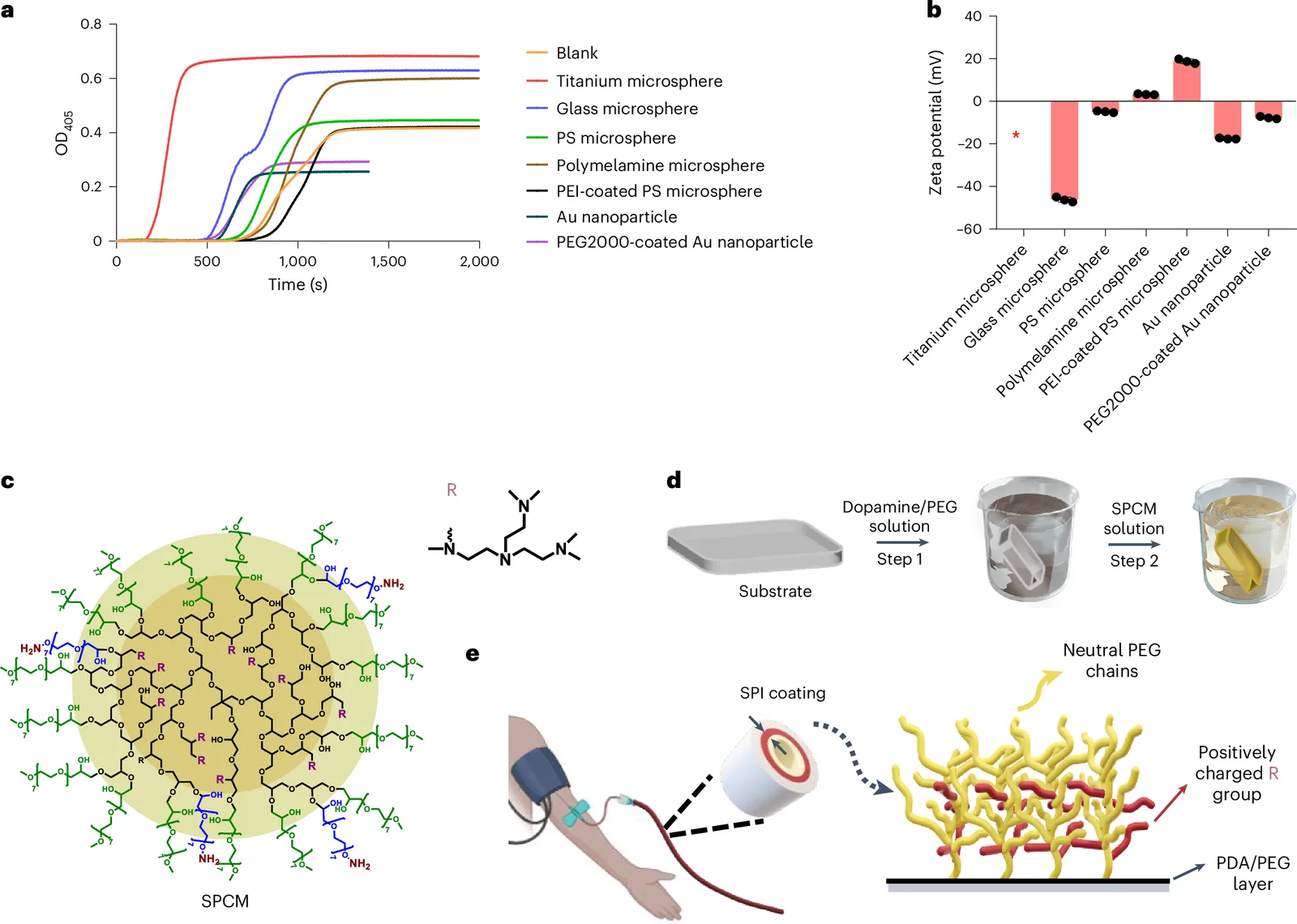
Concept and synthesis of SPI coating. **a**, Clotting time of recalcified plasma after incubation with different surfaces. OD_405_, OD at 405 nm. At least *n* = 3 biologically independent measurements are used. PS, polystyrene. **b**, Zeta potential of different surfaces. The red asterisk indicates that the measurements cannot be performed for this particle. **c**, Chemical structure of SPCM. The molecule has an HPG core decorated with R-based cationic binding groups, and a brush layer of methoxy PEG (mPEG) to protect the R groups from non-specific interactions. The primary amine groups at the end of the PEG reacts with the quinone groups of the PDA/PEG coating to generate the SPI coating. **d**, Schematic of the generation of an SPI coating on different substrates. **e**, Cartoon illustrating the macrostructure and microstructure of an SPI-coated catheter used for the development of a blood-contacting device. Source data

We began our studies by examining the effects of coagulation activation in the plasma of various surfaces with distinct surface properties. These included negatively charged surfaces (glass and titanium), positively charged surfaces (polymelamine and polyethylenimine (PEI)-coated polystyrene (PS)), hydrophobic surfaces (PS), metallic surfaces (titanium and gold) and neutral hydrophilic surfaces (polyethylene glycol (PEG)-modified gold) (Fig. 1a,b). Most of the tested synthetic surfaces were in their nanoparticulate form, including those with antifouling properties (PEG-modified gold), and they shortened the plasma-clotting time compared with plasma alone. The one exception to this finding, however, was positively charged surfaces, which had minimal effects on plasma clotting. This raised the possibility of a new approach to develop antithrombotic surfaces using cationic surfaces. However, conventional cationic surfaces are toxic in blood; such interfaces bind proteins, activate blood cells, initiate unwanted immune reactions and generate cell toxicity, possibly due to their strong electrostatic binding to proteins and cells^21,22^. Thus, we reasoned that developing a surface that does not initiate coagulation activation akin to cationic surfaces and simultaneously improve haemocompatibility could potentially lead to a new antithrombotic surface design. In our previous research, we have developed a library of biocompatible sheltered positively charged macromolecules (SPCMs) (for example, universal heparin reversal agents (UHRAs) and macromolecular polyanion inhibitors)^21,23–25^. Compared with naked positively charged macromolecules, these SPCMs maintained strong interactions with target biomolecules while demonstrating favourable haemocompatibility.

Inspired by this, we designed a universal substrate-independent coating based on surface-conjugated SPCMs. We refer to this new surface as a selective protein interacting (SPI) coating to achieve control over the protein interaction and selective protein binding with sheltered positive charges. To develop the SPI coating, we initially synthesized an SPCM, which consists of a hyperbranched polyglycerol (HPG) core decorated with ~11 methylated tris(2-aminoethylamine) (R) groups and is protected with a shell layer of methoxy PEG (mPEG350) chains. The molecule is functionalized with approximately four primary amines at the end of PEG chains for surface conjugation (Fig. 1c). The synthesis scheme, characterization and properties of modified SPCM are shown in Supplementary Figs. 1–4.

The next step in the generation of the SPI coating is the development of a substrate-independent coating. A polydopamine (PDA)-based coating consisting of PDA/PEG covalently conjugated with SPCM was used, as this allows for its application to diverse biomedical devices or surfaces without pretreatment (Fig. 1d)^26^. The successful conjugation of SPCM on the PDA/PEG coating was verified by attenuated-total-reflectance Fourier transform infrared spectroscopy (Supplementary Fig. 5), X-ray photoelectron spectroscopy (Supplementary Fig. 6), quartz crystal microbalance (Supplementary Fig. 7) and ellipsometry (Supplementary Fig. 8). The stability of the SPI coating was confirmed by the absence of thickness change and spectral features by exposing the coating to harsh conditions (Supplementary Figs. 5, 7 and 8). The surface morphology of the SPI coating revealed a grainy uniform surface feature (Supplementary Figs. 9 and 10). The water contact angle of the SPI coating (~10°) confirms the highly hydrophilic nature of the coating (Supplementary Fig. 11a), and the surface zeta potential measurements (Supplementary Fig. 11b) showed that the SPI coating has a slight negative charge (−7 mV), unlike the SPCM or PDA/PEG coating. A more detailed surface characterization of the SPI coating is provided in the Supplementary Results. The proposed macrostructure of the SPI coating is shown in Fig. 1e.

## SPI coating resists surface-induced thrombogenesis

**Fig. 2:**
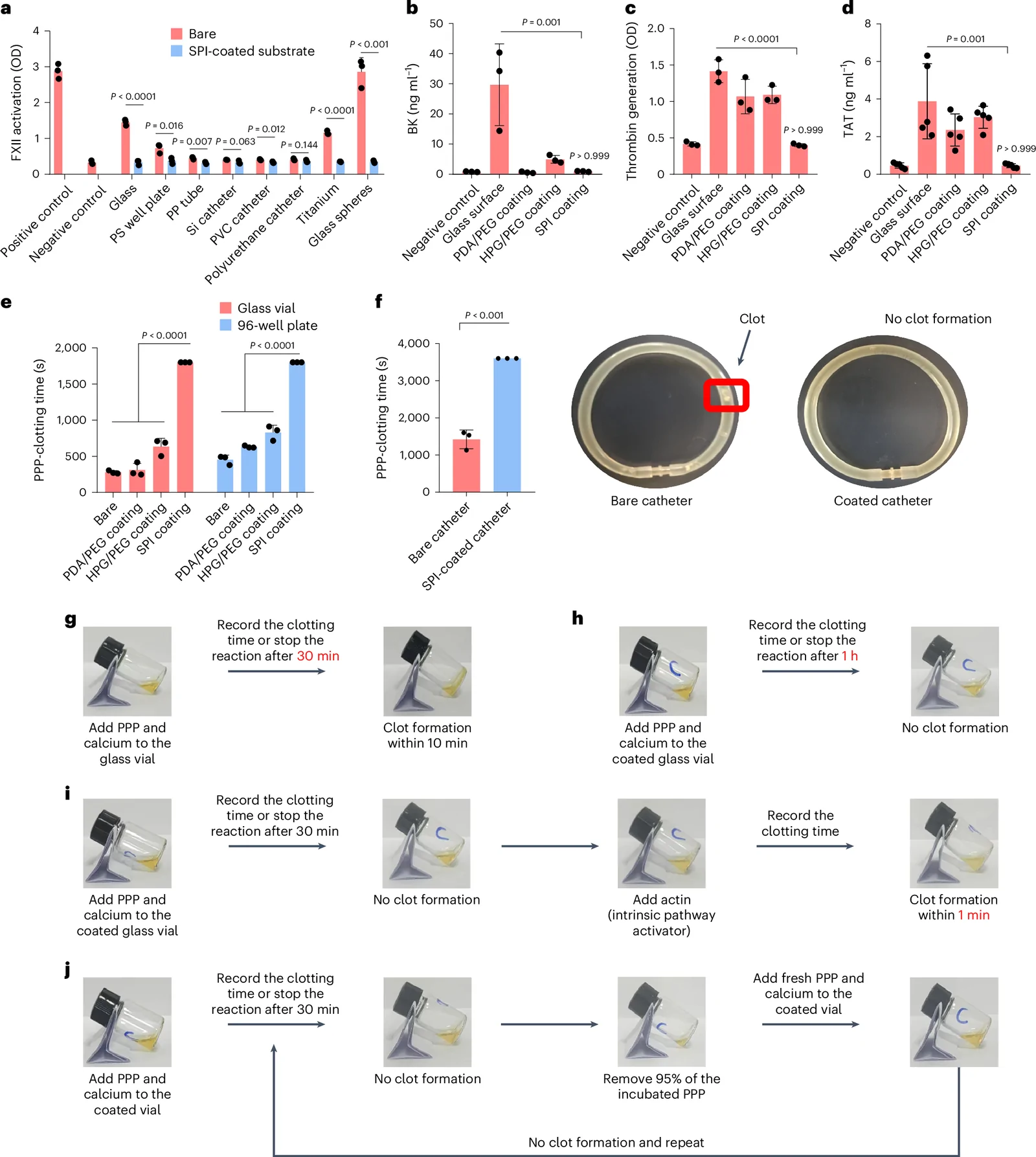
SPI coating prevents surface-induced contact activation and thrombogenesis. **a**, Cleavage efficiency of the chromogenic FXIIa substrate S-2302 in human plasma incubated with different substrates with or without an SPI coating. **b**, BK generation in the plasma incubated with glass vials with and without coatings. Different coatings were compared. **c**, Cleavage efficiency of the chromogenic thrombin substrate S-2238 in recalcified plasma incubated with different coatings. **d**, TAT complex generation in recalcified plasma incubated with glass vials with and without coatings. **e**, Clotting time of the recalcified plasma incubated with glass or well plate with or without an SPI coating. **f**, Closed loop with silicone tubing with or without an SPI coating for the evaluation of the coating’s antithrombotic function under shear condition. Left: the clotting time of the recalcified plasma incubated with a silicone catheter with or without an SPI coating. Right: photograph of the clot on the catheter at the end of the experiment. **g**–**j**, Photographs of the recalcified plasma incubated with a glass vial with or without an SPI coating after a predetermined procedure. Statistical analysis: for **a**, unpaired, two-tailed Student’s *t*-test was applied for intragroup comparison for each substrate with or without an SPI coating. Two-way ANOVA with Bonferroni’s post hoc analysis was applied for a comparison between the SPI-coated substrate and the negative control (PPP). For **b**–**d**, uncalcified PPP was used as the negative control. Multiple comparisons were performed using one-way ANOVA. If significance was determined, post hoc multiple comparisons analysis was conducted with a Tukey’s test. For **e**, one-way ANOVA was applied for intragroup comparison for each substrate with different coatings. If significance was determined, post hoc multiple comparisons analysis was conducted with a Tukey’s test. For **f**, unpaired, one-tailed Student’s *t*-test was applied. If not specified otherwise, *n* = 3 biologically independent analyses were performed. All values are expressed as the mean ± s.d. If not specified otherwise, individual *P* values represent comparisons with the negative control. Exact *P* values are provided in the corresponding figures. Statistical significance was defined as *P* < 0.05. Source data

Since most surfaces activate coagulation via the contact pathway, we investigated the effect of SPI coating on the contact pathway activation of blood coagulation in human plasma. The SPI coating was prepared on biomaterials with diverse surface chemistry that are commonly used for biomedical devices and implants. The cleavage efficiency of the chromogenic substrate S-2302 for FXIIa and kallikrein was used as a measure of contact activation after incubating the SPI-coated surfaces and control surfaces in plasma. As shown in Fig. 2a, contact activation by the SPI-coating-incubated plasma was significantly less compared with the uncoated surface, and showed no significant difference compared with the control plasma, whereas other hydrophilic control coatings such as PDA/PEG and HPG/PEG coatings maintain the surface-induced contact activation (Supplementary Fig. 12).

We next investigated whether the SPI coating induced the activation of other proteins in the contact pathway. Here we used glass as a positive control, which is a potent contact pathway activator. Compared with other coatings, the SPI coating significantly inhibited bradykinin (BK) generation (Fig. 2b) in plasma. The SPI coating also significantly inhibited thrombin generation as measured with the chromogenic substrate S-2238 (Fig. 2c), and the formation of thrombin–antithrombin (TAT) complexes (Fig. 2d)^27^. The SPI coating, furthermore, significantly prolonged the plasma-clotting time compared with other coated substrates (Fig. 2), as well as on coated polymeric catheters under shear conditions (60 s^−1^) (Fig. 2f). These data provide the initial evidence of the antithrombotic properties of the SPI coating.

Additional clotting studies were performed in recalcified plasma using coated glass vials (Fig. 2g–j and Supplementary Video 1). An unmodified glass surface induced clot formation within 10 min (Fig. 2g), whereas the SPI coating prolonged the clotting time to more than 1 h (Fig. 2h). Furthermore, the SPI coating restrained clot formation on the surface without significantly interfering with the haemostatic function of plasma. This was shown by the following: after an initial 30 min incubation of plasma with the SPI coating, coagulation was triggered by introducing actin. Results show that clotting was not inhibited or delayed in plasma incubated with the SPI coating (Fig. 2i). Further, compared with traditional heparin coatings and antifouling surfaces, the SPI coating demonstrated superior antithrombotic functionality (Supplementary Figs. 13 and 14). Moreover, the antithrombotic functionality of the SPI coating remained robust even after prolonged storage, and its effectiveness was not significantly diminished even after the adsorption of other plasma proteins (Fig. 2j and Supplementary Figs. 15 and 16).

## SPI coating is haemocompatible

**Fig. 3:**
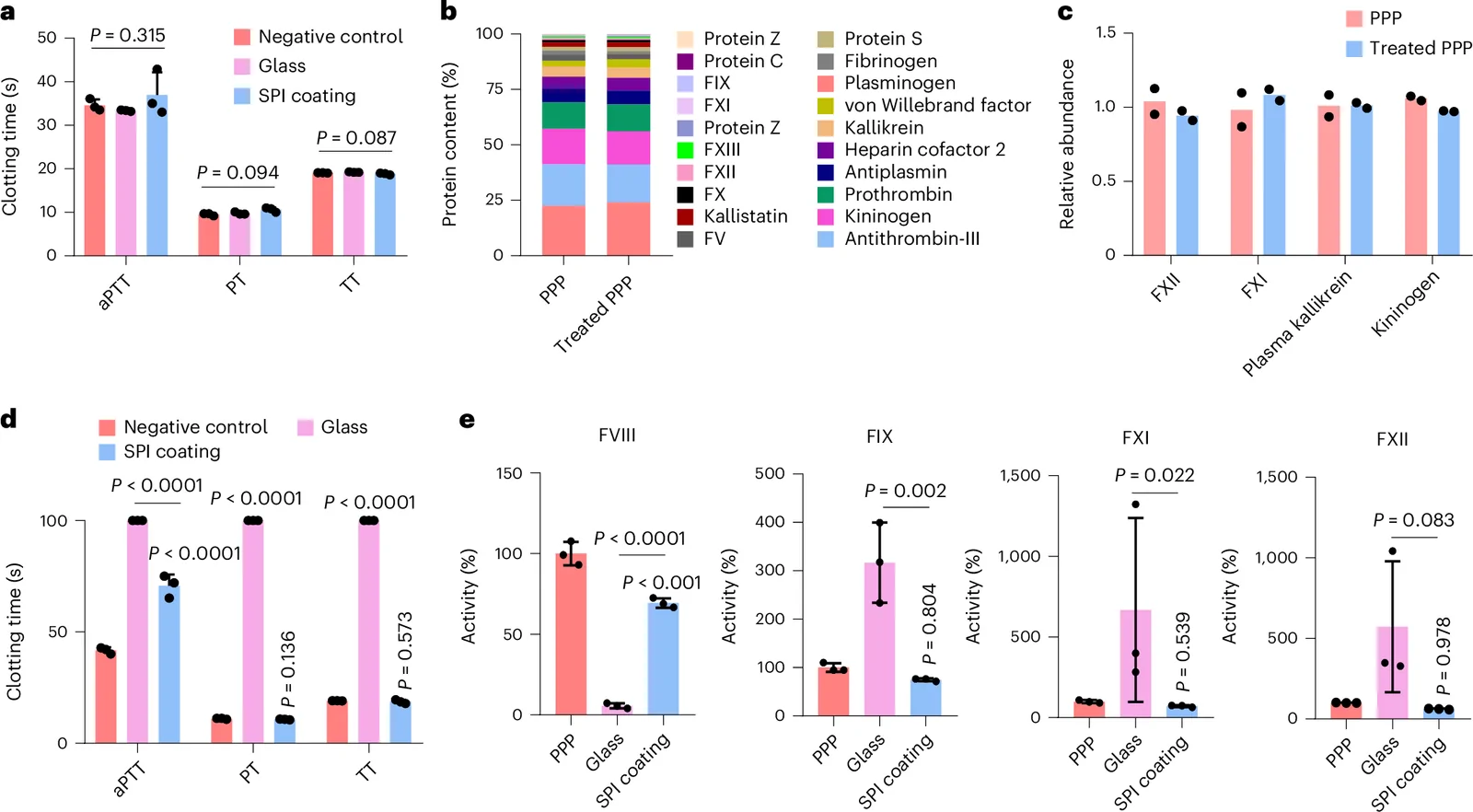
SPI coating prevents thrombin generation without interfering haemostasis. **a**, TT, aPTT and PT were measured using human PPP after incubation with bare and SPI-coated glass vials (PPP was used as the negative control; *n* = 3 biologically independent samples; all values are expressed as the mean ± s.d.). One-way ANOVA was used for intragroup comparison and no significant difference was found. **b**, Abundance of coagulation proteins as the percentage of total proteins in the plasma pre- and post-incubation with SPI coating (*n* = 2 biologically independent samples; all values are expressed as the mean). **c**, Relative abundance of contact-system-related proteins in the plasma pre- and post-incubation with an SPI coating (*n* = 2 biologically independent samples). **d**, TT, aPTT and PT in recalcified PPP after incubation with bare and SPI-coated glass vial (PPP was used as the negative control; *n* = 3 biologically independent samples; all values are expressed as the mean ± s.d.). One-way ANOVA was used for intragroup comparison for each substrate with different coatings. If significance was determined, post hoc multiple comparisons analysis was conducted with a Tukey’s test. **e**, Activity of FVIII, FIX, FXI and FXII belonging to the intrinsic pathway of the plasma after incubation with bare and SPI-coated glass vial (*n* = 3 biologically independent samples; all values are expressed as the mean ± s.d.). One-way ANOVA was used for intragroup comparison for each substrate with different coatings. If significance was determined, post hoc multiple comparisons analysis was conducted with a Tukey’s test. If not specified otherwise, individual *P* values represent comparisons with the PPP control. Exact *P* values are provided in the corresponding figures; statistical significance was defined as *P* < 0.05. Source data

Traditional anticoagulant materials/therapeutics can be complicated by disruptions in normal haemostasis, with a remaining risk of bleeding^12^. The SPI coating is not expected to interfere with normal coagulation; rather, it is designed to decrease surface-induced contact activation. As shown in Fig. 3a and Supplementary Fig. 17, prothrombin time (PT), activation partial thromboplastin time (aPTT), thrombin time (TT) and fibrinogen concentrations in plasma were not changed from normal after 30 min incubation with the SPI coating. The SPI coating also did not activate coagulation factor VII (FVII), thereby preventing the downstream activation of coagulation and fibrinolytic systems (Supplementary Fig. 18)^28^. A proteomic study was also used to confirm that the concentrations of different coagulation proteins in plasma incubated with the SPI coating did not appreciably change from normal (Fig. 3b and Supplementary Table 1), especially for those proteins involved in the contact activation (Fig. 3c).

Activated FXII can trigger the downstream activation of coagulation. To explore the potential impact of SPI coating on the coagulation system in the absence of an anticoagulant, we incubated recalcified plasma with glass and SPI-coated glass and stopped the coagulation reaction at designed intervals by re-adding sodium citrate. The PT, aPTT and TT values were markedly prolonged when plasma was incubated with a bare glass surface (Fig. 3d) due to the depletion of coagulation factors, particularly fibrinogen. In comparison, the SPI coating did not affect the PT or TT value, whereas the aPTT value was slightly prolonged. The prolonged aPTT observed in SPI-coating-incubated plasma may be attributed to the inevitable contact activation of air exposure in our experimental conditions. The activated coagulation factors that are promptly neutralized by other inhibitory proteins, such as C1 esterase inhibitor (C1INH) and antithrombin, resulting in the depletion of intrinsic coagulation factors, which, in turn, causes the slight prolongation of aPTT^29^. Further analysis revealed that the activities of factor IX (FIX), factor XI (FXI) and FXII were significantly boosted, whereas that of factor VIII (FVIII) was depleted from the plasma on glass incubation. However, the activities of FIX, FXI and FXII were only slightly attenuated in SPI-coating-incubated plasma (Fig. 3e). The SPI coating also resists the adhesion and activation of platelets and neutrophils (Supplementary Figs. 19–25) as well as shows good cell compatibility (Supplementary Figs. 26–28) and low complement activation (Supplementary Figs. 29 and 30). Details are analysed and given in the Supplementary Results.

## SPI coating is antithrombotic in an arteriovenous shunt model

**Fig. 4:**
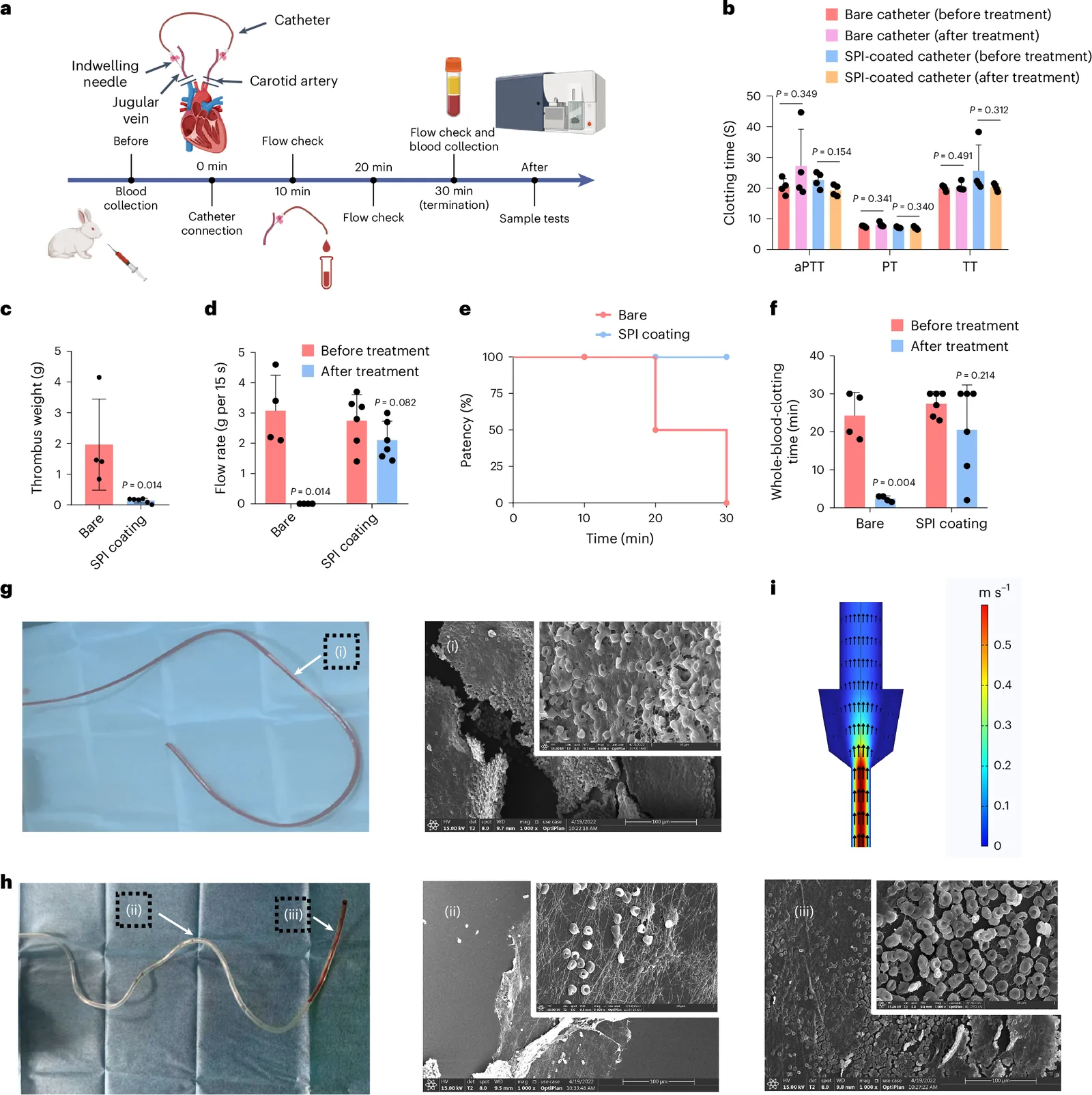
SPI coating is antithrombotic in the AV shunt model. **a**, Schematic showing the experimental setup to evaluate the antithrombotic characteristics of the SPI coating in an AV shunt model in rabbits without anticoagulant administration. **b**, Comparison of aPTT, PT and TT for the bare or SPI-coated catheter before and after treatment in rabbits. Paired one-way ANOVA was applied for intragroup comparison and no significant difference was found. **c**, Comparison of thrombus weight for the bare (uncoated) or SPI-coated catheter after treatment. Unpaired, two-tailed Student’s *t*-test was applied. **d**, Comparison of blood flow rate through the uncoated or SPI-coated catheter before and after treatment. Paired, two-tailed Student’s *t*-test was applied for intragroup comparison. **e**, Comparison of the flow patency of the blood through the uncoated or SPI-coated catheter at different time intervals. **f**, Clotting time of the whole blood collected from the uncoated or SPI-coated catheter before and after treatment. Paired, two-tailed Student’s *t*-test was applied for intragroup comparison. **g**,**h**, Digital (left) and scanning electron microscopy (right) photographs for the blood clotting adhered on the uncoated catheter (**g**) and SPI-coated catheter (**h**). **i**, Computational fluid dynamics model for the catheter-indwelling needle connection. *n* = 4 biologically independent samples for the uncoated catheter group and *n* = 6 biologically independent samples for the SPI-coated catheter group. All values are expressed as the mean ± s.d. Exact *P* values are provided in the corresponding figures; statistical significance was defined as *P* < 0.05. Source data

We investigated the antithrombotic function of the SPI coating using an arteriovenous (AV) shunt model in rabbits to evaluate intracatheter thrombosis without anticoagulant administration^16,30^. The experimental design is shown in Fig. 4a. Blood flow is from the carotid artery to the jugular vein through an indwelling needle connected to a polyvinyl chloride (PVC) catheter, which is either modified with an SPI coating or used without modification. The experiments were performed for 30 min. To assess the haematological parameters of the animals during the procedure, blood samples were collected before and after the experiment. The coagulation function of the animals remained normal throughout the experiment as measured by the aPTT, PT and TT (Fig. 4b). There were also no significant changes in the body temperature (Supplementary Table 2), whole blood cell count (Supplementary Fig. 30) and other parameters (Supplementary Table 3) during the procedure.

Thrombus formation within the catheter was measured (Fig. 4c,d). After 30 min, the uncoated catheter induced severe intracatheter thrombosis (Fig. 4c) and blood flow through the catheter was almost stopped (Fig. 4d) and the catheter was blocked (Fig. 4e). However, the SPI-coated catheter significantly reduced surface-induced thrombosis (Fig. 4c). No significant difference was observed in the blood flow rate through the SPI-coated catheter at 30 min compared with the flow rate at the start of the experiment (Fig. 4d). The data showed that the SPI-coated catheter maintained patency (Fig. 4e). Moreover, the clotting time of the blood collected from the bare catheter after the procedure was significantly shortened compared with blood collected at the start of the experiment. There was no significant difference in clotting time between the blood collected from the SPI-coated catheter before and after the procedure (Fig. 4f). The data indicated that coagulation was activated on the uncoated catheter surface and the SPI-coated catheter resisted activation. Moreover, thrombus adherence to the SPI-coated catheters was significantly reduced (Fig. 4h) compared with the unmodified catheters (Fig. 4g), as shown in the digital and scanning electron microscopy images. However, we observed some clot deposition on part of the SPI-coated catheter connected to the artery through the narrow needle (Fig. 4h(iii)). We attribute this to the dramatic change in local haemodynamics (Fig. 4i and Supplementary Fig. 31) when blood flows through the narrow needle and exits into the catheter, as simulated by computational fluid dynamics. The alternation in haemodynamics results in platelet activation, leading to local coagulation activation^31,32^. Since the SPI coating does not have inherent anticoagulant properties, it is unable to prevent clotting activation arising from non-self-induced factors.

## SPI coating strongly interacts with FXII without activation

**Fig. 5:**
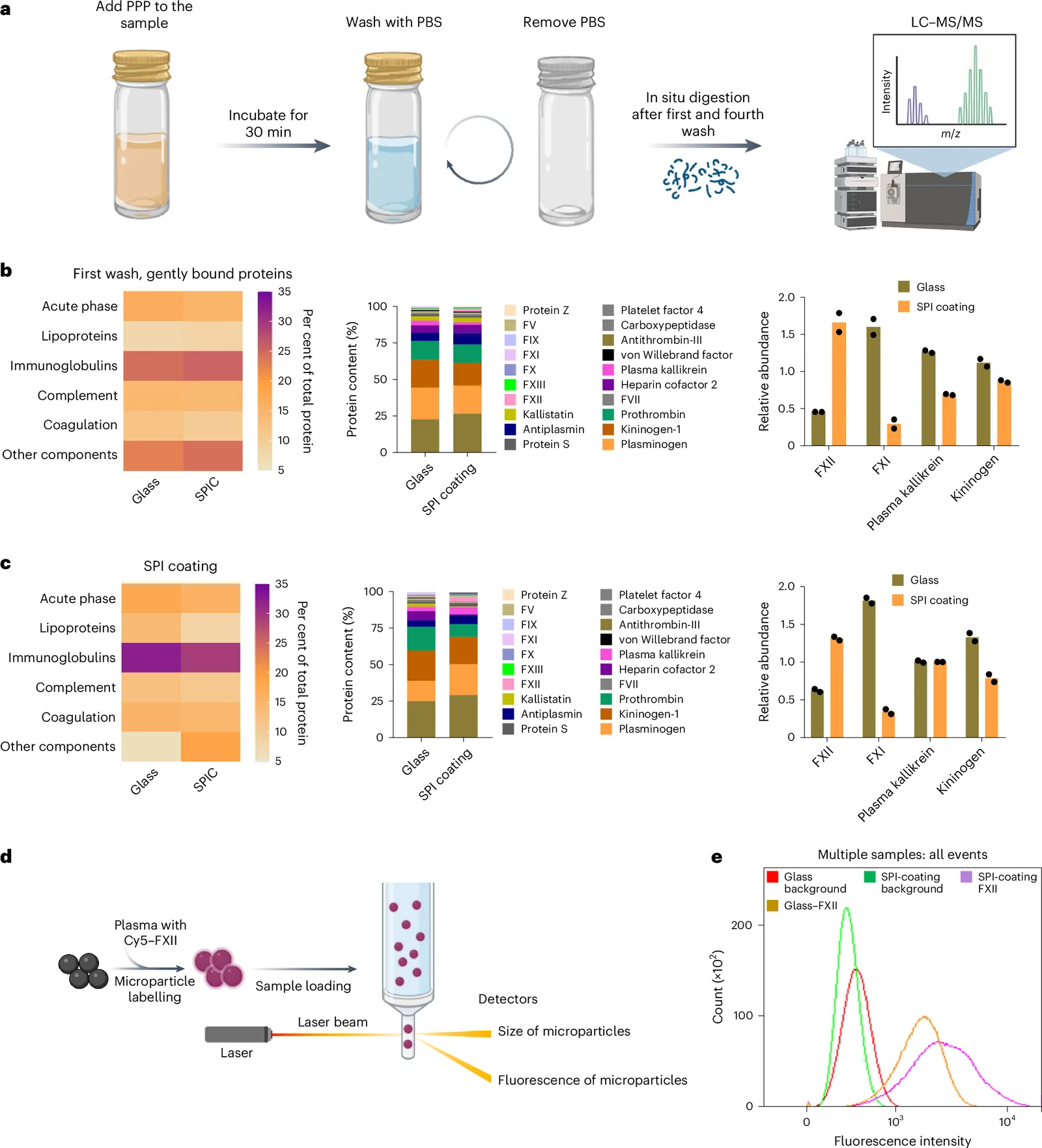
FXII strongly binds to an SPI-coated surface. **a**, Schematic for the detection of loosely or tightly bound proteins on bare or SPI-coated glass surface by proteomic analysis. **b**, Composition of loosely bound proteins on bare or SPI-coated glass surface by proteomic analysis. Left: abundance of each functional protein group as the percentage of total proteins. Middle: abundance of coagulation proteins as the percentage of total proteins. Right: comparison of the relative abundance of contact-system-related proteins loosely adsorbed on bare or SPI-modified glass surface. *n* = 2 biologically independent samples; all the values are expressed as the mean. **c**, Composition of tightly bound proteins on bare or SPI-modified glass surface by proteomic analysis. Left: abundance of each functional protein group as the percentage of total proteins. Middle: abundance of coagulation proteins as the percentage of total proteins. Right: a comparison of the relative abundance of contact-system-related proteins tightly adsorbed on bare or SPI-coated glass surface. *n* = 2 biologically independent samples; all values are expressed as the mean. **d**, Schematic of the in situ FXII adsorption behaviour on the surface. Glass microspheres with or without an SPI coating were incubated with FXII-deficient plasma externally added with Cy5-labelled FXII. The fluorescence intensity of the Cy5-labelled FXII adsorbed onto the surface was in situ detected by the flow cytometry method. **e**, Fluorescence intensity of the surfaces before and after Cy5-labelled FXII adsorption. Since the entire process does not involve surface separation and washing, the adsorbed proteins are retained as much as possible. The content of FXII in both hard protein corona and soft protein corona on the surface is reflected by the fluorescence intensity. The higher the fluorescence intensity, the more Cy5-labelled FXII is incorporated onto the material surface (500,000 events were recorded for each sample in FXII-deficient plasma). *n* = 3 biologically independent samples. The fluorescence intensity on the surface was measured after 30 min incubation. LC–MS/MS, liquid chromatography tandem mass spectrometry. Source data

Although we showed that the SPI coating significantly reduced contact activation and thrombogenesis, it is not clear as to how the SPI coating generates this activity. We analysed the adsorbed protein corona on the SPI coating from plasma to understand the role of biointerface-adsorbed proteins and their antithrombotic functions. The adsorbed proteins can be loosely or tightly bound to the surface, and their composition and surface organization can potentially affect the functionality of the material surface (Supplementary Figs. 32–34)^33,34^. Thus, we designed different washing steps to investigate the composition of loosely and tightly bound proteins by proteomics analysis, respectively, on glass and SPI-coated surfaces (Fig. 5a)^19^.

After gentle washing, the loosely bound proteins were largely retained on both surfaces. Although the amounts of proteins were similar, the bare glass and SPI-coated surfaces showed different compositions (Fig. 5b, Supplementary Fig. 35a and Supplementary Table 4). The glass surface adsorbed more coagulation proteins and lower amounts of immunoglobulins than the SPI coating. We then focused on proteins in the contact activation system, and found that the SPI-coated surface adsorbed more FXII than on the glass surface. The amounts of FXI, prekallikrein and kininogen on the SPI-coated surface were lower than those on the glass surface. Thorough washings distinguished the compositions of loosely bound and tightly bound proteins^33^. The glass surface adsorbed more immunoglobulins and coagulation proteins than the SPI coating. However, the SPI-coated surface had more adsorbed FXII than the glass surface (Fig. 5c, Supplementary Fig. 35b and Supplementary Table 5). The data illustrated that the SPI-coated surface interacted strongly with FXII in plasma compared with the glass surface. We also conducted the in situ detection of FXII adsorption onto glass microspheres, both with and without the SPI coating, using the flow-cytometry-based technique^35^. The results aligned with those of the proteomics analysis, showing that the SPI coating adsorbed more FXII (Fig. 5d and Supplementary Figs. 36 and 37). Detailed results and explanation are provided in the Supplementary Results.

Taken together, the results of protein corona analyses and flow-cytometry-based assay allowed us to conclude that the SPI coating exhibits strong interactions with FXII. These results also point to the fact that the strong interaction of FXII with the surface may not be a sufficient condition for the initiation of surface-induced coagulation activation.

## Mechanism of modulation of the contact activation by SPI coating

Our data demonstrated that a strong interaction between a surface and FXII may not be a prerequisite condition for its activation and initiation of the contact pathway of coagulation. We further analysed the effect of different surfaces, including the SPI coating (low thrombogenic), HPG/PEG coating (neutral antifouling surface, moderately thrombogenic) and glass (highly thrombogenic), on the contact activation system to probe the mechanism of inhibition of surface-induced thrombogenesis by the SPI coating. Since contact activation in plasma is regulated by various proteins, an in vitro simulation of FXII–PK reciprocal activation was measured in a simplified system (detailed results and explanation are available in the Supplementary Results)^36^.

**Fig. 6:**
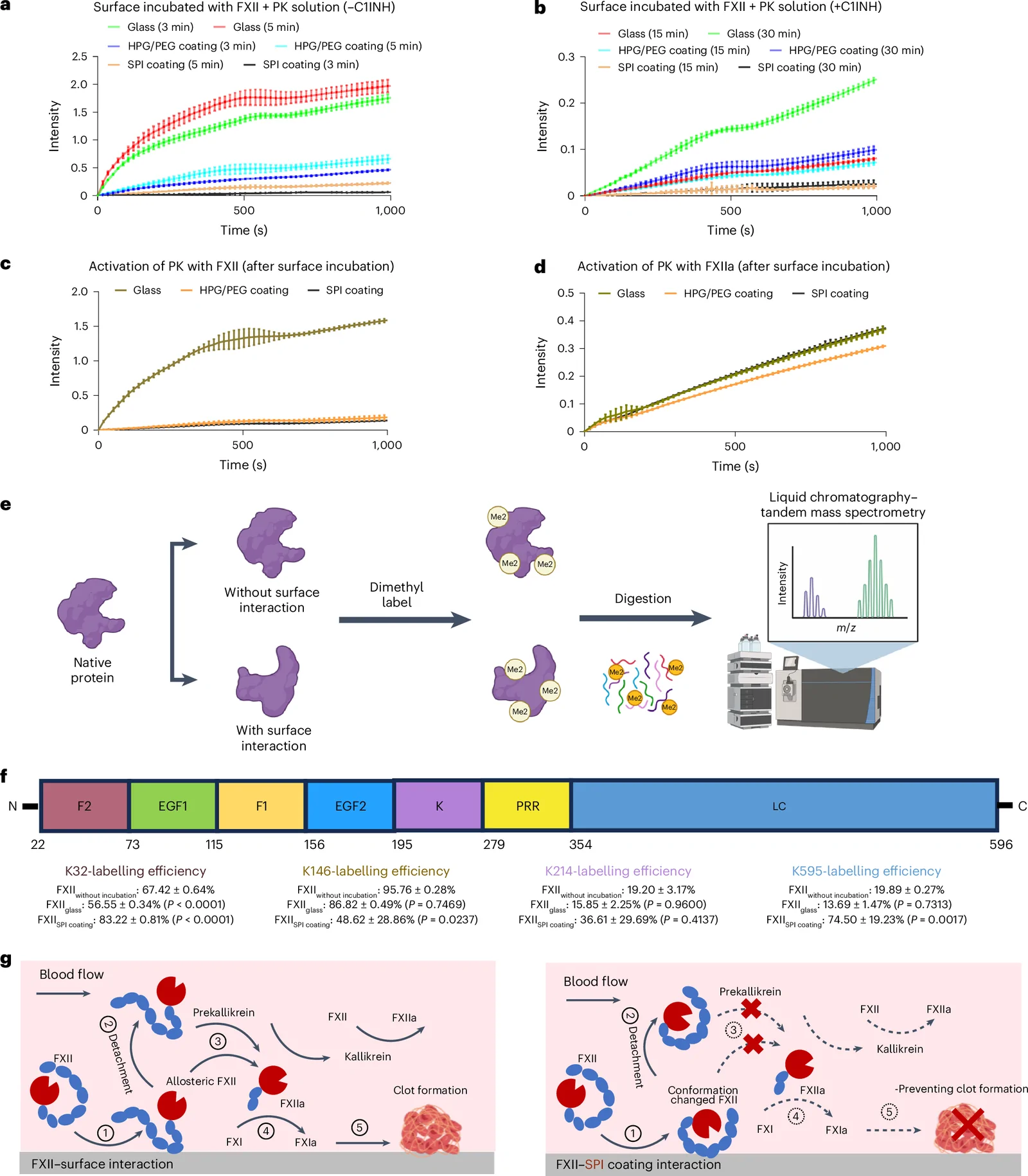
Mechanism of contact activation modulation by SPI coating. **a**, Cleavage efficiency of S-2302 in the absence of inhibitor (C1INH) by an in vitro simulated contact initiation system (400 nM FXII and 400 nM PK in a PBS buffer) after incubation with different surfaces at different time intervals. The slope can represent the catalytic activity of the incubated contact initiation system. *n* = 3 biologically independent samples; all values are expressed as the mean ± s.d. **b**, Cleavage efficiency of S-2302 in the presence of inhibitor (1 μM C1INH) by an in vitro simulated contact initiation system (400 nM FXII and 400 nM PK in a PBS buffer) after incubation with different surfaces at different time intervals. *n* = 3 biologically independent samples; all values are expressed as the mean ± s.d. **c**, FXII solution (final concentration, 400 nM) was first incubated with different surfaces for 10 min and then the solution was co-incubated with PK (final concentration, 400 nM) in a new PP tube, and the cleavage efficiency of S-2302 for the mixture was recorded. *n* = 3 biologically independent samples; all values are expressed as the mean ± s.d. **d**, FXIIa solution (final concentration, 50 nM) was first incubated with different surfaces and then the solution was co-incubated with PK (final concentration, 400 nM) in a new PP tube, and the cleavage efficiency of S-2302 for the mixture was recorded. *n* = 3 biologically independent samples; all values are expressed as the mean ± s.d. **e**, Schematic showing the evaluation of material-induced FXII conformational change using the proteomic approach. **f**, Labelling efficiency for selected dimethyl-labelled lysines. *n* = 3 biologically independent samples; all values are expressed as the mean ± s.d. Multiple comparisons were performed using one-way ANOVA. If significance was attained, post hoc multiple comparisons analysis was conducted with a Tukey’s test. Exact *P* values are provided in the corresponding figures; statistical significance was defined as *P* < 0.05. Individual *P* values represent comparisons with the native FXII. **g**, Schematic of the mechanistic understanding on how an SPI coating prevents surface-induced thrombosis without interfering with haemostasis. Source data

After incubating the FXII–PK mixture with different surfaces, cleavage of the substrate S-2302 was quantified. The catalytic activity of glass and HPG/PEG coating was at least 30 and 4 times higher than that of the SPI coating, respectively (Fig. 6a). The extent of S-2302 cleavage by the different surfaces corresponded with their propensity towards thrombogenesis. This was further confirmed by sodium dodecyl sulfate–polyacrylamide gel electrophoresis (SDS–PAGE) analyses (Supplementary Fig. 38). In the presence of C1INH^37^, the catalytic activity of the FXII–PK mixture incubated with the SPI coating was negligible compared with that observed with the glass surface and HPG/PEG coating (Fig. 6b). We observed that these material surfaces did not induce the hydrolysis of FXII and PK in the solution (Supplementary Fig. 39a,b). However, the FXII incubated with the glass surface could significantly enhance the reciprocal activation with PK even after the surface was removed from the solution (Fig. 6c and Supplementary Fig. 41). Unlike the glass surface, such activity was not seen in the case of the SPI coating. Additionally, after incubation with different surfaces, no significant activity changes were observed for FXIIa (Fig. 6d), PK (Supplementary Fig. 39c) and KK (Supplementary Fig. 40), indicating that the inhibition of contact activation by the SPI coating was not due to any changes to FXIIa, PK and KK.

Since the dissociation of FXII after its interaction with different surfaces was detected using flow cytometry (Supplementary Fig. 42), we investigated whether the surface-dissociated FXII undergoes conformational changes. A modified targeted proteomics approach and dimethyl-labelling techniques were applied (Fig. 6e)^38,39^. Our studies revealed that the labelling efficiency of lysine for the surface-dissociated FXII was significantly different from its native state (Fig. 6f), both for the bare glass and SPI-coated surface. However, these structural changes for SPI coating, unlike other surfaces, did not result in the enhanced activity of FXII^36,40^ (refer to the detailed explanation in the Supplementary Results). Our hypothesis is summarized in Fig. 6g. For conventional synthetic surfaces, FXII can undergo certain conformational changes that can initiate and enhance contact activation, whether the protein is adsorbed or desorbed from the surface. However, in the case of SPI coating, even though a surface-induced conformational change in FXII is unavoidable, contact activation initiation does not occur, which results in the lack of generation of FXIIa, marked reduction in material-surface-induced thrombogenesis and retention of normal haemostasis.

## Outlook

Here we have demonstrated a new concept in the design of a universal antithrombotic surface by altering FXII binding to the surface with favourable haemocompatibility, and that the polymeric coating reduces surface-induced thrombogenesis without interfering with normal haemostasis. The SPI coating, with a sheltered positively charged surface, can prevent the reciprocal activation of FXII–PK (the ‘Supplementary extended outlook’ section in the Supplementary Information provides a detailed discussion). Deviating from the conventional wisdom for generating blood-contacting surfaces that utilize protein-resistant or antifouling surfaces, we propose a new direction—by generating a protein-binding SPI coating that avidly interacts with FXII but renders it inactive in terms of initiating the contact pathway of coagulation. We have demonstrated this concept in preventing surface-induced thrombogenesis in both in vitro studies in human blood and using rabbit extracorporeal shunt model without anticoagulant administration.

Besides generating a universal antithrombotic surface for blood-contacting devices, this work also significantly enhances our understanding on the interaction of proteins in the contact pathway with surfaces. This, in turn, provides new design strategies for the next generation of blood-contacting surfaces with improved haemocompatibility. Specifically, we have identified the following. (1) FXII–PK reciprocal activation already occurs under physiological conditions, and antifouling surfaces may increase this activation. Maintaining the activity of the FXII–PK complex affected by surface interaction under physiological conditions is more important in generating an antithrombotic surface. (2) On the basis of our proteomic analyses, coagulation proteins, especially FXII, are adsorbed onto almost all surfaces. However, surface–FXII interaction/binding may not be a prerequisite for the activation of FXII, and all such surface interactions may not increase FXII–PK reciprocal activation. (3) Surface-induced coagulation activation may not be limited to the biointerface. The proteins that are released from the surface can activate coagulation away from the surface. Thus, current strategies used in the development of antithrombotic surfaces that are based on antifouling surfaces (the most commonly used approach) need critical reconsideration. The ‘Supplementary extended outlook’ section in the Supplementary Information provides an additional discussion.

Although our concept of controlling FXII on the surface led to the generation of a new antithrombotic material, there are still many questions that need to be addressed. The SPI coating attracts more blood proteins than conventional antifouling surfaces. Thus, a key question to be answered is whether single-layer or multilayer adsorption of FXII dominates the SPI coating–FXII interaction. In addition, it is important to understand whether the adsorption of other plasma proteins has a significant impact on the SPI coating–FXII interaction. Is there any correlation between the characteristics of the SPI coating and the specific binding domain of FXII? How does it relate to the desorption behaviour of conformation-altered FXII from the SPI coating? How do different FXII–surface binding sites affect its conformational change^36^, which conformations of FXII are prone to induce coagulation activation or inhibition and how stable is the conformation-altered FXII in plasma? These fundamental questions need to be answered in the future and may provide a much deeper understanding on the mechanism(s) of action of the SPI coating and its function as an antithrombotic surface. The current study provides a foundation for such future studies.

## Methods

### Ethics statement for blood collection

Our research complies with all the relevant ethical regulations. Human blood was collected at the Centre for Blood Research, University of British Columbia (the protocol for blood collection was approved by the University of British Columbia’s clinical ethics board: University of British Columbia Ethics approval nos. H10-01896, H20-00084 and H18-02553) and at the West China Hospital, Sichuan University (GB/T 16886.4-2003/ISO 10993-4:2002, General Administration of Quality Supervision, Inspection and Quarantine of the People’s Republic of China, Standardization Administration of the People’s Republic of China). Informed written consent was obtained before the blood draw from donors in accordance with the Declaration of Helsinki. Fresh human blood was collected from healthy unmedicated donors using vacutainer blood collection tubes. The use of anticoagulants is described in detail in each experimental section. Blood was centrifuged at 156*g* for 15 min at room temperature to obtain platelet-rich plasma, at 2,000*g* for 15 min to obtain platelet-poor plasma (PPP) or treated by EasySep Direct Human Neutrophil Isolation Kit to obtain neutrophil-rich plasma.

### Ethics statement for animal experiments

Our research complies with all relevant ethical regulations. All procedures involving the use of animals in this study were prospectively reviewed and approved by the Institutional Animal Care and Use Committee. This study was conducted in accordance with the National Institutes of Health Guide for the care and use of laboratory animals (NIH publication no. 8023, revised 1978). This experiment conformed to the legal requirement in China and was approved by the ethical committee (no. K2016027) of the West China Hospital, Sichuan University. During the experimental period, the animals had free access to water and food.

### Synthesis of modified SPCM

Supplementary Information provides details on the precursors used in the synthesis of SPCM. UHRA-10 (1 g) and 200 mg epoxy-PEG_400_-azide (~5 mol eq. of UHRA-10) were dried under a vacuum at 60 °C overnight. Then, UHRA-10 was dissolved in 30 ml anhydrous tetrahydrofuran at 70 °C under argon, and 200 mg of NaH was added to the solution. The reaction was kept under stirring at 60 °C for 10 h under argon. After that, 200 mg epoxy-PEG_400_-azide was dissolved in 20 ml anhydrous tetrahydrofuran, and the solution was added to the reaction solution under argon protection over a period of 10 min under stirring. Then, the reaction was maintained at 75 °C for 24 h. The resulting solution was concentrated by rotary evaporation and was dissolved in 10 ml deionized (DI) water. Then, the product was purified by dialysis (molecular weight cut-off, 2,000) for 24 h, during which the water was changed three times. The solution was dried using a lyophilizer to give the azide-modified UHRA-10. Fourier transform infrared spectra had a peak of 2,100 cm^−1^ (peak of the azido group; Supplementary Fig. 4a). ^1^H nuclear magnetic resonance analysis is given in Supplementary Fig. 3b.

After that, 1 g azide-modified UHRA-10 was dried under a vacuum at 60 °C overnight and then dissolved in 80 ml tetrahydrofuran. Then, 400 mg triphenylphosphine was added to the reaction, and after that, 40 ml DI water was added to the solution under stirring for 48 h at 50 °C. After the reaction, the solution was concentrated by rotary evaporation and dissolved in 100 ml DI water, and washed with 100 ml ethyl acetate twice to remove triphenylphosphine and oxidized triphenylphosphine. The resulting water solution was purified by dialysis (molecular weight cut-off, 1,000) for 24 h, during which the water was changed three times. The solution was dried using a lyophilizer to give the final primary-amine-surface-functionalized SPCM. The Fourier transform infrared spectra peak at 2,100 cm^−1^ disappeared.

### Synthesis of SPI coating, HPG/PEG coating and PEI coating

The glass substrate was immersed in a dopamine/PEG coating solution (2 mg ml^–1^ dopamine, 10 mg ml^–1^ MeO-PEG-OH (5,000 Da) and 5.3 mg sodium periodate per ml in sodium acetate buffer solution (pH 5)) for 20 min (for glass vials) or 10 min (for glass microspheres). Afterwards, the substrate was washed with DI water to obtain the PDA/PEG coating^41^.

For the SPI coating, the PDA/PEG-coated glass was immersed in primary-amine-surface-functionalized SPCM solution (0.5 mg ml^–1^ in phosphate-buffered saline (PBS), pH 8.5) under room temperature for 24 h. For the HPG/PEG coating and PEI coating, the PDA/PEG-coated substrate was immersed in amine–HPG/PEG or PEI (*M*_n_, ~10,000 Da) solution (0.5 mg ml^–1^ in PBS, pH 8.5) under room temperature for 24 h. The HPG/PEG coating and PEI coating were used as control groups for blood analysis. Supplementary Information provides a detailed characterization of the coating.

### FXII activation

Citrate-anticoagulated PPP (3.8% sodium citrate with anticoagulant/blood ratio of 1:9) and S-2302 (Chromogenix) were used in this study^1^. To demonstrate the substrate independence of the coating procedure, the coating was applied to glass vials, PS well plate, polypropylene (PP) centrifuge tube, silicone tubing, PVC tubing, polyurethane tubing, titanium cups and glass macroparticle. A mixture of 80 μl of PPP, 20 μl of normal saline and 20 μl of 4 mM substrate S-2302 was incubated with different coatings for 30 min at 37 °C under shaking. Then, 100 μl of the mixture was transferred to a 96-well plate immediately and the optical density (OD) was measured at 405 nm in a plate reader. PPP (80 μl) added with 20 μl normal saline without surface incubation was used as the negative control.

To determine the effect of coatings on FXII activation, different coatings including PDA/PEG, HPG/PEG and SPI were applied on glass vials. The glass vials were incubated with a mixture of 100 μl of PPP, 50 μl of normal saline and 100 μl of 4 mM substrate S-2302 for 30 min at 37 °C under shaking. Then, 100 μl of the mixture was transferred to a 96-well plate immediately and the OD was measured at 405 nm in a plate reader. PPP added with normal saline without surface incubation was used as the negative control. PPP was obtained from at least three individuals, and each experiment was repeated independently at least three times.

### BK activation

Activation level of the kallikrein/kinin system by the coating was measured via ELISA (Human BK Enzyme Immunoassay Kit, Abcam)^10^. Different coatings including PDA/PEG, HPG/PEG and SPI were applied to glass vials. Then, 200 μl PPP was introduced and incubated for 30 min at 37 °C under shaking. Finally, the measurements were conducted according to the respective instruction manuals. PPP without incubation was used as the control. PPP was obtained from at least three individuals, and each experiment was repeated independently at least three times.

### Thrombin generation

The citrate-anticoagulated PPP and S-2238 (Chromogenix) were used in this study^42^. Different coatings including PDA/PEG, HPG/PEG and SPI were applied to glass vials. Mixture of 80 μl of PPP, 20 μl of 6 mM CaCl_2_/normal saline (final concentration) and 20 μl of 4 mM substrate S-2238 was incubated with different coatings for 30 min at 37 °C under shaking. After incubation, the reaction was stopped by adding sodium citrate. Thrombin generation was detected by ultraviolet–visible (UV–vis) spectroscopy at 405 nm. PPP was obtained from at least three individuals, and each experiment was repeated independently at least three times.

### TAT complex generation

The TAT complex generation was evaluated by ELISA (Human TAT kit, Abcam) method^10^. Different coatings including PDA/PEG, HPG/PEG and SPI were applied to glass vials. Then, 80 μl of citrate-anticoagulated PPP was introduced and the coagulation was triggered by adding 20 μl of 6 mM CaCl_2_/normal saline (final concentration). The reaction was carried out for 30 min at 37 °C under shaking. After incubation, the reaction was stopped by adding sodium citrate. Then, the plasma samples were collected and the TAT concentration was tested according to the instruction manuals. PPP was obtained from at least three individuals, and each experiment was repeated independently at least three times.

### Plasma recalcification times

Different coatings including PDA/PEG, HPG/PEG and SPI were applied on glass vials and PS well plates. Then, 80 μl of citrate-anticoagulated PPP was introduced to each sample and the coagulation was triggered by adding 20 μl of 6 mM CaCl_2_/normal saline (final concentration). The reaction was carried out at 37 °C under shaking conditions^10^. Clotting times for each sample were recorded. If no thrombus formation was observed after 30 min, the experiment was terminated and the time of experiment was recorded.

For the PPP-clotting time under shear conditions, a closed loop was developed with silicone tubing (Tygon 3350 Sanitary Silicone Tubing) that is approximately 10 cm in length and an inner diameter of 2.4 mm. The closed loop was formed using a straight connector. The uncoated or SPI-coated tubing loop was placed in a peristaltic roller pump (WF300-TH16, PreFluid), and a PBS buffer was circulated through the loop for 10 min. Citrate-anticoagulated PPP (recalcified with 6 mM CaCl_2_, final concentration) was carefully introduced into the tubing and ensured that no air is introduced (this step is very important as air bubbles can cause severe contact activation). The loop was modelled to simulate flow in the carotid vein; the shear rate of the plasma was 60 s^−1^. The time of thrombus formation during the experiment was recorded (under 37 °C). If no thrombus formation was observed after 60 min, the experiment was terminated and the time of experiment was recorded. PPP was obtained from at least three individuals, and each experiment was repeated independently at least three times.

### aPTT, PT, TT and fibrinogen concentrations

A semiautomatic blood coagulation analyser (CA-50, Sysmex) was used to investigate the influence of SPI coating on the blood coagulation system^10^. Here 300 μl of fresh citrate-anticoagulated PPP was introduced into bare or SPI-coated glass vial and incubated at 37 °C for 30 min. For the aPTT test, 50 μl of the incubated PPP was moved to a test cup and mixed with 50 μl of the aPTT reagent (Dade Actin Activated Cephaloplastin Reagent, Siemens; incubated for 10 min at 37 °C before use). After incubating at 37 °C for 3 min, 50 μl of 25 mM CaCl_2_ solution was added, and then the aPTT was measured. For the TT test, 50 μl of the incubated PPP was added in a test cup and mixed with 100 μl of the TT reagent (Test Thrombin Reagent, Siemens; incubated for 10 min at 37 °C before use), and then the TT was measured. For the PT test, 50 μl of the incubated PPP was added in a test cup, followed by the addition of 100 μl PT reagent (Thromborel S, Siemens; incubated for 10 min before use), and then the PT was measured. For the detection of the concentration of fibrinogen in plasma, 10 μl of the incubated PPP was added in a test cup and diluted with 50 μl buffer. After incubating at 37 °C for 3 min, the complex solution was mixed with 50 μl thrombin reagent (Sysmex; incubated for 10 min at 37 °C before use), and then the fibrinogen was measured. For all the experiments, at least three parallel samples were applied to get a reliable value, and results were expressed as the mean ± s.d. (*n* = 3).

### Proteomic analysis of SPI-coating-incubated PPP

Fresh citrate-anticoagulated PPP (300 μl) was introduced into bare or SPI-coated glass vial. After incubating for 30 min, the plasma was collected. The sample was grinded with liquid nitrogen into a powder and then transferred to a 5 ml centrifuge tube. After that, four volumes of lysis buffer (8 M urea, 1% protease inhibitor cocktail) were added to the plasma powder, followed by sonication three times on ice using a high-intensity ultrasonic processor (Scientz). The cellular debris from the plasma sample was removed by centrifugation at 12,000*g* at 4 °C for 10 min. Then, the supernatant was transferred to a new centrifuge tube. The top-14 high-abundance proteins were removed by Pierce Top 14 Abundant Protein Depletion Spin Columns Kit (Thermo Fisher Scientific). Finally, the protein concentration was determined with a bicinchoninic acid kit according to the manufacturer’s instructions. Detailed analysis of protein digestion, mass spectrometry analysis and protein identification are given in the ‘Extended methods’ section in the Supplementary Information.

### Detection of the activities of coagulation factors in PPP

An automatic coagulation analyser (ACL Elite Pro, Werfen) was used to investigate the activities of coagulation factors after incubating recalcified PPP with a bare or SPI-coated glass vial. Fresh recalcified PPP (300 μl) was introduced into vials and incubated at 37 °C, and the reaction was stopped by adding sodium citrate after 30 min incubation^10^. For the detection of the activities of FVIII, FIX, FXI and FXII in plasma, 5 μl of the incubated PPP was added in a test cup and diluted with a 45 μl buffer solution. After incubating at 37 °C for 30 s, the complex solution was mixed with 50 μl of the corresponding factor-deficient plasma (coagulation FVIII-deficient plasma, coagulation FIX-deficient plasma, coagulation FXI-deficient plasma and FXII-deficient plasma; Sysmex; incubated for 10 min at 37 °C before use). After another 30 s incubation at 37 °C, 50 μl of the aPTT reagent was added and incubated at 37 °C for 3 min, followed by adding 50 μl of 25 mM CaCl_2_ solution, and then the factor activity was measured. For all the experiments, at least three parallel samples were measured to get a reliable value, and results were expressed as the mean ± s.d. (*n* = 3).

### Animal model

Thrombogenicity of the medical-grade PVC catheters before and after functionalization by an SPI coating were assessed using an ex vivo AV shunt model in the rabbit^43^. This study was conducted on at least four healthy male New Zealand rabbits aged approximately 4 months old and weighing about 2.5–3.5 kg (without sex preference). The rabbit was anaesthetized with 3% sodium pentobarbital (1 ml kg^–1^) through an injector placed in an ear vein. The rabbit left carotid artery and the right external jugular vein were isolated through a midline neck incision. Two indwelling needles (22G) were stabbed into the carotid artery and jugular vein, and the blood flow was temporarily blocked by the cover that was supplied with the indwelling needle. Then, bare or SPI-coated catheters were connected with two indwelling needles and the AV custom-built extracorporeal circulation was initiated. Animals had no systemic anticoagulation throughout the experiment. After 30 min, the extracorporeal circulation was terminated, and the blood flow rate through the catheters was checked every 10 min. The blood flow rate was calculated from the volume of outflow within preset time (15 s). After the experiment, the catheter was gently rinsed by normal saline and then all the liquid was pumped out by using a peristaltic pump (BT100 + YZ1515). The residual thrombus in the catheter was collected and weighted. The catheters before and after the treatment were fixed using freshly prepared glutaraldehyde (2.5 wt% in PBS) overnight. The samples were washed with a PBS solution, and subjected to a drying process by passing them through a series of graded alcohol–PBS solutions. Fixed samples were imaged using a field-emission scanning electron microscope (JSM-7500F, JEOL).

The clotting-time assays of the rabbits before and after the experiment were explored by an automatic blood coagulation analyser (CA-7000, Sysmex). For exploring the coagulation activation of the blood after the experiments, blood from the catheter before and after the experiment was dropped into a PS 96-well plate. The whole-blood-clotting time of the blood sample was recorded. It is worth noting that the test methods based on thrombin and fibrinolytic systems to assess coagulation activation are not applicable here, because although the blood from the catheter could still flow and be collected, the coagulation pathway might be already activated and cannot be inhibited by anticoagulants. At least four parallel sample groups were applied to get a reliable value, and results were expressed as the mean ± s.d.

### Proteomic analysis of tightly and loosely bound proteins

As both loosely bound and tightly bound proteins on the surface should have the potential to contribute to the antithrombotic function of the surface, the protein adsorption characteristics on the surface in plasma were explored using proteomics techniques. The loosely and tightly bound proteins on bare or SPI-coated glass vials were in situ digested by trypsin and analysed by liquid chromatography–tandem mass spectrometry; detailed methods are described in the ‘Proteomic analysis of SPI coating incubated PPP’ section^10^ in the Supplementary Information.

### Influence of SPI coating on FXII–PK reciprocal activation

The in vitro simulated contact initiation system contains 400 nM FXII and 400 nM PK (from Enzyme Research Laboratories) in a PBS buffer was incubated with glass vials before and after HPG/PEG and SPI modification for different intervals (3 min and 5 min). After the reciprocal activation of FXII and PK, 10 μl of the mixture was transferred to 90 μl of S-2302 substrate solution (2 mM in a PBS buffer) and p-nitroanilide (pNA) generation was detected by UV–vis spectroscopy at 405 nm in a kinetic model and at least three parallel sample groups were applied to get a reliable value. Reducing SDS–PAGE (12% acrylamide) was used to detect the products.

For further exploring the influence of SPI coating–FXII interaction on the contact system under the simulated plasma condition, C1INH (Sigma), a protease inhibitor widely present in blood, was introduced. Contact initiation system contains 400 nM FXII, 400 nM PK and 1 µM C1INH in a PBS buffer and was incubated with uncoated, HPG/PEG-coated and SPI-coated glass vials for different intervals (15 min and 30 min). After the reciprocal activation of PK and FXII, 10 μl of the mixture was transferred to 90 μl of S-2302 substrate solution (2 mM in a PBS buffer) and pNA generation was detected by UV–vis spectroscopy at 405 nm in the kinetic mode.

For exploring whether surface–FXII interaction can result in the activation of FXII, 400 nM FXII was first incubated with glass vials with and without HPG/PEG and SPI coatings for 10 min, and then the solution was transferred to a new PP tube and PK (final concentration, 400 nM) was added and co-incubated for 5 min. Thereafter, 10 μl of the mixture was transferred to 90 μl of S-2302 substrate solution (2 mM in a PBS buffer) and pNA generation was detected by UV–vis spectroscopy at 405 nm in the kinetic mode. At least three parallel sample groups were applied to get a reliable value. Reducing SDS–PAGE (12% acrylamide) was used to detect the products.

For exploring the surface–PK interaction on the function of PK, 400 nM PK was first incubated with uncoated, HPG/PEG-coated and SPI-coated glass vials for 10 min, and then the solution was transferred to a new PP tube and FXII (400 nM, final concentration) was added and co-incubated for 5 min. Thereafter, 10 μl of the mixture was transferred to 90 μl of S-2302 substrate solution (2 mM in a PBS buffer) and pNA generation was detected by UV–vis spectroscopy at 405 nm in the kinetic mode. At least three parallel sample groups were applied to get a reliable value. Reducing SDS–PAGE (12% acrylamide) was used to detect the products.

### Active dimethyl labelling and conformation analysis of FXII in plasma by mass spectrometry

Here 1 mg of SPI-coated or uncoated glass microspheres was incubated with 100 µl FXII solution (10 µg/100 µl of a PBS buffer) for 30 min under 37 °C. The FXII solution without surface incubation was used as a blank control. Then, 3.2 µl NaBH_3_CN (0.15 M) and 3.2 µl CH_2_O (1%) were added, and the reaction was carried out at 37 °C for 20 min. To stop the reaction, 4 µl NH_4_HCO_3_ (2.5 M) was added and incubated for 20 min. The mixture was centrifuged and the supernatant was collected. Subsequently, the labelled FXII protein present in the supernatant was subjected to chymotrypsin digestion following the instructions provided with the reagents (https://assets.fishersci.com/TFS-Assets/LSG/manuals/MAN0011638_Mass_SpectroGrade_Endoprotein_UG.pdf). Detailed information on mass spectrometry analysis and protein identification are given in the ‘Extended methods’ section in the Supplementary Information.

#### Statistical analysis

Most experiments were repeated for a minimum of three independent analyses unless mentioned otherwise. In general, data are expressed as the mean ± s.d. Statistical analysis was performed using GraphPad Prism 8. A difference with a *P* value of less than 0.05 was considered statistically significant. Data distribution was assumed to be normal but this was not formally tested. Data were statistically analysed with unpaired, two-tailed Student’s *t*-test (Figs. 2a and 4c and Supplementary Figs. 16, 20, 24 and 27); paired, two-tailed Student’s *t*-test (Fig. 4d,f); unpaired, one-way analysis of variance (ANOVA) followed by Tukey’s post hoc tests (Figs. 2b–e and 3a,f,g and Supplementary Figs. 12, 14, 15, 17–19, 21–23, 25, 28–30, 34 and 36); and paired, one-way ANOVA (Fig. 4b). In substrate course analyses, a mixed two-way ANOVA with Bonferroni’s post hoc tests were used (Fig. 2a). No statistical methods were used to predetermine the sample sizes, but our sample sizes are similar to those reported in previous publications^10,11,23,38,44^. For Figs. 1–3, 5 and 6 and the associated supplementary figures, data collection and analysis were not performed blind to the conditions of the experiments. For Fig. 4 and the associated supplementary figures, rabbits were randomly assigned to experimental groups, and operators were blinded to the group assignments. No animals or data points were excluded from the analyses. All results were consistent across independent experiments. The findings in this study were replicated independently with different groups, and the data collection was randomized.

### Reporting summary

Further information on research design is available in the Nature Portfolio Reporting Summary linked to this article.

## Online content

Any methods, additional references, Nature Portfolio reporting summaries, source data, extended data, supplementary information, acknowledgements, peer review information; details of author contributions and competing interests; and statements of data and code availability are available at https://doi.org/10.1038/s41563-024-02046-0.

## Supplementary information


Supplementary InformationSupplementary Figs. 1–42, Tables 1–5, caption for Supplementary Video 1, results, methods, discussions and references.
Reporting Summary
Supplementary Video 1SPI coating can prevent the glass-vial-induced coagulation activation.
Supplementary Data 1Supplementary data for Supplementary Figures.


## Source data


Source Data Figs. 1–6Source data for Figs. 1–6.


## Data Availability

Protein mass spectra were searched against the human SwissProt database (20,422 entries) concatenated with the reverse decoy database (proteome ID: UP000005640; release nos. 2021_01/2021_01). Proteomic data, including raw data and search results, have been deposited in the ProteomeXchange database with dataset identifier PXD054476 (for Figs. 3b and 5b,c) and PXD054293 (for Fig. 6f). All other data supporting the findings of this study are available within the Article and its Supplementary Information and available from the corresponding authors on request. Source data are provided with this paper.
