# Supplementary material for: Antithrombotic coating with sheltered positive charges prevents contact activation by controlling factor XII–biointerface binding
Source: Nat Mater. 2024 Nov 12;24(4):626–36. doi: 10.1038/s41563-024-02046-0 (PMC11961369; doi:10.1038/s41563-024-02046-0)
Supplement: Supplementary file 1 — Supplementary Figs. 1–42, Tables 1–5, caption for Supplementary Video 1, results, methods, discussions and references. [file 41563_2024_2046_MOESM1_ESM.pdf]

nature materials

Supplementary information

https://doi.org/10.1038/s41563-024-02046-0

Antithrombotic coating with sheltered positive charges prevents contact activation by controlling factor XII–biointerface binding

In the format provided by the authors and unedited

Table of Contents

Supplementary videos legends: **2**
Supplementary methods: **2**
Exploration of coagulation activation induced by materials with distinct surface properties 2
Chemicals and substrates 2
Chemical characterization methods 2
Preparation of epoxy-PEG_{350}-OMe 2
Preparation of epoxy-PEG_{400}-azide 3
Preparation of HPG-PEG 3
Preparation of UHRA-10 4
Preparation of primary amine functionalized HPG-PEG (Amine-HPG-PEG) 5
Preparation of heparin coating 5
PDA/PolyN,N-dimethylacrylamide (PDMA) coating preparation 5
ATR-FTIR 5
X-ray photoelectron spectroscopy (XPS) 5
Quartz crystal microbalance (QCM) analysis 6
Ellipsometry 6
Scanning electron microscopy (SEM) 6
Atomic force microscopy (AFM) 6
Water contact angle (WCA) measurements 6
Zeta potential measurements 7
Plasma recalcification times 7
Comparison of PDA/PDMA and SPI coating in antithrombotic functionality 7
Long-term stability of the coating 7
Proteomic analysis of SPI coating incubated PPP 8
Detection of the activation of FVII and fibrinolytic system 9
Confocal analysis for platelet adhesion on glass chamber 9
Lactate dehydrogenase (LDH) and SEM analyses for platelet adhesion under different shear rates 9
Platelet activation using flow cytometry 10
Confocal microscopy analysis for neutrophil adhesion 10
Neutrophil activation using flow cytometry 10
Cell adhesion and cytotoxicity measurements 11
Hemolysis 11
Blood count assay *in vitro* 12
C3a and C5b-9 generation 12
Sheep erythrocyte based hemolytic assay 12
Additional information on animal experiments 13
Blood flow simulation using computational fluid dynamics (CFD) mode 13
Wash protocol and measurement of surface protein concentration after washing 13
QCM analysis of protein adsorption on SPI coating from plasma 13
Cy5 labeling of FXII 14
In situ FXII adsorption detection by flow cytometry 14
Surface influence of FXII-SPI coating reciprocal activation 14
Active dimethyl labelling and conformation analysis of FXII in plasma by mass spectrometry 15
Supplementary Results and Extended Discussion: **16**
Supplementary results for coating characterization: 16
Supplementary results on the hemocompatibility of SPI coating: 16
Supplementary results on coating-FXII interaction: 16
Supplementary results on mechanism of modulation of the contact activation: 17
Supplementary extended outlook: 18
Supplementary figures: **20**
Supplementary tables: **50**
Supplementary references: **57**

Supplementary videos legends:

SPI coating can prevent the glass vial induced coagulation activation.

Supplementary methods:

Exploration of coagulation activation induced by materials with distinct surface properties

All chemicals are purchased from Sigma-Aldrich Canada (Oakville, Ontario) without purification for finding the concepts guiding the design of new antithrombogenic surfaces. Development of various surfaces with distinct surface properties including glass (3 μm), polyethyleneimine (PEI) coated glass, titanium (5 μm), polymelamine (5 μm), polystyrene (PS) (7 μm), gold (100 nm) and PEG modified gold in their nanoparticulate form.

The substrates in their nanoparticulate form were incubated with citrate anticoagulated plasma (0.5 mg of glass, PEI coated glass, titanium, polymelamine, or PS per 200 μL of PPP, or 50 μg of gold and PEG modified gold per 200 μL of PPP) for 10 min, then the incubated plasma was collected and recalcified (final concentration: 10 mM) to trigger the coagulation. 100 μL of the mixture was transferred to a 96-well plate immediately and the optical density was detected at 405 nm in a plate reader. Each experiment was repeated independently at least three times and the plasma of 3 individuals was used.

Chemicals and substrates

Chemicals are purchased from Sigma-Aldrich, Canada (Oakville, Ontario) without purification if not specified in the subsequent experimental procedures. Water was purified using a Milli-Q Plus water purification system (Milipore Corp., Bedford, MA) and used in all experiments. Single-side-polished silicon wafers were bought from University Wafer (Boston, MA). Polypropylene (PP) sheet with a thickness of 0.3 mm was purchased from Professional Plastics (CA, USA). Silicone and polyvinyl chloride tubing for platelet adhesion experiments were products from Cole-Parmer (Canada) and Amazon (Canada) respectively. Polyvinyl chloride catheters for animal experiment were products from Alibaba (China). Indwelling needles (22 G) were purchased from Braun Melsungen AG (Germany).

Chemical characterization methods

^{1}H nuclear magnetic resonance (HNMR) spectra were acquired in deuterium oxide or chloroform-D ($CDCl_3$, Cambridge Isotope Laboratories, Inc. USA) on a Bruker Avance AV-300 spectrometer. Absolute molecular weights of the polymers were determined by Gel Permeation Chromatography (GPC) on a Waters 2695 separation module fitted with a DAWN EOS multiangle laser light scattering (MALLS) detector coupled with Optilab DSP refractive index detector, both from Wyatt Technology. GPC analysis was performed using Waters ultrahydrogel columns (guard, linear and 120) and 0.1 M $NaNO_3$ (10 mM phosphate buffer) as the mobile phase. The Attenuated total reflectance-Fourier transform infrared spectroscopy (ATR-FTIR) spectra were collected using a Bruker 670 TensorII with an MCT/A liquid nitrogen cooled detector, a KBr beam splitter, and a VariGATR grazing angle accessory. Spectra were recorded at 2 cm^{-1} resolution, and 128 scans were collected for each sample.

Preparation of epoxy-PEG_{350}-OMe

In an ice bath, 220 g of MeO-PEG_{350}-OH and 74 g of NaOH were charged into the flask under the agitation condition for 3 h. Then, 125 g of the epichlorohydrin was added to the flask dropwise (0.75-1g/min). After the addition of the epichlorohydrin, ice bath was removed and the reaction was kept for 3 days under room temperature (RT). After the reaction, 200 mL of dichloromethane (DCM) was added to the flask. The suspension

was filtered and the filtrate was collected containing epoxy-PEG_{350}-OMe. To the filtrate, 15 g of anhydrous $MgSO_4$ was added. The flask was re-sealed by a glass stopper and stirred for 5 h. Then, the mixture was filtered again to remove $MgSO_4$, and a rotary evaporator was used to remove the DCM. Then the epoxy-PEG_{350}-OMe was dried for another 5 h under vacuum at 80 °C, then molecular sieves (4 A, Davison, Fisher Scientific) were added to the epoxy-PEG_{350}-Me under the protection of argon.

^{1}H NMR ($CDCl_3$, 300 MHz): δ ppm 3.78 (dd, J = 11.7, 3.1 Hz, 1H), 3.73-3.51 (m, 33H), 3.16 (dq, J = 6.1, 3.1 Hz, 1H), 2.79 (t, J = 4.6 Hz, 1H), 2.61 (dd, J = 5.0, 2.7 Hz, 1H) (peaks from epoxy group), no water peak was observed.

Preparation of epoxy-PEG_{400}-azide

PEG_{400} (0.027 M) (10.9 g) was dried under vacuum at 70 °C overnight and then dissolved in 200 mL of DCM. After making a homogeneous solution, 2 mL of triethylamine was added to the solution. The solution was cooled down in ice, then 1.875 g of p-toluenesulfonyl chloride (TsCl) (0.01 M, ACROS) dissolved in 50 mL of DCM solution was added dropwise to the PEG_{400} solution for 15 h in ice, and the reaction was kept for 36 h under RT. After that, the solution was washed with DI water (150 mL, three times) and saturated NaCl aqueous solution (200 mL). The resulting solution was concentrated by rotary evaporation, then the product was dried by freeze-drying for 24 h to give the OH-PEG_{400}-TsCl, which was then dissolved in 100 mL of dimethyl formamide (DMF). After being completely homogeneous, 3.6 g of NaN_3 (0.055 M) was added to the solution and the reaction was carried out at 60 °C for 24 hrs. The resulting solution was concentrated by rotary evaporation and re-dissolved in ethyl acetate, then the solution was filtered to remove the solid sodium tosylate and concentrated by rotary evaporation. The resulting oily product was dissolved in water and washed with hexane until diazido PEG was not detected in the hexane solution. Then the solution was dried by rotary evaporation, gave the product (OH-PEG_{400}-azide) as a light red oily liquid (yield: 4.1 g).

3.17 g of OH-PEG_{400}-azide (8 mM) was dried under vacuum at 50 °C overnight, then dissolved in 60 mL of anhydrous tetrahydrofuran (THF). Then, 0.4 g of NaH (16 mM) was added to the solution and the reaction was carried up at 60 °C for 4 h under the protection of argon. After that, 1.1 mL of epichlorohydrin (14 mM) was added to the solution dropwise for 10 min, and the reaction was kept at 60 °C overnight under the protection of argon. The reaction solution was filtered and concentrated by rotary evaporation. After that, the product was dissolved in 150 mL of DCM solution, and which was washed with water (150 mL, three times). Then the solution was dried by rotary evaporation and further dried under vacuum at 70 °C overnight. Finally, the product (epoxy-PEG_{400}-azide, yield: 2.4 g) was stored under argon protection.

^{1}H NMR ($CDCl_3$, 300 MHz): δ ppm 3.78 (dd, J = 11.7, 3.1 Hz, 1H), 3.73-3.51 (m, 33H), 3.16 (dq, J = 6.1, 3.1 Hz, 1H), 2.79 (t, J = 4.6 Hz, 1H), 2.61 (dd, J = 5.0, 2.7 Hz, 1H) (peaks from epoxy group), 3.37 (protons near azido group), no water peak was observed, as shown in **Supplementary Fig. 2a.**

Preparation of HPG-PEG

Three-necked flask was dried by flame under vacuum and filled with argon (3 times). Then 1,1,1-tris(hydroxymethyl)propane (TMP) (480 mg, 3.6 mM) was added into the flask and melt at 70 °C, and 440 μL of potassium methylate (25 wt% in methanol) was added to the flask at 70 °C for 15 min under stirring, then the methanol was removed by vacuum for 4 hrs. The flask was heated to 95 °C and distilled glycidol (7.5 mL, 113 mM) was added with argon protection over a period of 15 h under stirring, then the mixture was stirred for additional 3 h at 95 °C. After that, 15.5 mL of epoxy-PEG_{350}-Me (44 mM) was added with argon protection over a period of 12 h under stirring. Then the mixture was stirred for additional 4 h at 95 °C. After the reaction, the product was dissolved in 30 mL of methanol, and then twice precipitated from 500 mL of cold diethyl ether. The

sediment was collected by centrifuge, and further dried by rotary evaporation. The result was dissolved in DI water and dialyzed against DI water using MWCO-1000 membrane (Repligen) for 2 days with periodic changes in water. Then the solution was dried by lyophilizer to give the HPG-PEG.

GPC-MALLS (0.1 M $NaNO_3$): Mn 10,000 g/mol; Mw/Mn 1.4. ^{1}H NMR ($CDCl_3$, 300 MHz): δ ppm 3.37 (s, -O-CH_3 from PEG), 3.4-3.95 (main chain protons from PEG and HPG), PEG content: 31 mol%; Polyglycerol: 69 mol%.

Preparation of UHRA-10

The synthesis of UHRA-10 can be referred in our previous report[1]. Briefly, HPG-PEG (2.29 g) was dried under vacuum at 60 °C overnight, then 50 mL of dry pyridine was added. To this, 650 mg of TsCl was added and stirred at RT for 24 hrs. Then the pyridine was removed by rotary evaporation. The polymer was dissolved in 10 mL of HCl (0.1 M) solution, and dialyzed against DI water using MWCO-1000 membrane for 1 day. The HPG-PEG-tosylate was dried by freeze drying. Then the HPG-PEG-tosylate and 10 mL of tris (2-aminoethylamine) were dissolved in 50mL of 1,4-dioxane and refluxed for 24 h at 105 °C under stirring. Then the dioxane was removed by rotary evaporation and the polymer was dissolved in 5 mL of methanol and precipitated twice in 400 mL of cold diethyl ether. The sediment was collected by centrifuge, and further dried by rotary evaporation. The polymer was dissolved in 50 mL of water and dialyzed against DI water using MWCO-1000 membrane for 8 hrs. Then a mixture of formaldehyde (12 mL) and formic acid (12 mL) was added to the resulting polymer solution, and the reaction mixture was refluxed overnight at 100 °C. After cooling to room temperature, the pH of the solution was adjusted to 10 using NaOH solution and the polymer was extracted with DCM. The solvent was removed by rotary evaporation, and the resulting polymer was dissolved in distilled water and dialyzed against DI water using MWCO-1000 membrane with frequent changes in water for 2 days. Then the solution was dried by lyophilizer to give the SPCM. The number of methylated tris(2-aminoethylamine) group conjugated on the HPG-PEG polymer was determined by conductometric titration [2]. The number of methylated tris(2-aminoethylamine) in UHRA-10 (by conductometric titration) was calculated to be 11, the positive charged groups were calculated to be 44.

^{1}H NMR ($CDCl_3$, 300 MHz): δ ppm 2.27 (s, -NCH_3), 2.4-2.7 (m, -N-CH_2-), 3.37 (s, -O-CH_3), 3.4-3.95 (main chain protons from PEG and HPG, m, 35H).

Synthesis of sheltered positive charged macromolecule (SPCM)

UHRA-10 (1 g) and 200 mg of epoxy-PEG_{400}-azide (~5 mole equ. of UHRA-10) were dried under vacuum at 60 °C overnight severally. Then the UHRA-10 was dissolved in 30 mL of anhydrous THF at 70 °C under argon, and 200 mg of NaH was added to the solution and the reaction was kept under stirring at 60 °C for 10 h under argon. After that, 200 mg of epoxy-PEG_{400}-azide was dissolved in 20 mL of anhydrous THF, and the solution was added to the reaction solution under argon protection over a period of 10 min under stirring, then the reaction was maintained at 75 °C for 24 hrs. The resulting solution was concentrated by rotary evaporation and dissolved in 10 mL of DI water, the product was purified by dialysis (molecular cut-off: 2000) for 24 hours, during which the water was changed three times. The solution was dried by lyophilizer to give the azide modified UHRA-10 (A-UHRA-10). FTIR: 2100 cm^{-1} (peak of azido group, **Fig. S4a**). ^{1}H NMR are given in **Fig. S3b.**

After that, 1 g of A-UHRA-10 was dried under vacuum at 60 °C overnight and then dissolved in 80 mL of THF. Then, 400 mg of triphenylphosphine (PPh) was added to the reaction and after that 40 mL of DI water was added to the solution under stirring for 48 h at 50 °C. After the reaction, the solution was concentrated by rotary evaporation and dissolved in 100 mL of DI water, and washed with 100 mL ethyl acetate twice to remove unreacted PPh and oxidized PPh. The resulting water solution was purified by dialysis (molecular cut off: 1000)

for 24 h, during which the water was changed three times. The solution was dried by lyophilizer to give the final primary amine modified SPCM. FTIR: peak of 2100 cm^{-1} disappeared.

Preparation of primary amine functionalized HPG-PEG (Amine-HPG-PEG)

The synthesis of amino-HPG-PEG is similar to that of SPCM, differ in replace SPCM with HPG-PEG. The success synthesis of amino-HPG-PEG was proved by FTIR. The amine-HPG-PEG was used to grafted onto substrate surface as a control group for surface and blood analyses. FTIR: peak of 2100 cm^{-1} disappeared.

Preparation of heparin coating

The coating is stable under physiological condition and can combine with antithrombin III in recalcified platelet poor plasma. Briefly, the glass vials were first immersed in mix solution (2 mg of dopamine, 5.3 mg of sodium periodate, and 20 mg of deacetylated chitosan per 1 mL of sodium acetate buffer (ph~5.4)) for 30 min to generate the PDA-chitosan coating (zeta potential in pH~7: ~-1 mV). Then, the coated surface was immersed in unfractionated heparin solution (1 mg of heparin per 1 mL of DI water) for 1 h to generate the heparin coating (zeta potential in pH~7: ~-22 mV, heparin coating density calculated by QCM measurement: 71.7 ng/cm^2). The coating was carefully washed with normal saline for several times to remove free heparin.

Synthesis of PDA/PEG coating, SPI coating, HPG-PEG coating, and PEI coating

Take glass substrate for example, the glass was immersed in dopamine/PEG coating solution (2 mg/mL dopamine, 10 mg/mL of MeO-PEG-OH (5000 Da) and 5.3 mg sodium periodate/mL sodium acetate buffer solution (pH=5)) for 20 min (for glass vials) or 10 min (for glass microspheres). Afterwards, the substrate was washed with DI water to get the PDA/PEG coating[3].

For the SPI coating, the PDA/PEG coated glass was immersed in SPCM solution (0.5 mg/mL in PBS, pH=8.5) under RT for 24 hrs. For the HPG-PEG coating and PEI coating, the PDA/PEG coated substrate was immersed in amine-HPG-PEG or PEI (Mn~10000 Da) solution (0.5 mg/mL in PBS, pH=8.5) under RT for 24 hrs. The HPG-PEG coating and PEI coating were used as control group for blood tests. All the coated surfaces are washed and stored in PBS buffer.

PDA/PolyN,N-dimethylacrylamide (PDMA) coating preparation

The ultra-high molecular weight PDMA was synthesized by aqueous ATRP as detailed in our previous work[4]. The synthesized PDMA has a Mw of 702,000 and a polydispersity of 1.38 (Mw/Mn). For preparing PDA/PDMA coating, the glass vials were immersed in dopamine/PDMA coating solution (2 mg/mL dopamine, 30 mg/mL of PDMA and 5.3 mg/mL of sodium periodate dissolved in sodium citrate buffer solution (pH=5)) for 20 min. After the coating preparation, the vials were washed thoroughly with water.

ATR-FTIR

PP film was chosen as the substrate in this experiment. The ATR-FTIR spectra were collected using a Bruker 670 TensorII with an MCT/A liquid nitrogen cooled detector. Spectra were recorded at 2 cm^{-1} resolution, and 128 scans were collected for each sample. To test the stability of the coating in physiological environment. The coated PP film was respectively treated by 3M NaCl and 0.3 M urea solution under RT for 1 day. The spectrum of the coating after treatment was collected (**Fig. S5**).

X-ray photoelectron spectroscopy (XPS)

Single-side-polished silicon wafer was chosen as the substrate in this experiment. XPS analysis was performed using a Leybold LH Max 200 surface analysis system (Leybold, Cologne, Germany) equipped with a Mg Kα

source at a power of 200 W. Elements were identified from survey spectra. High resolution spectra were collected at 48 eV pass energy (**Fig. S6**).

Quartz crystal microbalance (QCM) analysis

QCM-D (Biolin Scientific) was used for evaluation of the mass and stability of the coating on the sensor surface. The PDA/PEG coated gold sensor (QSX 301, nano science instruments) were placed into the titanium flow chamber. PBS buffer was pumped over the sensor surface for 1 h, and baseline equilibrium was reached. Then the solution was switched to SPCM solution (concentration 0.5 mg/mL), and the reaction was kept for 24 h. And the sensor was washed by PBS solution until getting a horizontal line. Then the solution was switched to 3 M NaCl solution, and the wash step was kept until getting a horizontal line. Followed by a PBS rinse for 30 min. Then the solution was switched to 0.3 M Urea solution, and the wash step was kept until getting a horizontal line. Followed by a PBS rinse for 30 min. The coating mass was analyzed by Q-Sense Dfind.

Ellipsometry

The coating thickness was obtained from ellipsometer (M-2000V, J.A. Woollam Co., Inc.). The bare wafer was cleaned by the oxygen plasma treatment, and immersed in MeOH for 30 min and dried by argon, then the thickness of the bare wafer was measured. After that, the wafer was first coated with PDA/PEG, and immersed in PBS solution and sonicated for 5 min, and immersed in MeOH and then dried by argon. Then the thickness of the PDA/PEG coating was measured. Next, the wafer was coated with SPCM, and immersed in PBS solution and sonicated for 5 min, and immersed in MeOH and then dried by argon. Then the thickness of the SPI coating was measured. In order to verify whether the coating is stable and bonded to the surface, the SPI coating after wash step was immersed in 3 M of NaCl solution overnight, and immersed in MeOH and then dried by argon. Then the thickness of the NaCl solution treated SPI coating was measured. Then SPI coating was immersed in 0.3 M of Urea solution overnight, and immersed in MeOH and then dried by argon and the thickness of the SPI coating was measured. In order to further explore the stability of the coating, the SPI coated substrate was stored in an antibacterial solution (0.3 M of NaN_3) for 7 days, and immersed in MeOH and then dried by argon. Then the thicknesses of the SPI coating were measured in each case.

Scanning electron microscopy (SEM)

Single-side-polished silicon wafer was chosen as the substrate in this experiment and coated with PDA/PEG or SPI coating. The surface-morphologies were obtained by scanning electron microscopy (SEM, Phenom Pure, Phenom World, Netherlands.

Atomic force microscopy (AFM)

Single-side-polished silicon wafer was chosen as the substrate in this experiment and coated with PDA/PEG or SPI coating. AFM measurements were performed using a commercially available multimode system with a scan range of 2×2 μm^2, controlled by a NanoScope IIIa controller (Digital Instruments, Santa Barbara, CA). Surface morphology and atomic force spectroscopy was examined under PBS buffer in contact mode using a commercially manufactured V-shaped silicon nitride (Si_3N_4) cantilever with gold on the back for laser beam reflection (Veeco, NP-S20).

Water contact angle (WCA) measurements

For the WCA analyses, the coatings were applied on both silicon wafer and PP film. A water droplet (6 μL) was placed on the surface and an image of the droplet was taken with a digital camera (Retiga 1300, Q-imaging Co.). The contact angle was analyzed using Northern Eclipse software. Over three different sites were tested for each

sample.

Zeta potential measurements

The surface zeta potentials of the coated substrates were measured using a Zetasizer Nano-ZS90 (Malvern Instruments Ltd., UK). In this work, polystyrene macroparticles (7 μm) were used as substrate. Then the coated macrospheres were dispersed to make a 1 mg/mL suspension in pH~7. The zeta potential measurements were carried out at RT. To verify the interaction between SPI coating and negatively charged heparin, SPI coated PS microspheres were incubated with heparin solution (200 IU/mL of PBS buffer (150 mM)) for 4 h, then the microspheres were collected and used for zeta potential measurements. The zeta potential of SPCM is measured in SPCM solution (10 mg of SPCM per 1 mL of DI water, pH~7).

Plasma recalcification times

A series of experiments were designed to verify the sustained antithrombotic effect of the coating. Typically, bare glass vials and SPI, heparin, PDA/PEG-coated glass vials were used as test samples. 100 μL of citrate-anticoagulated PPP was introduced to each sample, then the coagulation was triggered by adding 10 μL of 6 mM $CaCl_2$/normal saline (final concentration). The time of thrombus formation during the experiment was recorded (under 37 °C). If there was no thrombus formation after 30 min, the experiment was terminated and recorded the time of experiment. Then, PPP in samples without thrombus formation was gently aspirated, and fresh recalcified PPP was added to these samples and repeat the above process.

To explore the influence of SPI coating on the blood coagulation system especially the intrinsic coagulation pathway, the recalcified PPP was added to SPI-coated vials and incubated for 30 min (no thrombus formation), then 100 μL of activated partial thromboplastin time (aPTT) reagent (Dade Actin Activated Cephaloplastin Reagent, Siemens) was added to trigger the coagulation, and the clotting time was recorded.

Comparison of PDA/PDMA and SPI coating in antithrombotic functionality

The antithrombotic function of the different samples (uncoated glass vial, PDA/PDMA coated glass vial, and SPI coated glass vial) and their effects on contact system were explored. For the exploration of the antithrombotic function, 100 μL of recalcified PPP was co-incubated with the samples at 37°C under shaking condition, and the clotting time was recorded. For exploring the effects on contact system, PK/FXII mixture solution (final concentration: 3 μg of PK and 3 μg of FXII in 100 μL of PBS buffer) was co-incubated with the samples. After the incubation, 5 μL of the solution was added to 50 μL of S-2302 substrate solution (2 mM in PBS buffer) and pNA generation was detected by UV-VIS at 405 nm in kinetic model, at least 3 parallel sample groups were applied to get a reliable value.

Long-term stability of the coating

Glass vials coated with SPI coating and uncoated glass vials were immersed in PBS at 4°C for one month. Then the antithrombotic function and their effects on contact system were explored. For the exploration of the antithrombotic function, 100 μL of recalcified PPP was co-incubated with the storage samples at 37°C under shaking condition, and the clotting time was recorded. For exploring the effects on contact system, prekallikrein (PK)/FXII (Enzyme research laboratories) mixture solution (final concentration: 3 μg of PK and 3 μg of FXII in 100 μL of PBS buffer) was co-incubated with the storage samples for 3 min. After the incubation, 5 μL of the solution was added to 50 μL of S-2302 substrate solution (2 mM in PBS buffer) and pNA generation was detected by UV-VIS at 405 nm in kinetic model, at least 3 parallel sample groups were applied to get a reliable value.

To detect the functional stability of the coating after incubating with plasma, glass vials coated with SPI coating and uncoated glass vials were first incubated with citrated PPP for 4 h. Then the antithrombotic function of the samples and their effects on contact system were explored. For the exploration of the antithrombotic function, 100 μL of recalcified PPP was co-incubated with the samples (with or without plasma incubation in last step) at 37 °C under shaking condition, and the clotting time was recorded. For exploring the effects on contact system, PK/FXII mixture solution (final concentration: 3 μg of PK and 3 μg of FXII in 100 μL of PBS buffer) was co-incubated with the samples (with or without plasma incubation in last step). After the incubation, 5 μL of the solution was added to 50 μL of S-2302 substrate solution (2 mM in PBS buffer) and pNA generation was detected by UV-VIS at 405 nm in kinetic model, at least 3 parallel sample groups were applied to get a reliable value.

Proteomic analysis of SPI coating incubated PPP

Three hundred microliters (300 μL) of fresh PPP citrate-anticoagulated was introduced into bare or SPI-coated glass vial. After incubating for 30 minutes, the plasma was collected. The sample was grinded with liquid nitrogen into powder and then transferred to a 5-mL centrifuge tube. After that, four volumes of lysis buffer (8 M urea, 1% protease inhibitor cocktail) were added to the plasma powder, followed by sonication three times on ice using a high intensity ultrasonic processor (Scientz). The cellular debris from plasma sample was removed by centrifugation at 12,000 g at 4 °C for 10 minutes. Then, the supernatant was transferred to a new centrifuge tube. The top 14 high abundance proteins were removed by Pierce™ Top 14 Abundant Protein Depletion Spin Columns Kit (ThermoFisher Scientific). Finally, the protein concentration was determined with BCA kit according to the manufacturer's instructions.

For digestion, the protein solution was reduced with 5 mM dithiothreitol for 30 min at 56 °C and alkylated with 11 mM iodoacetamide for 15 min at room temperature in darkness. The protein sample was then diluted by adding 100 mM TEAB to urea concentration less than 2 M. Finally, trypsin was added at 1:50 trypsin-to-protein mass ratio for the first digestion overnight and 1:100 trypsin-to-protein mass ratio for a second 4 h-digestion. Finally, the peptides were desalted by C18 SPE column. The tryptic peptides were dissolved in solvent A (0.1 % formic acid, 2 % acetonitrile/in water), directly loaded onto a home-made reversed-phase analytical column (25-cm length, 75/100 μm i.d.). Peptides were separated with a gradient from 4 % to 20 % solvent B (0.1 % formic acid in acetonitrile) over 96 minutes, 20 % to 32 % in 18 min and climbing to 80 % in 3 min then holding at 80 % for the last 3 min, all at a constant flow rate of 450 nL/min on an EASY-nLC 1200 UPLC system (Thermo Fisher Scientific).

The peptides were subjected to capillary source followed by the timsTOF Pro (Bruker Daltonics) mass spectrometry. The electrospray voltage applied was 1.60 kV. Precursors and fragments were analyzed at the TOF detector, with a MS/MS scan range from 100 to 1700 m/z. The timsTOF Pro was operated in a parallel accumulation serial fragmentation (PASEF) mode. Precursors with charge states 0 to 5 were selected for fragmentation, and 10 PASEF-MS/MS scans were acquired per cycle. The dynamic exclusion was set to 30 s.

The resulting MS/MS data were processed using MaxQuant search engine (v.1.6.15.0). Tandem mass spectra were searched against the human SwissProt database (20422 entries) concatenated with reverse decoy database. Trypsin/P was specified as cleavage enzyme allowing up to 2 missing cleavages. The mass tolerance for precursor ions was set as 20 ppm in first search and 5 ppm in main search, and the mass tolerance for fragment ions was set as 0.02 Da. Carbamidomethyl on Cys was specified as fixed modification, and acetylation on protein N-terminal and oxidation on Met were specified as variable modifications. FDR was adjusted to < 1%. Percent abundance for each protein was calculated using label-free quantification (LFQ) intensities relative to the total sum of protein LFQ intensities for each group. Relative abundance (R) of proteins in different samples was obtained by centralizing the LFQ intensity (I) of protein in different samples. The calculation formula is as follows:

$R_{ij}= M_{ij}/Mean (M_j)$, $M_{ij}= I_{ij}/Median (I_i)$

where i represents the sample and j represents the protein.

Detection of the activation of FVII and fibrinolytic system

Plasma with synergistic anticoagulation of hirudin (final concentration: 300 IU/mL) and sodium citrate (3.8 % sodium citrate with anticoagulant/blood ratio of 1:9) was used in this study. The plasma was then recalcified (final concentration: 10 mM) and incubated with different samples (PEI (Mw:10000, final concentration: 50 μg/mL), glass vial, glass vial coated with SPI coating, PT reagent (Stago, 10 μL/100 μL plasma, used as positive control)) for 30 min under 37 °C, plasma without recalcification was used as blank control. Afterwards, the FVIIa level for each sample was measured by Human Factor VIIa Chromogenic Activity Assay Kit (ab137995).

Plasma with synergistic anticoagulation of hirudin (final concentration: 300 IU/mL) and sodium citrate (3.8 % sodium citrate with anticoagulant/blood ratio of 1:9) was used in this study. The plasma was then recalcified (final concentration: 10 mM) and incubated with different samples (PEI coated glass via, glass vial, glass vial coated with SPI coating) for 30 min under 37 °C, plasma without recalcification was used as blank control. For the positive control, the citrated-plasma was recalcified and the obtained clot was treated with 200 nM plasmin for 30 min. Afterwards, the FDP level for each sample was measured by FDP ELISA Kit (Abbexa Ltd.).

Confocal analysis for platelet adhesion on glass chamber

To test the adhesion and activation of platelets on the surface, 200 μL of citrate-anticoagulated PRP was introduced into bare or SPI-coated 8-well-glass-chamber (eppendorf) and incubated for 30 min under shaking condition. After that, the surfaces were gently washed by PBS (avoid completely dry) and staining solution was introduced (mixture of 5 μL of anti-CD42-fluorescein isothiocyanate (FITC, BD, biosciences) and 5 μL of anti-CD62P-phycoerythrin (PE, BD, biosciences) to 90 μL of citrate-anticoagulated PPP) and incubated for 30 min at room temperature away from light. Following incubation, the surface was gently washed with stain buffer (BSA) (BD, biosciences). Then, the buffer was removed and the surface was fixed with 2.5 wt. % glutaraldehyde in PBS solution at 4 °C overnight. The platelet adhesion and activation on the surface was measured by III spinning disk confocal microscope. Images from the same batch with the same brightness and contrast were compared, the fluorescence intensity was measured by imageJ 1.53t. Each experiment was repeated at least three times.

Lactate dehydrogenase (LDH) and SEM analyses for platelet adhesion under different shear rates

For exploring the platelet adhesion on SPI-coated substrate under different shear rates, a closed loop was developed with two sections of SPI-coated PVC tubing (2 cm in length and an inner diameter of 2.4 mm) randomly nested within two sections of SPI-coated silicone tubing (2 cm in length and an inner diameter of 2.4 mm) and bare silicone tubing (5 cm in length and an inner diameter of 2.4 mm) as the creeping segment, and sterilized PBS buffer was circulated through the loop for 10 min). Citrate-anticoagulated PRP was carefully introduced into the tubing and ensured that no air is introduced (this step is very important as air bubbles can lead to severe platelet activation and protein denaturation). We explored the platelet adhesion behaviors under the shear rate of 60 s^{-1} (carotid vein) and 220 s^{-1} (carotid artery). After 1 h of the circulation, the PRP was gently removed and the sample sections were gently washed with PBS. Platelet adhesion on the sample sections was quantified using LDH assay[5]. The edges of the substrate were removed before the analysis. The adherent platelets were then lysed with 1.8% v/v Triton X-100 in water for 1 h at 37 °C. The LDH activity of the supernatant was measured following the protocol given with the commercial kit (LDH-cytotoxicity colorimetric assay kit, Biovision). Each experiment was repeated independently at least three times and the results were expressed as mean ± SD.

After circulation (the same test condition as above), the sample sections were gently washed with PBS and treated with 2.5 wt. % glutaraldehyde in PBS solution at 4 °C for 1 day. Then, the samples were washed by PBS solution and subjected to a drying process by passing them through a series of graded alcohol-DI water solutions (30, 50, 70, 80, 90, 95 and 100%). Platelet adhesion was observed using a FE-SEM (JSM-7500F, JEOL, Japan).

Platelet activation using flow cytometry

To test the platelet activation after incubating with coating, 200 µL of citrate-anticoagulated PRP was introduced into bare or SPI-coated glass vials and incubated for 30 min under shaking condition, thrombin receptor activating peptide was used as positive control (Sigma, final concentration: 0.1 mM). After that, the incubated PRP was added into the corresponding antibody-containing PPP solutions (mixture of 5 µL of anti-CD42-fluorescein isothiocyanate and 5 µL of anti-CD62P-phycoerythrin to 90 µL of citrate-anticoagulated PPP) at a 1:9 volumetric ratio, which were then incubated for 15 min at room temperature away from light. The level of platelet activation was analyzed in a BD FACSCanto II flow cytometer. The solution was then diluted to stain buffer (BSA) at a 1:9 volumetric ratio, and then run on a 3-Laser CytoFLEX Flow Cytometer (Beckman Coulter Life Sciences, Indianapolis, IN, USA). Using the CD42-gated platelets, 10,000 events were counted for each measurement. Platelet activation was assessed by PE median fluorescence intensity associated with presentation of the glycoprotein CD62P. FITC-conjugated goat anti-mouse IgG polyclonal antibodies was used as the non-specific binding control[5].

Confocal microscopy analysis for neutrophil adhesion

The neutrophil rich plasma (NRP) was collected from human whole blood anticoagulated by hirudin (300 IU/mL of blood, HYPHEN BioMed) and sodium citrate synergistically. To test the adhesion of neutrophils on the surface, 200 µL of citrate-anticoagulated NRP was introduced into bare or SPI-coated 96-well-glass bottom-plates (cellvis) and incubated for 30 min under shaking condition (8 mM of $CaCl_2$ and 6 mM of $MgCl_2$ (final concentration) were added in this step). After that, the surfaces were gently washed by stain buffer (BSA) (avoid completely dry) and staining solution was introduced (mixture of 5 µL of allophycocyanin (APC) anti-human CD15 (Biolegend) and 5 µl of FITC anti-human CD11b (Biolegend) to 90 µL Stain Buffer (BSA)) and incubated for 30 min at room temperature away from light. Following incubation, the surface was gently washed with stain buffer (BSA). Then, the buffer was removed and the surface was fixed with 2.5 wt. % glutaraldehyde in PBS solution at 4 °C overnight. The neutrophil adhesion on the surface was measured by Confocal &BX61 Microscope. Images from the same batch with the same brightness and contrast were compared. Each experiment was repeated at least three time.

Neutrophil activation using flow cytometry

The NRP was collected from human whole blood anticoagulated by hirudin (300 IU/mL of blood, HYPHEN BioMed) and sodium citrate synergistically. To test the neutrophil activation after incubating with coating, 200 µL of NRP was introduced into bare or SPI-coated glass vials and incubated for 30 min under shaking condition (8 mM of $CaCl_2$ and 6 mM of $MgCl_2$ (final concentration) were added in this step), TNF-α was used as positive control (Sigma, final concentration: 100 ng/mL). After that, the incubated NRP was added into the corresponding antibody-containing solutions (mixture of 5 µL of APC anti-human CD15 (Biolegend) and 5 µl of FITC anti-human CD11b (Biolegend) to 90 µL Stain Buffer (BSA)) at a 1:9 volumetric ratio, which were then incubated for 30 min at room temperature away from light. The level of neutrophil activation was analyzed in a BD FACSCanto II flow cytometer (3-Laser CytoFLEX Flow Cytometer (Beckman Coulter Life Sciences, Indianapolis, IN, USA)). Using the CD15-gated neutrophil, 10,000 events were counted for each measurement. Neutrophil activation was assessed by FITC median fluorescence intensity associated with presentation of the glycoprotein CD11b. Standard endotoxin (100EU/mL, CSE, Associates of Cape Cod, East Falmouth, USA) was used as the

positive control, and APC-conjugated goat anti-mouse IgG polyclonal antibodies was used as the non-specific binding control[6].

Cell adhesion and cytotoxicity measurements

EA.hy926 cells were purchased from American Type Culture Collection (ATCC, CRL-2922) and used up to a passage number of 50. The cells were cultured using Dulbecco's modified eagle medium (DMEM) containing 10% HI fetal bovine serum (FBS) and 1% penicillin/streptomycin (P/S) in tissue-culture-treated T-75 flasks at 37 °C and 5% CO_2. Upon reaching 70% confluence, cells were dissociated with 0.25% trypsin and 0.05% EDTA (Gibco, 25300062), pelleted by centrifugation at 300 g and resuspended with complete DMEM medium.

The SPI coating was applied on Nunc™ MicroWell™ 96 well-plate under aseptic condition. For cell seeding, cell densities of 1.5×10^4 cells/100 µL were incubated with the coated or uncoated well-plate. The cell proliferation levels at day 1, 3, and 5 were measured based on MTS assay. All steps are performed in accordance with the instructions provided by the manufacturer (Promega corporation). For the cell amount and survival rate at day 5, cells attached on the well-plate were dissociated with 0.25% trypsin and 0.05% EDTA, and then the live/dead cells were stained with Trypan blue, and measured using Celldrop FL (DeNovix). Expression of IL-6 was measured on the coating, with cells treated with TNF-α (sigma, final concentration: 10 ng/mL) serving as the positive control. Medium of cells incubated with culture medium on SPI-coated and blank wells were used to assess IL-6 levels via ELISA method (Thermo Fisher).

Human vascular smooth muscle cells (HVSMC, ATCC, PCS-100-012) were used to evaluate the SMC proliferation on SPI coating. The cells were cultured using smooth muscle cell growth medium (Promo Cell GmbH) in tissue-culture-treated T-75 flasks at 37 °C and 5% CO_2. Upon reaching 70% confluence, cells were dissociated with 0.25% trypsin and 0.05% EDTA (Gibco, 25300062), pelleted by centrifugation at 300 g and resuspended with complete smooth muscle cell growth medium smooth muscle cell growth medium.

The SPI coating was applied on Nunc™ MicroWell™ 96 well-plate under aseptic condition. For cell seeding, cell densities of 1.5×10^4 cells/100 µL were incubated with the coated or uncoated well-plate. The cell proliferation levels at day 1, 3, and 5 were measured based on MTS assay. All steps are performed in accordance with the instructions provided by the manufacturer (Promega corporation). For the cell amount and survival rate at day 5, cells attached on the well-plate were dissociated with 0.25% trypsin and 0.05% EDTA, and then the live/dead cells were stained with Trypan blue, and measured using Celldrop FL (DeNovix). Medium of cells incubated with culture medium on SPI-coated and blank wells were used to assess IL-6 levels via ELISA method (Thermo Fisher). Cells treated with TNF-α (sigma, final concentration: 10 ng/mL) serving as the positive control.

Hemolysis

The hemolysis test was carried out to evaluate the erythrocyte compatibility of the coating. 200 µL of citrate-anticoagulated whole blood was firstly added to bare or SPI-coated glass vials and incubated at 37 °C for 30 minutes, then the incubated blood was collected and diluted by 900 µL of calcium- and magnesium-free PBS solution, and then the red blood cells (RBCs) were isolated from plasma by centrifuging at 2,000 g for 10 minutes. PBS and DI water were used as negative and positive control, respectively. Then the suspensions were centrifuged at 2,000 g for 3 minutes, and the absorbance of the released hemoglobin in the suspensions was measured at 540 nm using a UV-vis spectrometer (UV-1750, Shimadzu Co., Ltd, Japan), and then the hemolysis ratios of the coatings could be calculated by the following formula:

Hemolysis ratio (%) = $100 \times (A_s - A_n)/(A_p - A_n)$

where A_s is the absorbance of the suspensions, A_p and A_n are the absorbance of the positive control and the

negative control, respectively. At least 3 parallel samples were applied to get a reliable value, and the results were expressed as mean ± SD (n = 3)[7].

Blood count assay *in vitro*

Blood count assays were performed to investigate the *in vitro* blood salvage efficacy of the coating. 200 μL of Ethylene Diamine Tetraacetic Acid (EDTA)-anticoagulated whole blood was firstly added to bare or SPI-coated glass vials and incubated at 37 °C for 30 minutes. The whole blood cell differential counts were measured by an automated hematology cell analyzer (BC-5100, Mindray Bio-Medical Electronics Co., Ltd., Shenzhen, China) under the provided instruction manual. Three replicates were averaged and the results were expressed as the mean ± SD (n = 3)[7].

C3a and C5b-9 generation

In general, for the assessment of material-induced complement activation, serum or hirudin-anticoagulated plasma is often used. There is a close link between the coagulation and complement systems and thrombus generation often leads to complement activation. For hirudin anticoagulation, this can also lead to significant complement activation during storage as the blood is not immediately available for complement activation testing after collection. The current anticoagulation strategies all result in a high background of complement activation.

In this study, we developed the ELISA tests based on **synergistic anticoagulation of hirudin and sodium citrate**, in which hirudin inhibits thrombogenesis without inhibiting complement activation and sodium citrate inhibits both complement activation and coagulation activation, but the inhibitory effect of sodium citrate on the coagulation and complement systems is lost after re-calcification and re-magnesium. Therefore, it can be guaranteed that the complement system of the blood is uninhibited only during the incubation with the material. We compared this method with the traditional hirudin anticoagulation method in several experiments and obtained the same trend. We therefore strongly recommend this developed approach for probing the material-induced complement activation.

The levels of C3a and C5b-9 generation were measured by ELISA (Complement C3a Human ELISA Kit, Fisher scientific, USA; Complement C5b-9 Human ELISA Kit, Quidel, San Diego, CA, USA) in human whole blood anticoagulated by hirudin (300 IU/mL of blood, HYPHEN BioMed) and sodium citrate synergistically. 200 μL of whole blood was introduced into PP tubes (background), bare or SPI-coated glass vials. After that, 8 mM of $CaCl_2$ and 6 mM of $MgCl_2$ (final concentration) were added to trigger the complement activation (which is not applied to condition 3), Cobra Venom Factor (CVF) was used as positive control (final concentration of 1 μg/mL, Quidel Corporation). After being incubated for 30 minutes at 37 °C, the whole blood was stopped by the addition of EDTA (final concentration of 10 mM), and centrifuged for 10 minutes at 2,500 g centrifugal force to obtain plasma. Finally, the detections were conducted according to the respective instruction manuals. At least 5 parallel sample groups were applied to get a reliable value, and the results were expressed as mean ± SD[8].

Sheep erythrocyte based hemolytic assay

The sheep erythrocyte based hemolytic Assay was used to determine the level of complement activation (consumption) by the coating with similar anticoagulant protocol as mentioned above. The whole blood anticoagulated by hirudin (300 IU/mL of blood, HYPHEN BioMed) and sodium citrate synergistically was centrifuged for 10 minutes at 2500 g centrifugal force to obtain plasma, which was introduced into bare or SPI-coated glass vials. After that, 8 mM of $CaCl_2$ and 6 mM of $MgCl_2$ (final concentration) were added to trigger the complement activation. The incubation was kept for 1 h under 37 °C. CVF (final concentration of 50 μg/mL, Quidel Corporation) was used as positive control, plasma without replenishment of $CaCl_2$ and $MgCl_2$ was used as

a negative control. After incubation, the plasma samples were diluted with GVB-CM buffer (CompTech, USA) (1:9). After that, 75 μL of the diluted plasma was incubated with antibody-sensitized sheep RBC (75 μL, CompTech, USA) then incubate for 1 h under 37 °C. After incubation, all reactions were stopped by the addition of 10 mM of EDTA. Control tubes containing equal volumes of RBCs and GVB-CM buffer (75 μL) were subjected to blank control, or DI water as 100% lysis control. Intact RBCs were pelleted by centrifugation at 2,000 g for 10 min, and the supernatants were collected. Percentage RBC lysis was calculated using average absorbance values as follows:

$$RBC_{lysis} = (A_{540,\ test\ sample} - A_{540,\ blank}) / (A_{540,\ 100\%\ lysis} - A_{540,\ blank}) \times 100\%$$

Percentage of complement consumption by different samples was expressed as 100%-% RBC_{lysis}[8].

Additional information on animal experiments

The blood count assays and biological parameters level assays of the rabbits before and after experiment were determined by an automated hematology cell analyzer (ADVIA 2120i, SIEMENS AG FWB, Germany) and a biochemical analysis instrument (Cabas C311, Roche, Switzerland), respectively. Before the experiment, the blood was collected from the catheter and stored in the tubes (5 mL, Jiangsu Kangjian Inc., China) containing sodium citrate for automatic blood coagulation analysis, tubes (2 mL, Jiangsu Kangjian Inc., China) containing EDTA for automated hematology cell analysis, and tubes without any anticoagulant for biochemical analysis. After the experiment, for the group of SPI-coated catheter, the blood was collected from the catheter, while for the group of bare catheters, the blood was directly collected from carotid artery since the catheter was totally blocked and we could not collect sufficient blood for testing.

Blood flow simulation using computational fluid dynamics (CFD) mode

Under physiologically relevant conditions inside the catheter, the rabbit blood flow is laminar with a Reynolds number around 81. We treated the rabbit blood in the catheter as a viscous non-Newtonian fluid obeying the Carreau model [5]. The computational domain of the blood flow in the catheter was drawn based on the three-dimensional geometry of the Introcan-W (22G×1″) catheter. The Introcan catheter was halved, and the internal dimensions were measured using a caliper. The mass flow inlet was set to be 3 gram/15 seconds under steady state condition and based on the mass flow rate measured before the treatment. The outlet boundary condition was set to be an outflow open boundary. The simulation was carried out in COMSOL Multiphysics, and an extra fine mesh was generated for the computational domain.

Wash protocol and measurement of surface protein concentration after washing

Citrate-anticoagulated PPP (200 μL) was first incubated with bare or SPI-coated glass vials at 37 °C for 30 min. After incubation, the PPP was gently aspirated, and the surface was gently immersed in PBS solution for 10 second, then the PBS solution was gently aspirated. The proteins adsorbed on the surface after one time wash are defined as loosely bound proteins, the proteins adsorbed on the surface after four time of wash are defined as tightly bound proteins. The proteins adsorbed on the surfaces were washed out by 1 % sodium dodecyl sulfate (SDS) solution, and the protein concentration in the SDS solution was determined with BCA kit according to the manufacturer's instructions, results were compared with those for diluted plasm.

QCM analysis of protein adsorption on SPI coating from plasma

QCM-D was used for measuring the mass and stability of the plasma proteins adsorbed on the SPI or PDA/PEG coated sensor surface. PBS buffer was pumped over the sensor surface for 30 min, and baseline equilibrium was reached. Then the solution was switched to PPP, and the protein adsorption was continued for 30 min. And the

sensor was washed by PBS solution until getting a horizontal line. The mass of the adsorbed protein was analyzed by Q-Sense Dfind.

Cy5 labeling of FXII

The quality of Native Human Factor XII protein (Abcam) and FXII Deficient Human Plasma (Cedarlane IFXIIDP10ML) were tested by aPTT analysis. FXII-deficient plasma (200 μL) was mixed with 10 μL of FXII (8 μg) in PBS/glycerol solution, then aPTT reagent (30 mL) was added followed by incubation for 5 min at 37 °C. The plasma was recalcified and time to clot formation was measured on a blood coagulation analyzer (Satellite Max, Stago).

The fluorescence labeling of FXII was conducted according to the instruction manual of protein Cy5 Labeling Kit (abcam). The labeled protein concentration was measured by NanoDrop (Nanodrop 2000C, Thermo Scientific) with a 1-cm pathlength at 280 nm (A280) and 650 nm (A650). Labelling efficiency is about 2-3 Cy5 molecules per protein.

The function of Cy5-labeled FXII was checked with FXII Deficient Human Plasma by aPTT analysis; results were compared with those for original FXII protein. As FXII may self-hydrolyze during labeling process, this may not be fed through the aPTT test. Therefore, reductant SDS-polyacrylamide gel electrophoresis (PAGE) (12% acrylamide) of Cy5-labeled FXII was applied, results were compared with those for original FXII protein.

***In situ* FXII adsorption detection by flow cytometry**

Glass microspheres (Sigma) with a diameter of 3 um were used as substrates for exploring the *in-situ* adsorption of FXII on the surface. These large sized particles provide a flat surface-like state for protein adsorption and facilitates detection by flow cytometry. The glass microspheres were first cleaned by resuspension-centrifugation circulation, after which SPI coating was applied to the glass microspheres. Modified and unmodified glass microspheres (2.5 mg) and FXII deficient plasma contained Cy5-labeled FXII were co-incubated (10 ug Cy5-labeled FXII /200 μL of FXII deficient plasma) for 30 min under rotation. Subsequently, 20 μL of the solution was transferred to 100 μL of FXII deficient plasma (the purpose was to reduce the background effect from Cy5-labeled FXII in solution) and the Cy5 fluorescence intensity on the surface of the modified and unmodified glass microspheres was detected by 3-Laser CytoFLEX Flow Cytometer (Beckman Coulter Life Sciences, Indianapolis, IN, USA). In order to eliminate background interference from other particles in the plasma, the detection rate was set at 10 μL/min and the detection events over 1,000 per second was recorded, in which case the detection events from the glass microspheres will be significantly higher than those from other particles in the plasma. 500,000 events were counted for each measurement, at least 3 parallel sample groups were applied to get a reliable value.

For evaluating the dynamic exchange of FXII from bulk plasma and surface-plasma interface, modified and unmodified glass microspheres (2.5 mg) and FXII deficient plasma contained Cy5-labeled FXII were co-incubated (10 ug Cy5-labeled FXII /200 μL of FXII deficient plasma) for 30 min under rotation. Subsequently, the microspheres were collected by centrifuge and transferred to 100 uL of normal plasma and incubated for another 30 min. The Cy5 fluorescence intensity on the surface was detected by 3-Laser CytoFLEX Flow Cytometer before and after 30 min incubation.

Surface influence of FXII-SPI coating reciprocal activation

To verify whether the SPI coating affects the interaction between kallikrein and FXII, glass vials coated with SPI coating and uncoated glass vials were incubated with KK/FXII mixture solution (final concentration: 1 μg of KK and 3 μg of FXII in 100 μL of PBS buffer) for 3 min. FXII and KK were co-incubated without surface for 3 min

as the negative control, FXII and KK without incubation as the blank control. After the incubation, 5 μL of the solution was added to 50 μL of S-2302 substrate solution (2 mM in PBS buffer) and pNA generation was detected by UV-Vis at 405 nm in kinetic model, at least 3 parallel sample groups were applied to get a reliable value.

The cleavage efficiency of S-2302 for the surface incubated FXII or PK was also explored. 400 nM of FXII or 400 nM of PK in PBS buffer was incubated with uncoated, HPG-PEG and SPI coated glass vials for 10 min, then 10 μL of the mixture was transferred to 90 μL of S-2302 substrate solution (2 mM in PBS buffer) and pNA generation was detected by UV-Vis at 405 nm in kinetic model, at least 3 parallel sample groups were applied to get a reliable value.

Considering the potential of SPI coating to inhibit contact activation by adsorption of FXIIa[9], the influence of surface-FXIIa interaction on the function of FXIIa was also explored. 50 nM of FXIIa (Enzyme research laboratories) was first incubated with uncoated, HPG-PEG and SPI coated glass vials for 10 min, then the solution was transferred to a new PP tube and PK (400 nM, final concentration) was added and co-incubated for 5 min, 10 μL of the mixture was transferred to 90 μL of S-2302 substrate solution (2 mM in PBS buffer) and pNA generation was detected by UV-VIS at 405 nm in kinetic model, at least 3 parallel sample groups were applied to get a reliable value.

Active dimethyl labelling and conformation analysis of FXII in plasma by mass spectrometry

1 mg of SPI coated or uncoated glass microspheres was incubated with 100 uL FXII solution (10 ug/100 uL of PBS buffer) for 30 min under 37 °C. The FXII solution without surface treatment was applied as blank control. Then 3.2 μl of $NaBH_3CN$ (0.15 M) and 3.2 μl of CH_2O (1%) were added, and the reaction was carried out at 37 °C for 20 min. To stop the reaction, 4 μl of NH_4HCO_3 (2.5 M) was added and incubated for 20 min. Then the reaction was centrifuged and the supernatant was collected. Subsequently, the labeled FXII protein present in the supernatant was subjected to chymotrypsin digestion following the instructions provided with the reagents (https://assets.fishersci.com/TFS-Assets/LSG/manuals/MAN0011638_Mass_SpectroGrade_Endoprotein_UG.pdf). The chymotryptic peptides were dissolved in 0.1% formic acid and 2% acetonitrile (solvent A), directly loaded onto a home-made reversed-phase analytical column. The gradient was comprised of an increase from 9% to 25% solvent B (0.1% formic acid in 90% acetonitrile) over 36 min, 25% to 35% in 18 min, 35% to 80% in 3 min holding at 80% for the last 3 min, all at a constant flow rate of 500 nL/min on an EASY-nLC 1200 UPLC system.

The peptides were subjected to NSI source followed by tandem mass spectrometry (MS/MS) in Q Exactive™ HF-X (Thermo) coupled online to the UPLC. The electrospray voltage applied was 2.1 kV. The m/z scan range was 490 to 700 for full scan, and intact peptides were detected in the Orbitrap at a resolution of 120000. A data independent analyzing (DIA) procedure was applied for data collection. Peptides were then selected for MS/MS using NCE setting as 28 and the fragments were detected in the Orbitrap at a resolution of 15,000. A data-independent procedure that alternated between one MS scan followed by 20 MS/MS scans. Automatic gain control (AGC) was set at 3E6 for full MS and 1E5 for MS/MS. The maximum IT was set at 220 ms for full MS and auto for MS/MS. The isolation window for MS/MS was set at 1.4 m/z.

The resulting MS data were processed using Skyline (v.21.1). Peptide settings: enzyme was set as Chymotrypsin [KR/P], Max missed cleavage set as 4. The peptide length was set as 7-25. Variable modification was set as dimethylation on Lys and fixed modification was set as alkylation Cys. Transition settings: precursor charges were set as 2, 3, ion charges were set as 1, ion types were set as b, y. The product ions were set as from ion 3 to last ion, the ion match tolerance was set as 0.02 Da.

The calculation formula of labeling efficiency is as follows: labeling efficiency=labeled peptide intensity/ (unlabeled peptide intensity + labeled peptide intensity)

Supplementary Results and Extended Discussion:

Supplementary results for coating characterization:

The average molecular weight of the SPCM used here is 11,000 g/mol with a polydispersity (Mw/Mn) of 1.4, and has a zeta potential of approximately + 5 mV in aqueous solution. For generating the SPI coating, initially, dopamine/PEG co-deposition was achieved using sodium periodate as an oxidant [4, 10] to generate a thin coating. Then the final SPI coating was synthesized by reacting SPCM with the PDA/PEG coating (**Fig. 1d**) where the quinone groups of PDA were covalently bonded with primary amine groups of SPCM via a Michael-type addition/Schiff base reaction. The stability of the SPI coating was confirmed by the absence of thickness change and spectral features by exposing the coating to harsh conditions such as long-time incubation with 3M NaCl solution (for destabilizing electrostatic interactions), 0.3 M urea (for breaking hydrogen bonding interactions) and 1 wt% NaN_3 solution (**Supplementary Fig. 5, 7-8)**. The surface morphology of the SPI coating, assessed by scanning electron microscopy (**Supplementary Fig. 9)** and by atomic force microscope (AFM) (**Supplementary Fig. 10**) revealed a grainy uniform surface feature. The coating did not exhibit hydrogel-like properties, as evidenced by the lack of significant surface morphology and hydrophilicity changes in aqueous conditions assessed by AFM (**Supplementary Fig. 10**). The water contact angle of the SPI coating (~10°) confirms the highly hydrophilic nature of the coating irrespective of the underlying substrate (**Supplementary Fig. 11a**). The surface charge as measured by the surface zeta potential (**Supplementary Fig. 11b**) showed that the SPI coating has a slight negative charge (-7 mV). Conjugation of positively charged SPCM did not completely reverse the surface charge of PDA/PEG (~ -35 mV) to positive, possibly due to the low cationic charge of SPCM. However, the surface charge of the SPI coating was decreased after its interaction with heparin to ~-60 mV confirming that strong negatively charged molecules can bind to the SPI coating. The data also suggested that the SPI coating was able to maintain the desired interaction while avoiding excess exposure to surface positive charges unlike naked polycationic coating (e.g., PEI coating which has a surface zeta potential of +19 mV).

Supplementary results on the hemocompatibility of SPI coating:

We also assessed the interaction of the SPI coating with circulating blood cells that might impact on the biocompatibility of a blood-contacting material/device. Compared to surfaces such as glass, PDA/PEG, and naked positively charged surfaces, the SPI coating significantly resisted the adhesion and activation of platelets and neutrophils as measured by laser scanning confocal microscopy (**supplementary fig. 19-20** (platelets), **23-24** (neutrophils)) and flow cytometry (**supplementary fig. 22** (platelets) and **supplementary fig. 25** (neutrophils)). Since platelet adhesion is significantly modulated by shear stress [11, 12], that is reduced on the SPI coating under both simulated arterial and venous conditions (**supplementary fig. 21)**. The SPI coating also did not induce hemolysis or affect whole blood cell count (**supplementary fig. 27**). The SPI coating did not affect complement activation as measured by the accumulation of C3a (initiation of complement) or sC5b-9 (terminal pathway of complement) in hirudin plasma incubated with the SPI coating [13]. Nor did it affect total complement activation via the classical pathway, as measured with a hemolytic assay using antibody-sensitized sheep erythrocytes (**supplementary fig. 28-29**) [8]. The SPI coating was also non-toxic to endothelial cells (Ea. Hy926) and smooth muscle cells (HAoSMCs), while also being unfavorable for the adhesion of these two cell types (**supplementary fig. 26**).

Supplementary results on coating-FXII interaction:

Our initial studies using QCM measurements showed that considerable amounts of proteins were adsorbed from human plasma onto the SPI coating in comparison to the control surface (**supplementary fig. 32**). We analyzed

the adsorbed protein corona on the SPI coating from plasma to understand the role of bio-interface adsorbed proteins and their antithrombotic function. The adsorbed proteins can be loosely or tightly bound to the surface, and their composition and surface organization can potentially affect the functionality of the material surface. Results showed that the amounts of adsorbed proteins on bare glass and SPI coated glass were not significantly different after washing steps (**supplementary fig. 33** and **34a**). We then looked at the composition of loosely and tightly bound proteins on glass and SPI coated surfaces using proteomic techniques and found the SPI coated surface had more adsorbed FXII than the glass surface.
Since proteomics studies do not fully reflect the interaction between FXII and the SPI coating as a washing step that is mandatory prior to these analyses. This may result in the loss of some loosely bound proteins on the surface [14]. Therefore, *in situ* detection of FXII can be more powerful to understand its surface interaction. Thus, a flow cytometry-based technique was developed to detect the interaction of specific proteins on the surface in plasma *in situ* without the washing process (**Fig. 5d**). For this purpose, FXII was first labelled with Cy-5 fluorescence (Cy5-FXII) without losing its function (**supplementary fig. 36**) or hydrolysis (**supplementary fig. 37**). The microparticle samples were incubated with FXII-deficient plasma containing labeled FXII. The fluorescence intensity of the protein corona on the particles can reflect the concentration of the adsorbed FXII. The results showed that both glass surface and SPI coated surface exhibited an increased fluorescence intensity, compared to that in FXII deficient plasma suggesting that FXII was binding to both surfaces. However, the fluorescence intensity of the SPI coated surface was significantly higher than that on the glass surface.

Supplementary results on mechanism of modulation of the contact activation:

In the contact system, surfaces may promote the generation of FXIIa with the assistance of PK. FXIIa can further cleave PK to form kallikrein (KK), whereupon KK can then back activate FXII to generate more FXIIa, resulting in a positive feedback loop[15, 16]. FXIIa can also cleave and activate factor XI (FXI) to generate FXIa and thus trigger activation of factor IX (FIX), leading to downstream generation of factor Xa (FXa) and thrombin, feeding into the coagulation cascade. Since contact activation in plasma is regulated by various proteins, an *in vitro* simulation of FXII-PK reciprocal activation is measured in a simplified system. FXII-PK reciprocal activation is recognized as the initiation step of contact activation [17].

After incubation of an FXII-PK mixture in PBS with different surfaces, cleavage of the substrate S-2302 was quantified. The SPI coating induced the lowest level of substrate cleavage (max slop ~1.1×10^{-4}/s for 3 min incubation and ~4.1×10^{-4}/s for 5 min incubation), while the HPG-PEG coating (max slop~1.1×10^{-3}/s for 3 min incubation and ~1.7×10^{-3}/s for 5 min incubation) and glass surface (max slop ~9.4×10^{-3}/s for 3 min incubation and ~1.4×10^{-2}/s for 5 min incubation) showed relatively high S-2302 cleavage (**Fig. 6a**). The extent of S-2302 cleavage by the different surfaces corresponded with their propensity to induce thrombosis. The catalytic activity of glass and HPG-PEG coating was at least 30 and 4 times, respectively, higher than that of the SPI coating. This was further confirmed by SDS-PAGE analyses that showed that the SPI coated surface generated lower amounts of FXIIa than the HPG-PEG coating or the glass surface (**supplementary fig. 38**).

Next, we incubated different surfaces with FXII or PK separately to observe direct activation of these proteins. Our results showed that none of the evaluated surfaces-initiated protein cleavage and did not exhibit catalytic activity (**supplementary fig. 39a-b**). Furthermore, we found that FXII incubated with the glass surface could significantly enhance the reciprocal activation with PK even after the surface was removed from the solution (**Fig. 6c** and **supplementary fig. 41**). This finding is notable, as it suggests that material-induced clotting activation can be initiated far away from the surface and the desorbed activated proteins can act as initiating points in solution. Unlike the glass surface, such activity was not seen in the case of the SPI coating. Moreover, PK alone could not significantly enhance reciprocal cleavage with FXII after incubation with the surface in solution (**supplementary fig. 39c**). To further probe whether the SPI coating can influence the activity of FXIIa, we investigated the effect

of different surfaces (glass, HPG-PEG coating and SPI coating) on the activity of FXIIa. As shown in **Fig. 6d,** the SPI coating did not inhibit the function of FXIIa suggesting that the inhibition of contact activation by the SPI coating was not due to the adsorption of FXIIa onto the surface. Further, following incubation with the SPI coating, KK retained its original enzymatic cleavage efficiency towards FXII, suggesting that the SPI coating is not achieving its ability to prevent contact activation by limiting KK activity or protecting FXII cleavage sites (**supplementary fig. 40**).

We also studied the dissociation of FXII after its interaction with different surfaces using flow cytometry. For this, FXII-deficient plasma containing Cy5-FXII was initially incubated with glass, PDA/HPG-PEG coating and SPI coated surface, and the plasma was removed. Then these surfaces were incubated with normal plasma without labeled FXII, and a time-dependent fluorescent intensity profile of the surface was measured. As shown in **supplementary fig. 42**, all three surfaces showed an increase in fluorescence intensity compared to that of surfaces incubated with FXII deficient plasma without Cy5-labeled FXII suggesting that FXII is binding to the surface. However, subsequent incubation with normal plasma decreased the fluorescent intensity of the surface suggesting that the adsorbed Cy5-FXII was exchanged with other proteins and released the adsorbed FXII.

To investigate whether the surface-dissociated FXII undergoes conformational changes, a modified targeted proteomics approach and dimethyl labeling techniques were applied. We evaluated the conformational changes of FXII upon interaction with different surfaces; changes in the labeling efficiency of different lysine residues were correlated as conformational change as described[18, 19]. Since the dimethyl-labeling efficiency and enzymatic efficiency of FXII are significantly altered after the conformational change, the change in intensity of dimethyl-labeled lysine on FXII qualitatively reflects the extent of altered conformation (as shown in **Fig. 6e**). For those segments of FXII interacting with the glass surface, the labeling efficiency of K32 decreased from 67.42% to 56.55%, indicating significant structural changes induced by the glass to the surface-dissociated FXII, which explains the enhanced activity of FXII as shown in **Fig. 6c.** However, in the case of the SPI coating, the labeling efficiencies of K32 (from 67.42% to 83.22%), K146 (from 95.76% to 48.62%), and K595 (from 19.89% to 74.5%) all showed significant changes, suggesting that the surface-disassociated-FXII was undergoing significant structural changes upon its interaction with the SPI coating in different segments of the protein in comparison to glass surface. However, these structural changes did not result in enhanced activity of FXII.

Supplementary extended outlook:

Under normal physiological conditions, the intrinsic coagulation pathway initiated via the contact activation system plays a major role in surface-induced thrombosis. The initial protein that triggers such activation is FXII which can adsorb to polyanionic molecules such as polyphosphate, extracellular DNA and RNA, and other foreign surfaces to generate FXIIa with the assistance of PK. FXIIa can further cleave PK to form kallikrein, whereupon KK can then back activate FXII to generate more FXIIa, resulting in a positive feedback loop [15, 16]. FXIIa can also cleave and activate FXI to generate FXIa and thus trigger activation of FIX, leading to downstream generation of FXa and thrombin, feeding into the coagulation cascade. In addition, kallikrein can bind to high molecular weight kininogen (HMWK), releasing bradykinin which promotes vasodilation, vascular permeability, and the recruitment of neutrophils - an important connection between coagulation and inflammation [20]. Most of the currently used biomaterials activate the contact pathway, leading to both thrombus generation and inflammation, even in the presence of negative regulatory proteins such as C1INH. Thus, material surfaces that can prevent the activation of FXII are the key to providing antithrombotic function and will have immense value in creating safe and functional medical devices for diverse blood contact applications.

The FXII-surface interaction can result in a conformational change of FXII which may either loosely bind to the surface or can be desorbed back to the plasma, resulting in increased FXII-PK reciprocal activation away from the

surface, resulting in coagulation initiation. Thus, inhibiting protein interactions on a surface may not necessarily protect against the induction of thrombus generation. Current strategies used in the development of antithrombotic surfaces that are based on antifouling surfaces (the most commonly used approach) need critical reconsideration. While antifouling surfaces can reduce tightly bound proteins, they cannot completely prevent surface-protein interactions and the initiation of the clotting process. Although many protein-repelling surfaces have been reported to exhibit antithrombotic functionality, there has been a lack of separate analysis on their impact on the contact activation system in plasma that is incubated with the surface[21, 22]. Considering the diverse range of protein-repelling surfaces reported to date, it is unlikely that all of them have similar effects on the contact activation system. Even though protein-repelling surfaces effectively mitigate many blood incompatibilities, a separate analysis of their effects on the contact activation system is warranted.

An important finding from this work is that the SPI coating exhibits a strong interaction with FXII and induces a conformational change to FXII; however, these events do not lead to increased FXII-PK reciprocal activation resulting in reduced or significantly dampened surface-induced coagulation initiation. The structure of the coating and the presentation of the cationic charges on the surface could be responsible for this finding. Prior to this report, FXII/FXIIa inhibitors have been grafted onto material surfaces and effectively inhibited thrombus generation induced by the materials[23]. These surface-immobilized inhibitors are believed to exert inhibitory effects on FXII/FXIIa similar to those in plasma. These studies, while not considering issues such as non-specific protein adsorption, inhibitor denaturation and detachment, universality and cost, and cannot strictly be categorized as an antithrombotic surface, nonetheless reflect the concept of controlling the interaction of coagulation factors on material surfaces.

Supplementary figures:

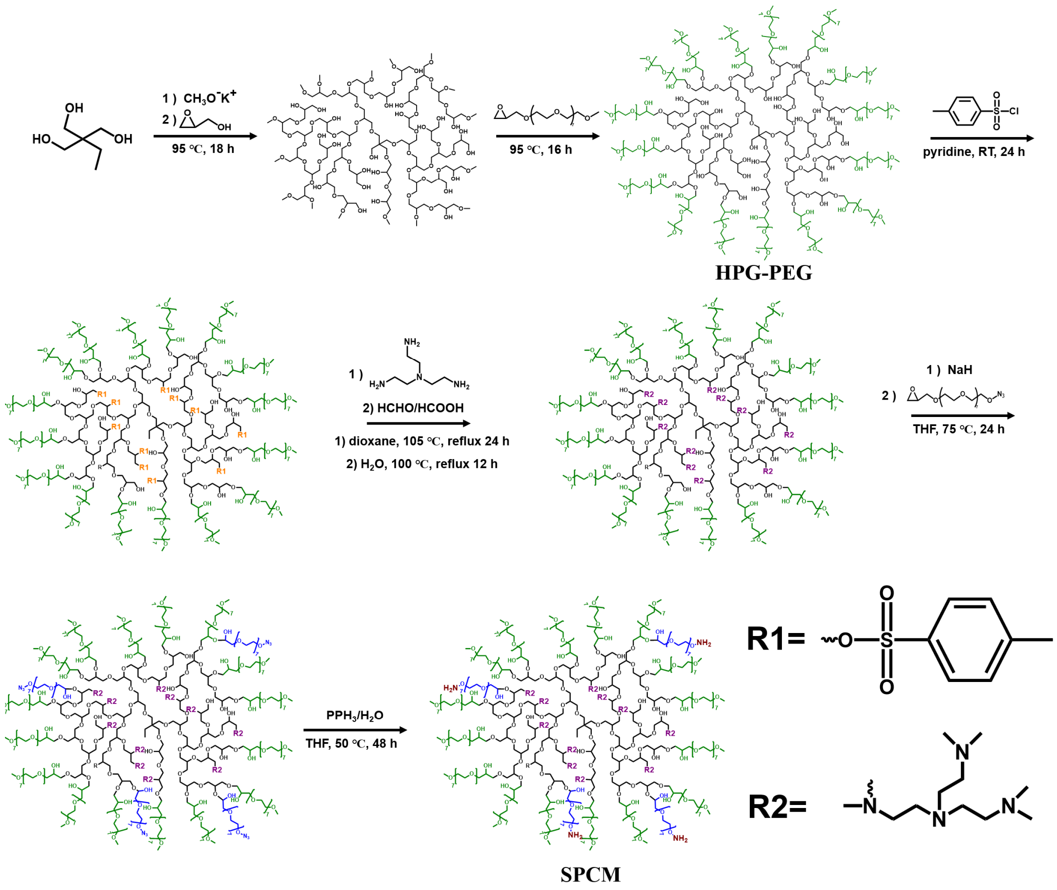

Supplementary figure 1: Scheme for the synthesis of the SPCM.

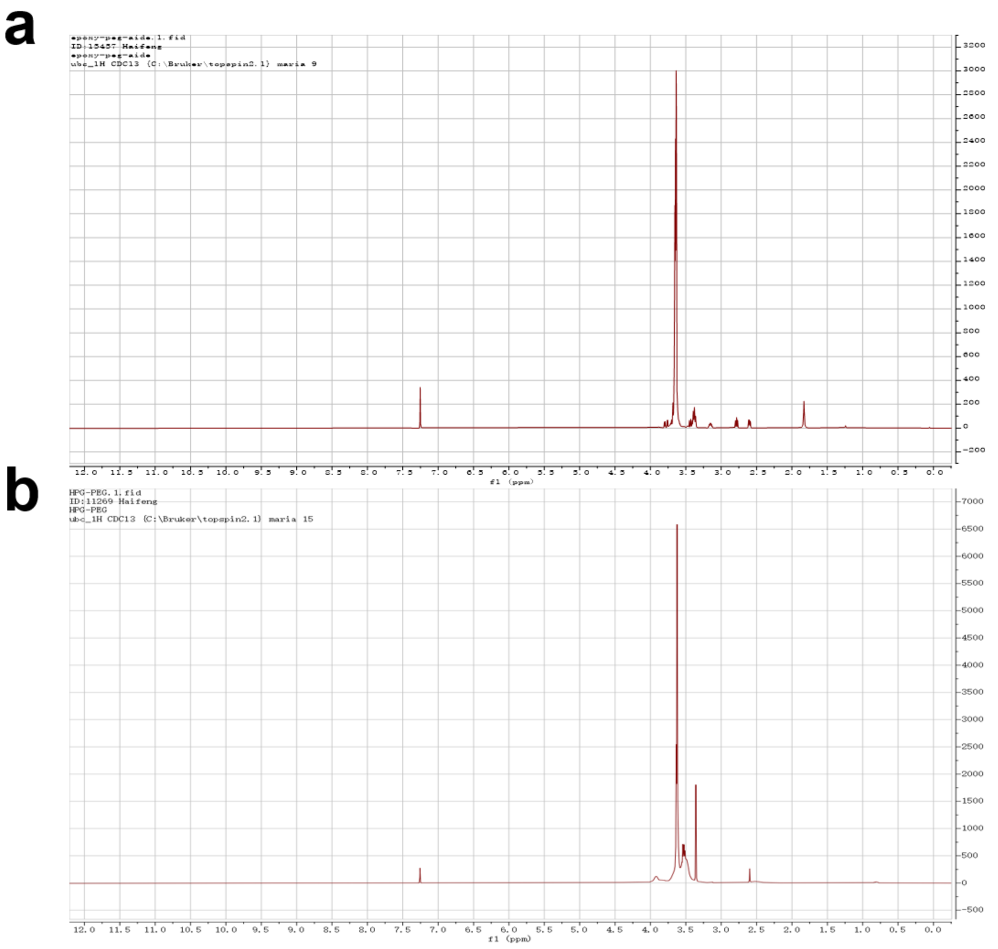

Supplementary figure 2: ^{1}H NMR spectrum ($CDCl_3$, 300 MHz) of epoxy-PEG_{400}-azide (a) and HPG-PEG (b).

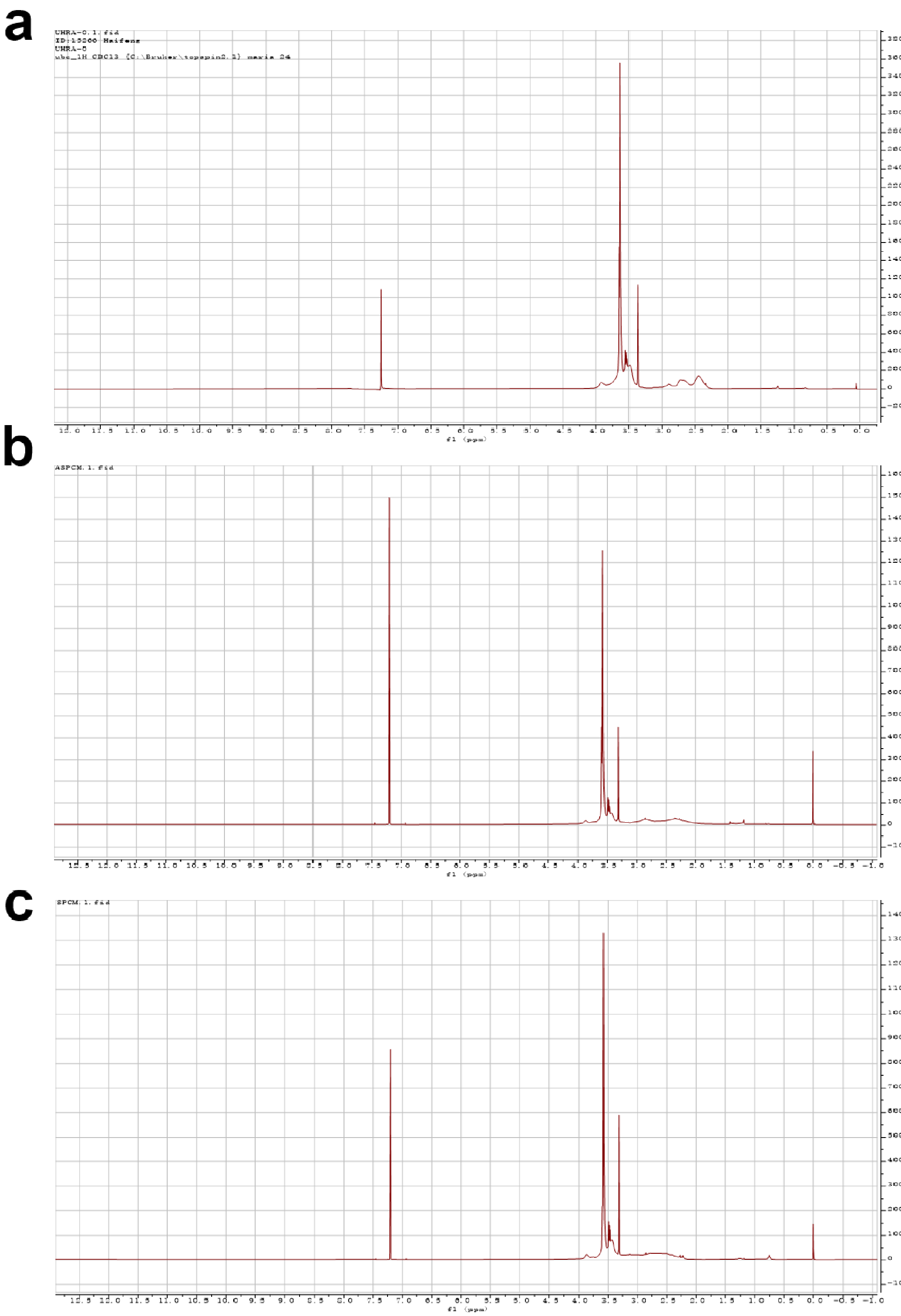

Supplementary figure 3: ^{1}H NMR spectrum ($CDCl_3$, 300 MHz) of UHRA-10 (a), A-UHRA-10 (b), and SPCM (c).

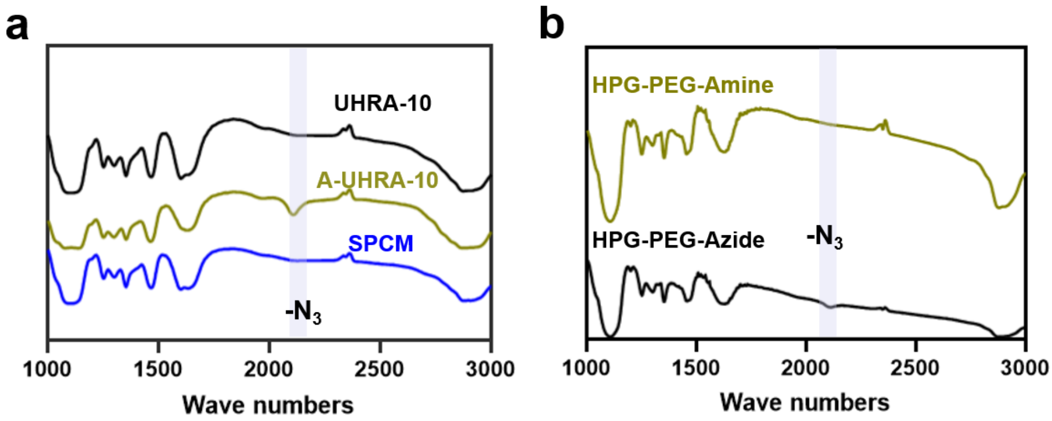

Supplementary figure 4: (a) FTIR spectra UHRA-10, A-UHRA-10, and SPCM. In comparison to the SPCM (without azido modification), the A-UHRA-10 showed a clear peak at 2100 cm^{-1}, representing the successful conjugation of azide containing PEG to the molecule. After Staudinger reduction, the peak at 2100 cm^{-1} for final SPCM disappears, representing the successful conversion of azide to primary amine. (b) FTIR spectra for azido group modified HPG-PEG and primary amine modified HPG-PEG.

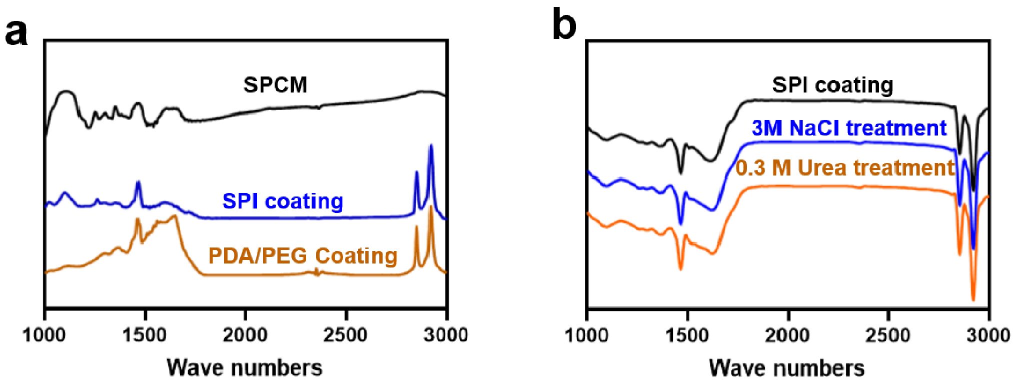

Supplementary figure 5: FTIR spectra for (a) SPCM, SPI coating and PDA/PEG coating (absorbance mode); and (b) SPI coating after 3 M NaCl or 0.3 M urea solution treatment (transmittance model). A wide vibrational peak corresponding to C-N or C-O at 1020-1200 cm^{-1} can be observed for SPCM, this signal is also present in the SPI coating but not in the PDA/PEG coating and represents the successful preparation of the SPI coating. After 3 M NaCl or 0.3 M urea solution treatment, The FTIR spectrum shows no significant change, indicating that electrostatic or hydrogen bonding interactions do not contribute to the SPI coating, and the SPCM is covalently grafted onto the PDA surface[24].

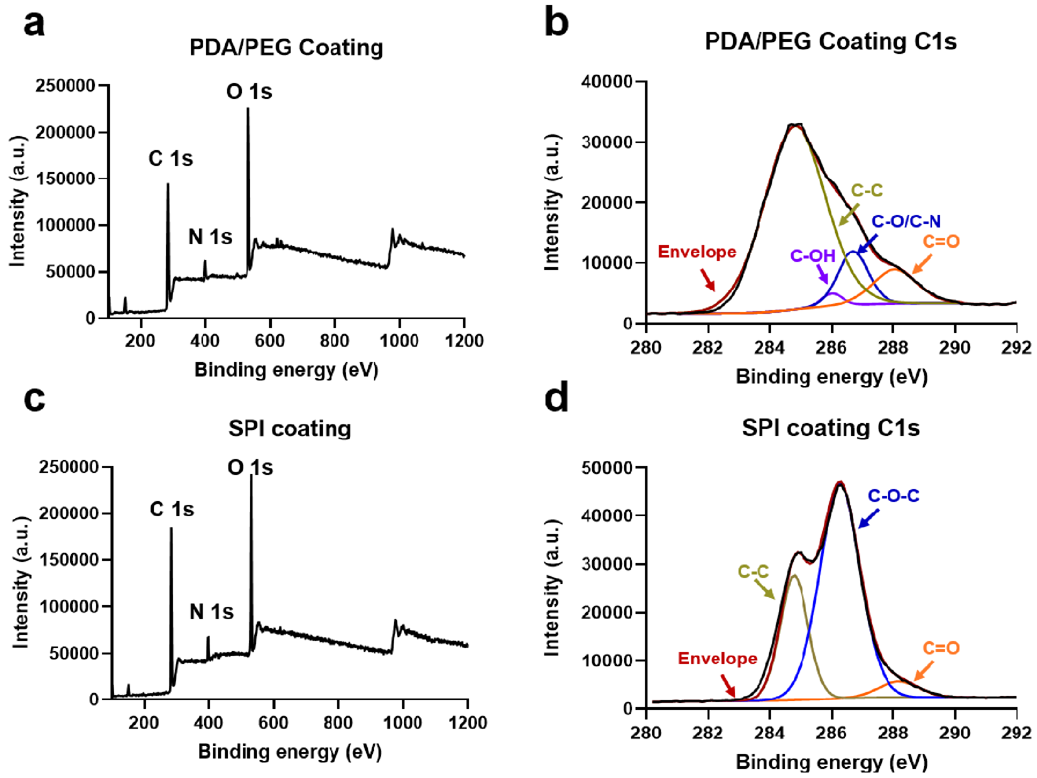

Supplementary figure 6: XPS spectra for PDA/PEG and SPI coating. (a) XPS survey spectra for PDA/PEG coating, (b) high resolution C1s spectra for PDA/PEG coating, (c) XPS survey spectra for SPI coating, and (d) high resolution C1s spectra for SPI coating. The peak intensity of 286.5 eV from C-O-C is increased for the SPI coating compared to the PDA/PEG coating, this can be attributed to the ether bonds from the SPCM.

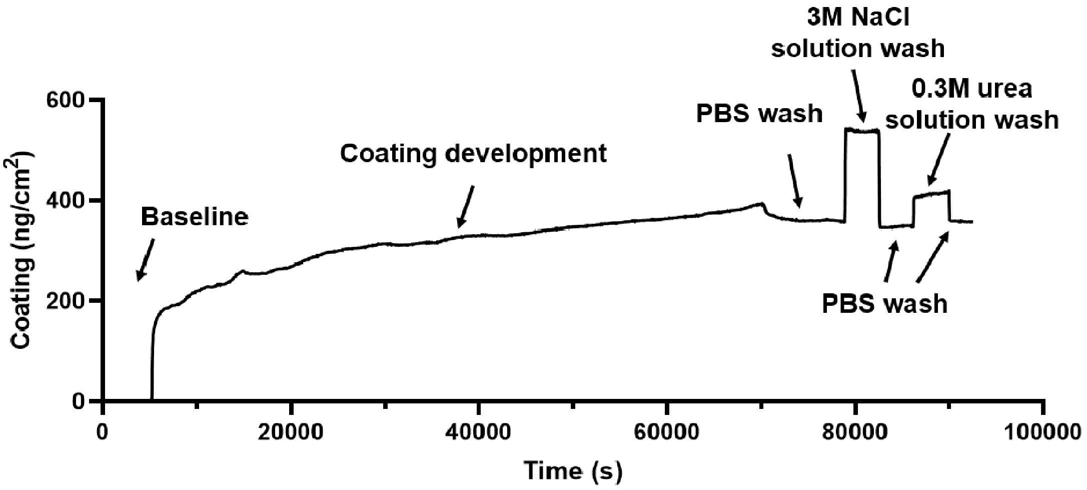

Supplementary figure 7: The formation and stability of SPI coating. QCM spectrum for SPI coating development and stability after 3 M NaCl or 0.3 M urea solution treatment. After obtaining a reliable baseline, it was clearly observed that the mass of the SPCM increased significantly on the PDA/PEG coated sensor, which maintained to ~300 ng/cm^2 after PBS washing. After treatment with 3 M NaCl or 0.3 M urea solution, the mass of the SPI coating maintained to the original value. Harsh experimental conditions are used to evaluate the stability of the coating.

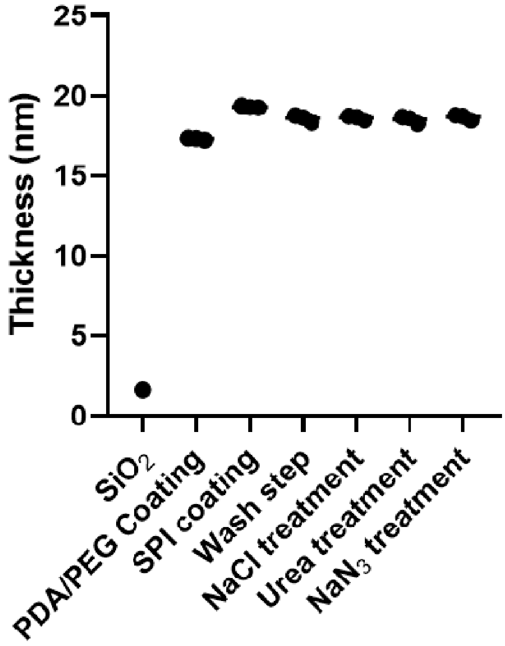

Supplementary figure 8: Thickness change of the Si wafer surface after different treatment processes as measured by ellipsometer. After each treatment, three randomly selected locations on the wafer surface were evaluated, which are not apparent in the data points due to very small difference between the data points. Error is within the size of the symbol. A significant increase in the thickness was observed after the PDA/PEG coating on silicon wafer, which increases further after the SPI coating. The treatment of 3 M NaCl, 0.3 M urea, and 1 wt% NaN_3 solution did not reduce the thickness of the coating. The data indicated that the coating has good stability.

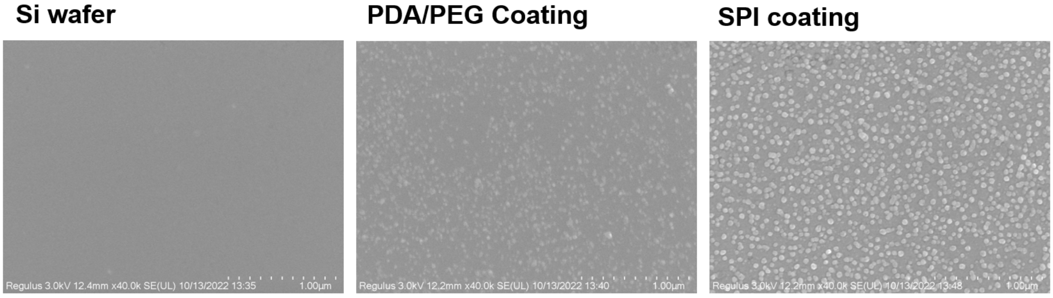

Supplementary figure 9: SEM micrographs of Si wafer surface before and after the application of PDA/PEG and SPI coating. The initial wafer surface was smooth and after PDA/PEG coating the PDA nanoparticles are uniformly distributed on the surface[25] and the SPI coating process does not significantly alter the morphology of these nanoparticles. At least 2 times each experiment was repeated independently with similar results.

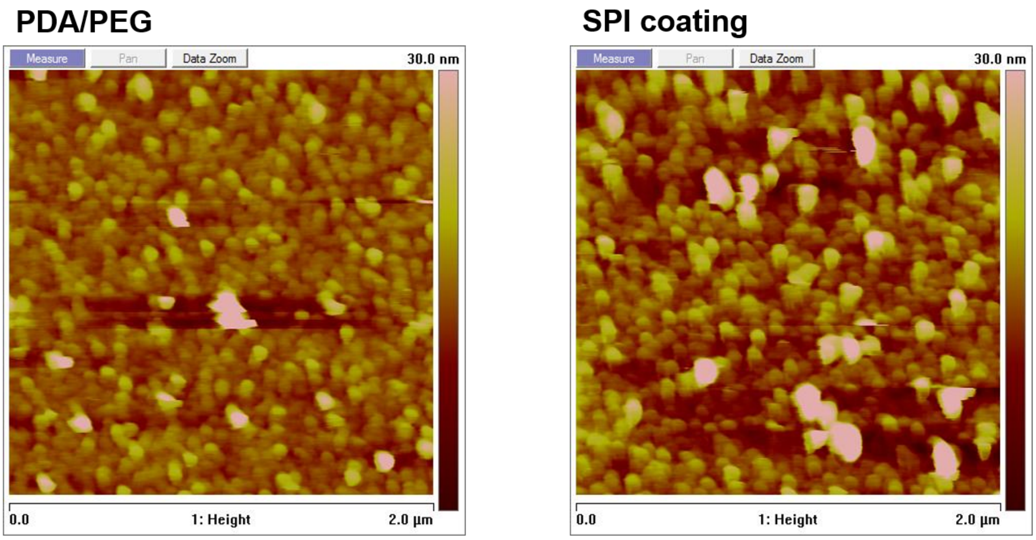

Supplementary figure 10: AFM image for Si wafer surface after PDA/PEG and SPI coating process. The analysis was conducted in aqueous environment to explore the morphology change of the SPI coating modified nanoparticles. The results indicate that SPI coating modified PDA nanoparticles show no significant swelling behavior. At least 2 times each experiment was repeated independently with similar results.

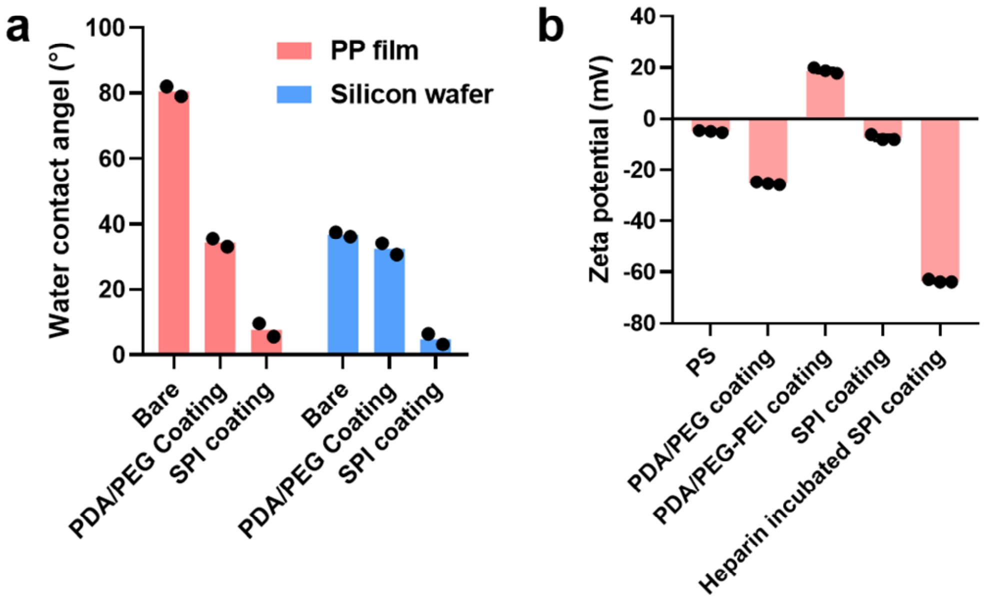

Supplementary figure 11: Hydrophilicity/hydrophobicity and surface charge. (a) Water contact angles (WCA) of for PP film and Silicon wafer after PDA/PEG and SPI coating process (n = 2 biologically independent samples). (b) Zeta potential of polystyrene spheres (~7 um) after different coating process (pH~7) (n = 3 technically replicate samples). All values are expressed as the mean.

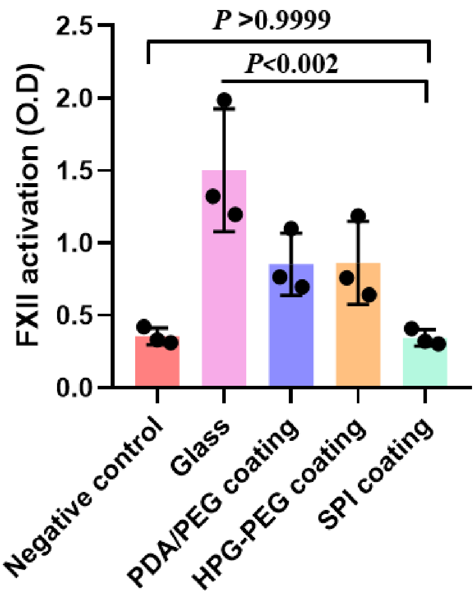

Supplementary figure 12: S-2302 cleavage efficiency for plasma incubated with glass vials treated by different coatings. Changes in OD 405 nm were measured on a plate reader after 30 min incubation. The SPI coating can inhibit the pNA generation while other coating could not (n = 3 biologically independent samples). Multiple comparisons were performed using one-way ANOVA. If significance was determined, post-hoc multiple comparison analysis was conducted with Tukey test. All values are expressed as the mean ± s.d. Exact p-values are provided in the corresponding figures, statistical significance was defined as $p < 0.05$.

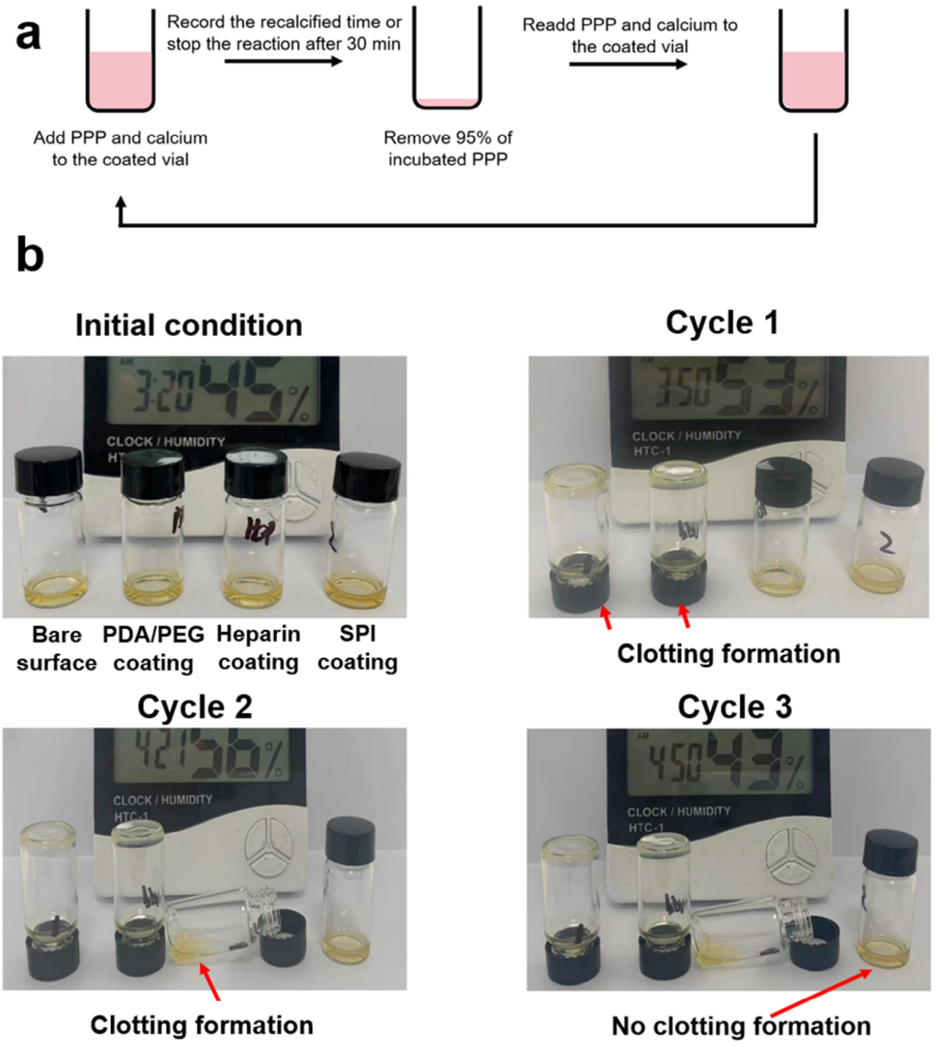

Supplementary figure 13: Recalcification clotting time of plasma incubated with glass vials after different coating treatments. (a) Schematic diagram of the test procedures and (b) photographs of the test results. The SPI coating can significantly prolong the recalcification clotting time. Unlike the heparin coating, there is no functional saturation of the SPI coating as it is the proteins adsorbed on the SPI coating that determine the function, whereas the function of the heparin coating is considered to result from the binding of ATIII/thrombin or heparin cofactor/thrombin on the surface [26].

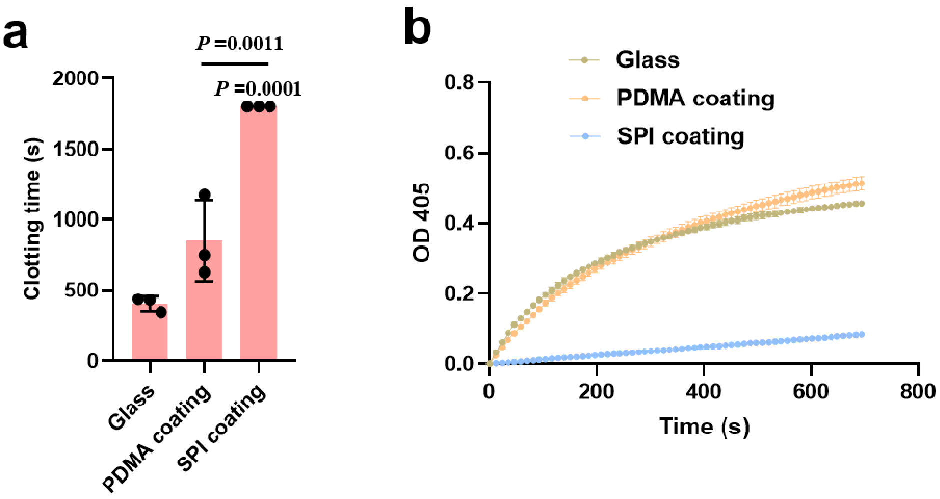

Supplementary figure 14: Antithrombotic property for different coatings. (a) Recalcification time of plasma after incubation with different coating (n = 3 biologically independent samples). Multiple comparisons were performed using one-way ANOVA. If significance was determined, post-hoc multiple comparison analysis was conducted with Tukey test. (b) Cleavage efficiency of S-2302 by an in vitro simulated contact initiation system (3 μg/100 μL of FXII and PK in PBS buffer) after incubation with different coating (n = 3 biologically independent samples). All values are expressed as the mean ± s.d. Exact p-values are provided in the corresponding figures, statistical significance was defined as $p < 0.05$.

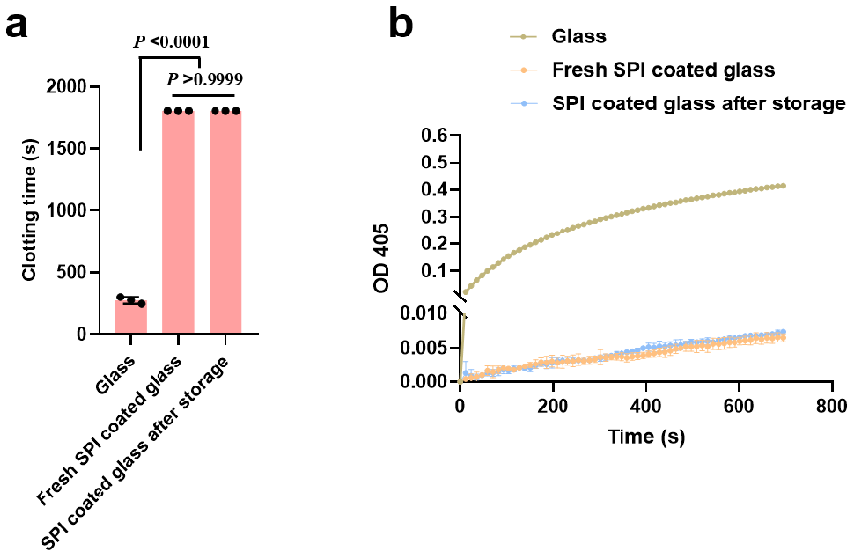

Supplementary figure 15: Long-term stability of the coating. (a) Recalcification time of plasma after incubation with SPI coating after different storage condition (n = 3 biologically independent samples). Multiple comparisons were performed using one-way ANOVA. If significance was determined, post-hoc multiple comparison analysis was conducted with Tukey test. (b) Cleavage efficiency of S-2302 by an in-vitro simulated contact initiation system (3 μg/100 μL of FXII and PK in PBS buffer) after incubation with SPI coating after different storage condition (n = 3 biologically independent samples). All values are expressed as the mean ± s.d. Exact p-values are provided in the corresponding figures, statistical significance was defined as $p < 0.05$.

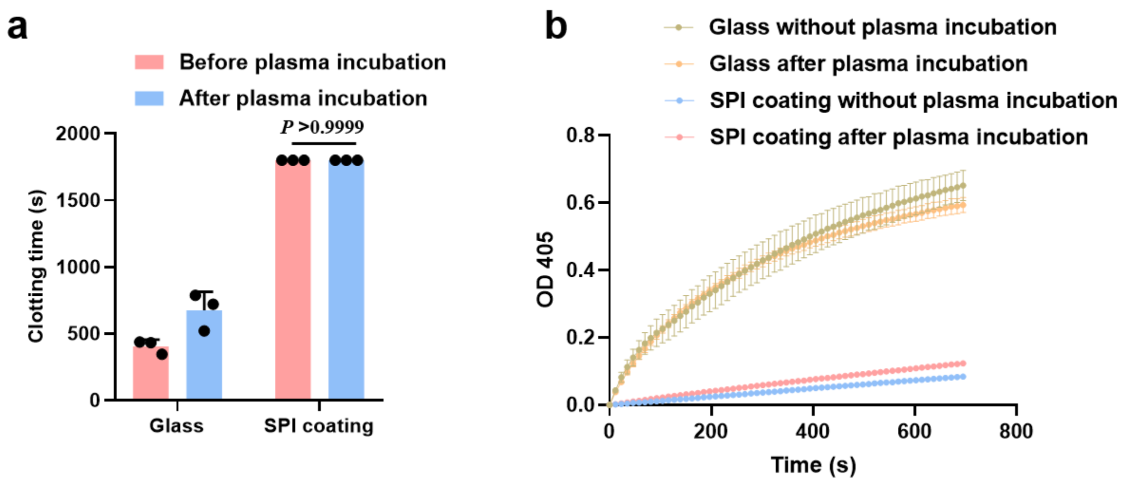

Supplementary figure 16: Functional stability of the coating after incubation with plasma. (a) Recalcification time of plasma after incubation with SPI coating with or without plasma protein fouling (n = 3 biologically independent samples). Unpaired, two-tailed student's t-test was applied for comparison of SPI coating with or without plasma protein fouling. (b) Cleavage efficiency of S-2302 by an in-vitro simulated contact initiation system (3 µg/100 µL of FXII and PK in PBS buffer) after incubation with SPI coating with or without plasma protein fouling (n = 3 biologically independent samples). All values are expressed as the mean ± s.d. Exact p-values are provided in the corresponding figures, statistical significance was defined as $p < 0.05$.

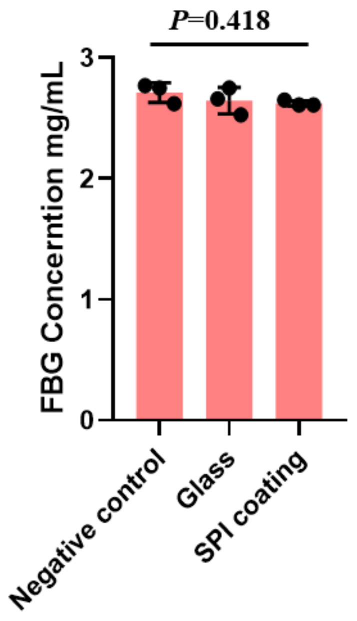

Supplementary figure 17: Fibrinogen concentration of the PPP incubated with glass vials before and after SPI coating treatment. The coatings did not significantly adsorb fibrinogen from the plasma (n = 3 biologically independent samples). Multiple comparisons were performed using one-way ANOVA. If significance was determined, post-hoc multiple comparison analysis was conducted with Tukey test. All values are expressed as the mean ± s.d. Exact p-values are provided in the corresponding figures, statistical significance was defined as $p < 0.05$.

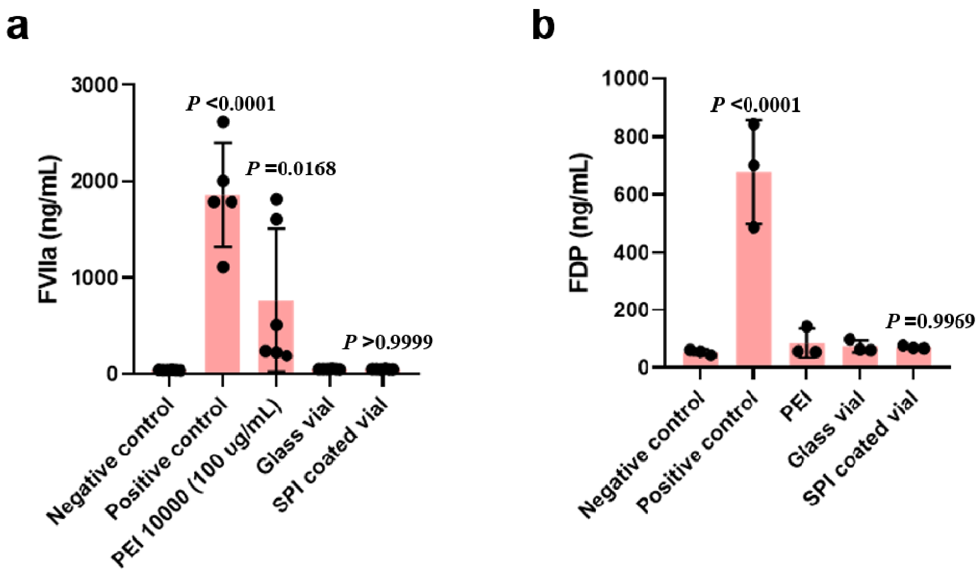

Supplementary figure 18: Detection of SPI coating impact on other potential coagulation pathway activation. (a) FVIIa activation of plasma treated with different samples (n = 5 biologically independent samples). Multiple comparisons were performed using one-way ANOVA. If significance was attained, post-hoc multiple comparison analysis was conducted with Tukey test. (b) Measurement of fibrinogen degradation products (FDP) levels in plasma treated with different samples (n=3 biologically independent samples), reflecting whether SPI coating induces abnormal activation of the fibrinolytic system. Multiple comparisons were performed using one-way ANOVA. If significance was attained, post-hoc multiple comparison analysis was conducted with Tukey test. All values are expressed as the mean ± s.d. Exact p-values are provided in the corresponding figures, statistical significance was defined as $p < 0.05$.

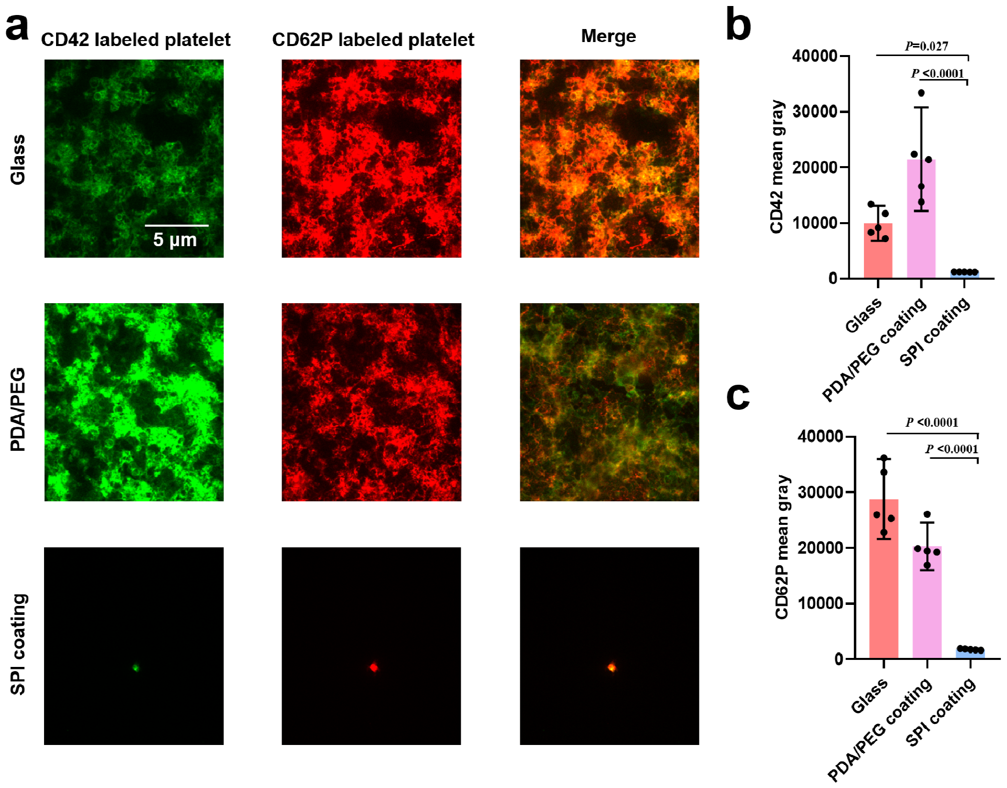

Supplementary figure 19: Platelet adhesion on coatings from platelet rich plasma. (a) Platelet adhesion and activation on bare, PDA/PEG or SPI coated glass surface observed by confocal microscope. Green represents CD42-labelled platelets (normal state) and red represents CD62P-labelled platelets (active state), (b) CD42 mean gray and (c) CD62P mean gray on glass surface before or after PDA/PEG or SPI coating treatment. Five random images for each group with same brightness and contrast were selected for statistically measuring fluorescence intensity. Multiple comparisons were performed using one-way ANOVA. If significance was determined, post-hoc multiple comparison analysis was conducted with Tukey test. All values are expressed as the mean ± s.d. Exact p-values are provided in the corresponding figures, statistical significance was defined as $p < 0.05$.

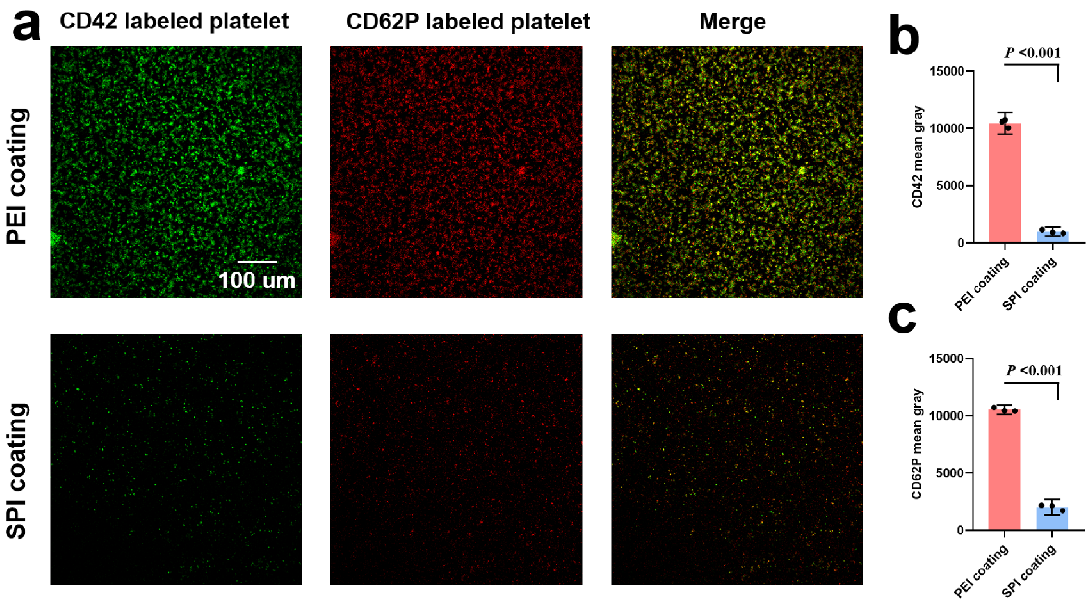

Supplementary figure 20: Platelet adhesion from human platelet rich plasma. (a) Platelet adhesion and activation on glass surface after PEI or SPI coating treatment observed by confocal microscope. Green represents CD42-labelled platelets (normal state) and red represents CD62P-labelled platelets (active state), (b) CD42 mean gray and (c) CD62P mean gray on glass surface before or after PDA/PEG or SPI coating treatment. Three random images for each group with same brightness and contrast were selected for statistically measuring fluorescence intensity. Unpaired, two-tailed student's t-test was applied. Positively charged surfaces tend to adhere platelets, while SPI coating inhibits the interaction between platelets and the surface. All values are expressed as the mean ± s.d. Exact p-values are provided in the corresponding figures, statistical significance was defined as $p < 0.05$.

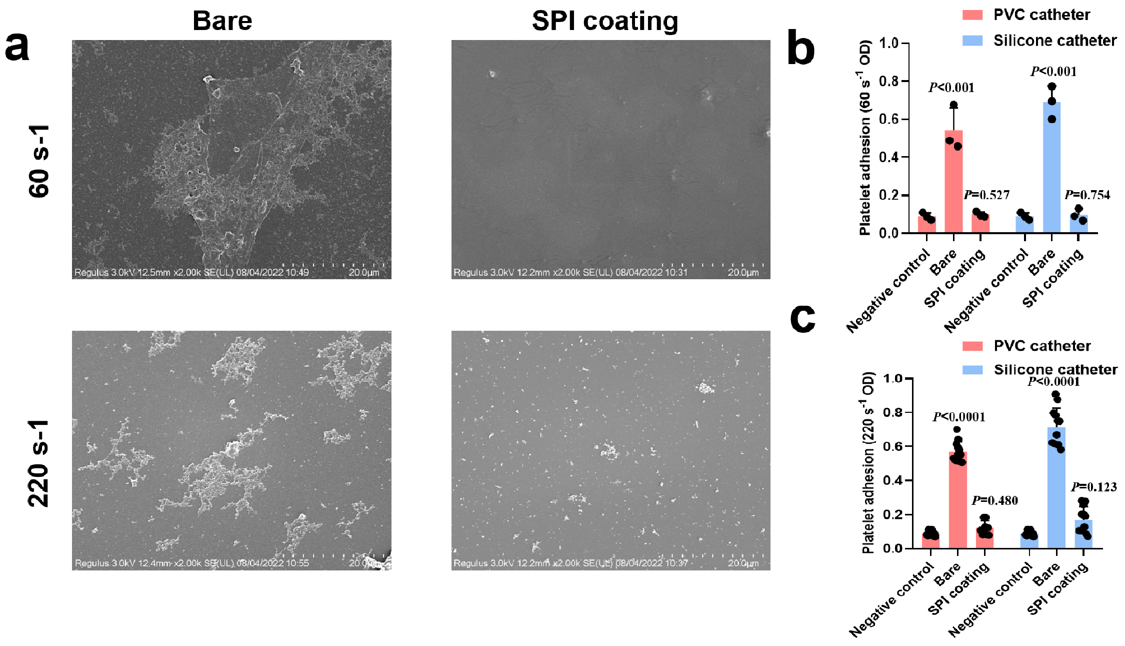

Supplementary figure 21: Platelet adhesion from platelet rich plasma under different shear rates. (a) Platelet adhesion on the surface of PVC catheter before and after SPI coating treatment under different shear rate observed by SEM. Representative images from PVC catheter are shown. (b) platelet adhesion on the surface of PVC catheter and silicone catheter before and after SPI coating treatment under 60 s^{-1} shear rate (all the values are expressed as mean ± SD, n = 3 biologically independent samples), and (c) platelet adhesion on the surface of PVC and silicone catheter before and after SPI coating treatment under 220 s^{-1} shear rate. The results of (b) and (c) are measured by LDH method [5] (all the values are expressed as mean ± SD, n = 12 biologically independent samples). One-way ANOVA was applied for intra-group comparison for each substrate with different coating. If significance was determined, post-hoc multiple comparison analysis was conducted with Tukey test. Exact p-values are provided in the corresponding figures, statistical significance was defined as $p < 0.05$.

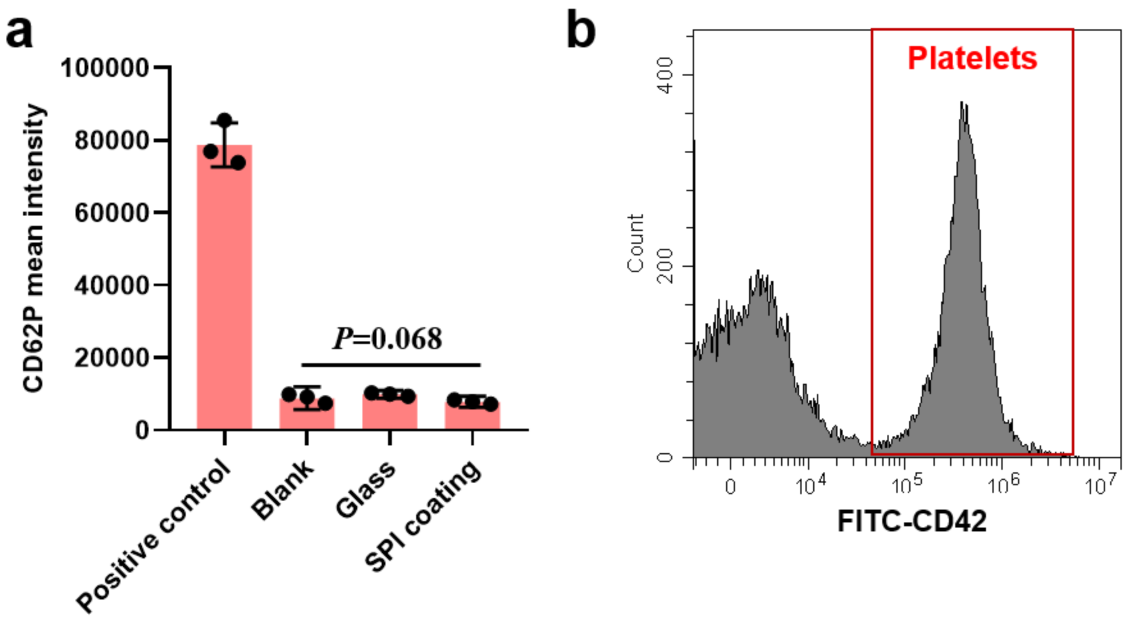

Supplementary figure 22: Platelet activation after incubation with glass vial with or without SPI coating in human platelet rich plasma measured by flow cytometry **(a)** and the gating strategy **(b).** Multiple comparisons were performed using one-way ANOVA. If significance was determined, post-hoc multiple comparison analysis was conducted with Tukey test. All the values are expressed as mean ± SD, n = 3 biologically independent samples. Exact p-values are provided in the corresponding figures, statistical significance was defined as $p < 0.05$. Here we do not detect PF4 generation because the PDA coating may significantly adsorb PF4, leading to wrong interpretation.

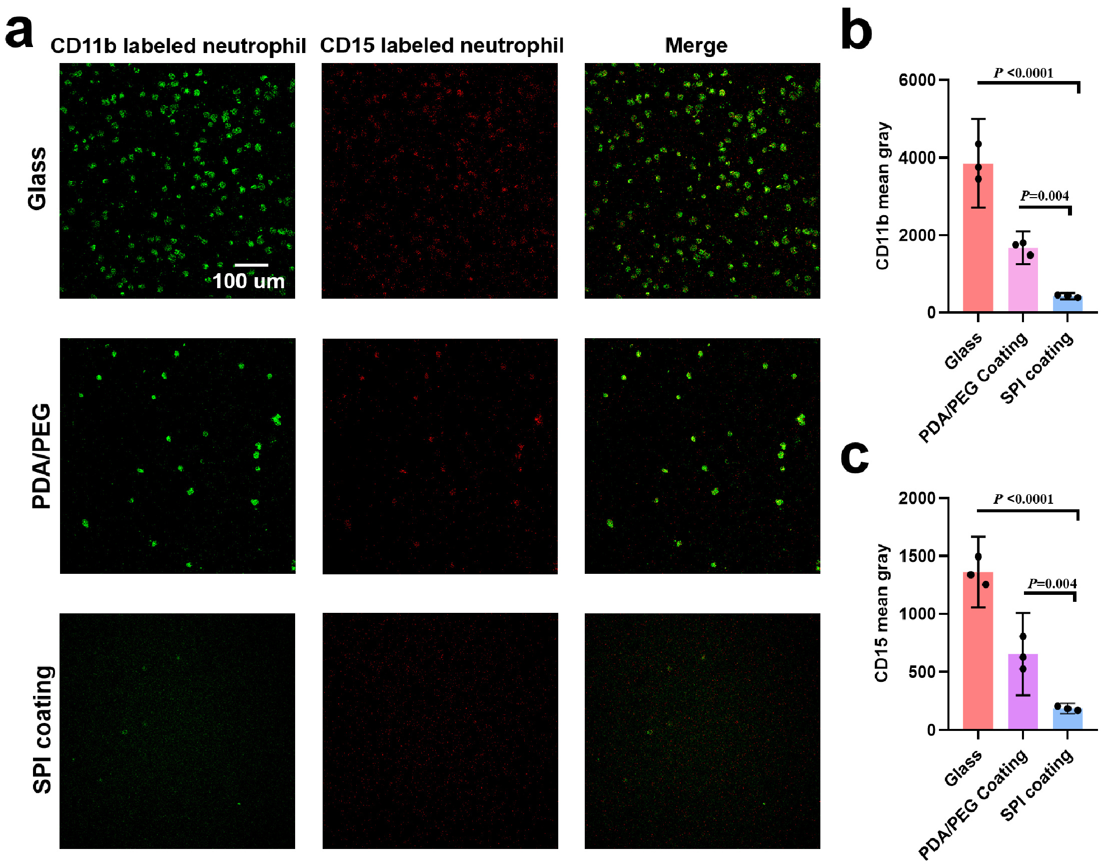

Supplementary figure 23: Neutrophil interaction with different surfaces. (a) Neutrophil adhesion and activation on bare, PDA/PEG or SPI coated glass surface observed by confocal microscope. Representative micrographs are shown. Green represents CD11b-labelled platelets (active state) and red represents CD15-labelled platelets (normal state), (b) CD11b mean gray and (c) CD15 mean gray on glass surface before or after PDA/PEG or SPI coating treatment. Three random images for each group with same brightness and contrast were selected for statistically measuring fluorescence intensity. Multiple comparisons were performed using one-way ANOVA. If significance was determined, post-hoc multiple comparison analysis was conducted with Tukey test. All values are expressed as the mean ± s.d. Exact p-values are provided in the corresponding figures, statistical significance was defined as $p < 0.05$.

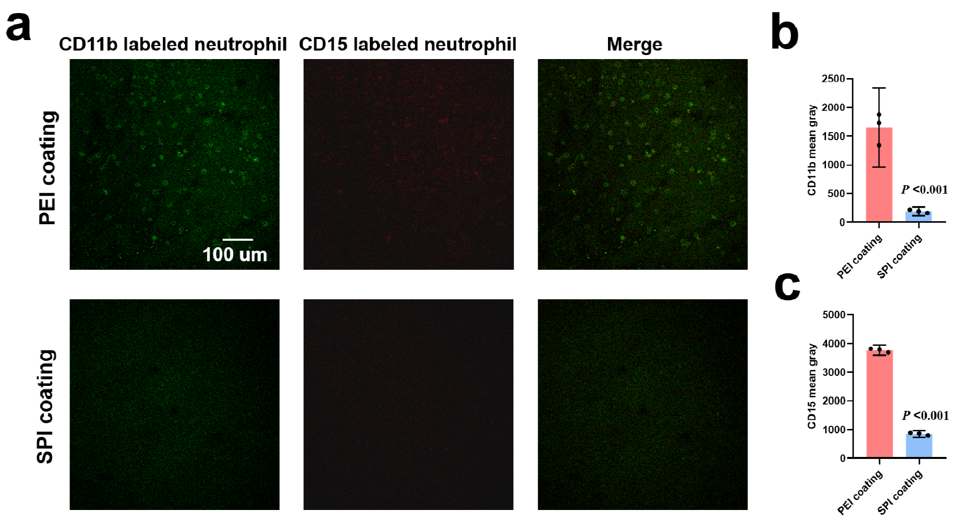

Supplementary figure 24: Neutrophil interaction with different surface coating. (a) Neutrophil adhesion and activation on glass surface after PEI or SPI coating treatment observed by confocal microscope. Representative images are given. Green represents CD11b-labelled platelets (active state) and red represents CD15-labelled platelets (normal state), (b) CD11b mean gray and (c) CD15 mean gray on glass surface after PEI or SPI coating treatment. Three random images for each group with same brightness and contrast were selected for statistically measuring fluorescence intensity. Unpaired, two-tailed student's t-test was applied. Positively charged surfaces tend to adhere neutrophil, while masked positively charged surfaces inhibit the interaction between neutrophil and the surface. All values are expressed as the mean ± s.d. Exact p-values are provided in the corresponding figures, statistical significance was defined as $p < 0.05$.

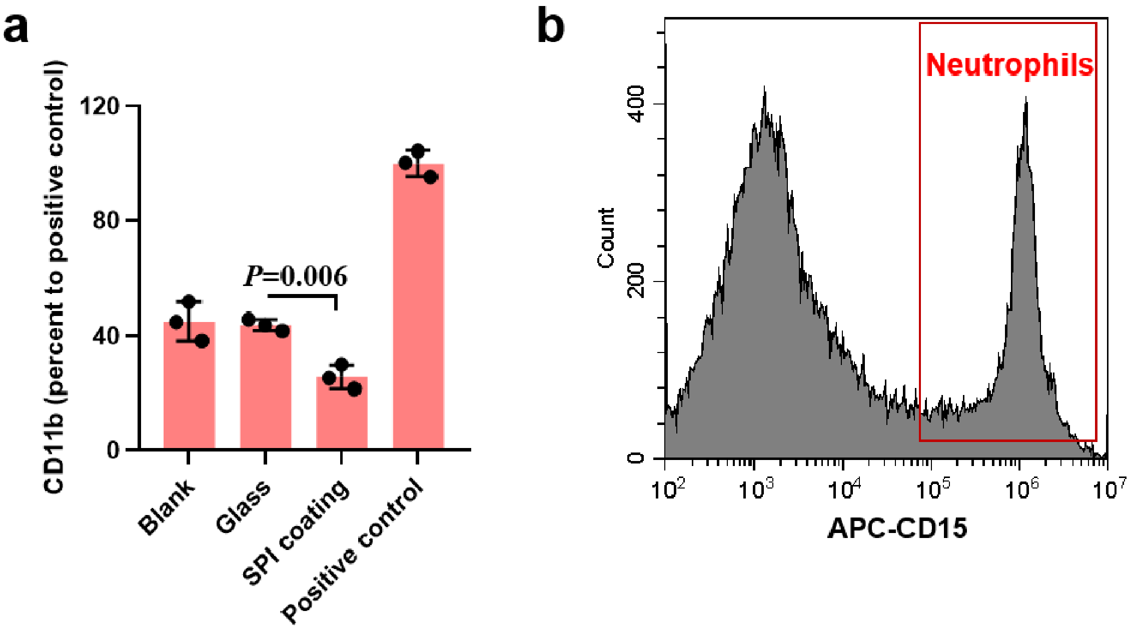

Supplementary figure 25: Neutrophil activation after incubation with glass vial with or without SPI coating treatment measured by flow cytometry **(a)** and the gating strategy **(b).** Multiple comparisons were performed using one-way ANOVA. If significance was determined, post-hoc multiple comparison analysis was conducted with Tukey test. All the values are expressed as mean ± SD, n = 3 biologically independent samples. Exact p-values are provided in the corresponding figures, statistical significance was defined as $p < 0.05$.

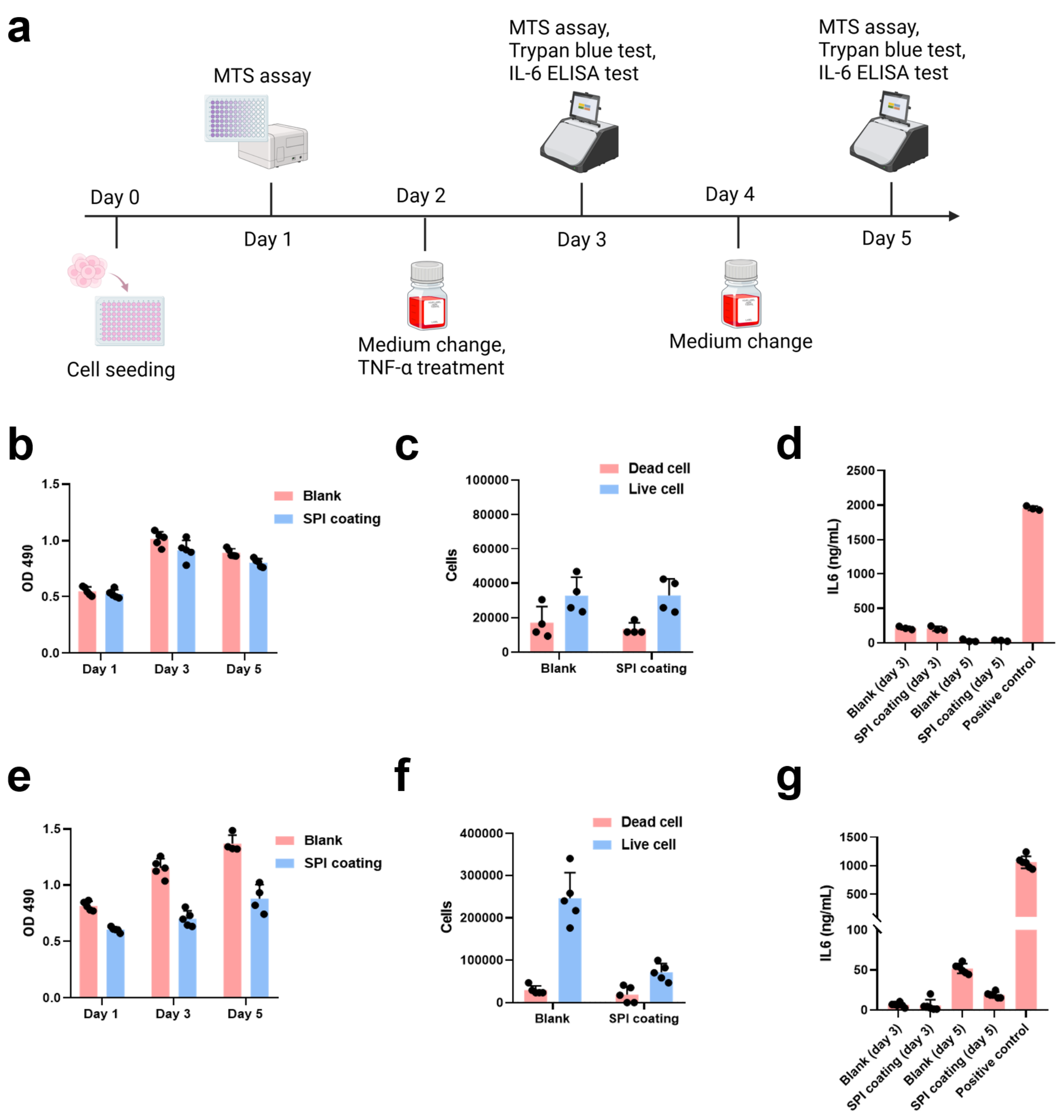

Supplementary figure 26: Cell adhesion and cytotoxicity of SPI coating. (a) Schematic diagram showing the experimental set-up to evaluate the cell proliferation and cytotoxicity after incubation with different surfaces. (b) MTS assay for the smooth muscle cells after incubation with different surfaces. The data showed the control and SPI coating showed similar cell viability based on OD measurements (n = 5 biologically independent samples). (c) Live-dead cell assay (Trypan blue) for the smooth muscle cells detached from different surfaces (n = 4 biologically independent samples). (d) IL-6 expression of the smooth muscle cells after incubation with different surfaces (n = 3 biologically independent samples). (e) MTS assay for the endothelial cells after incubation with different surfaces (n = 5 biologically independent samples). (f) Live-dead cell assay (Trypan blue) of the endothelial cells detached from different surfaces (n = 5 biologically independent samples). (g) IL-6 expression for the endothelial cells after incubation with different surfaces (n = 4 biologically independent samples). All the values are expressed as mean ± SD. The data confirms the non-toxic behavior of SPI coating in vitro.

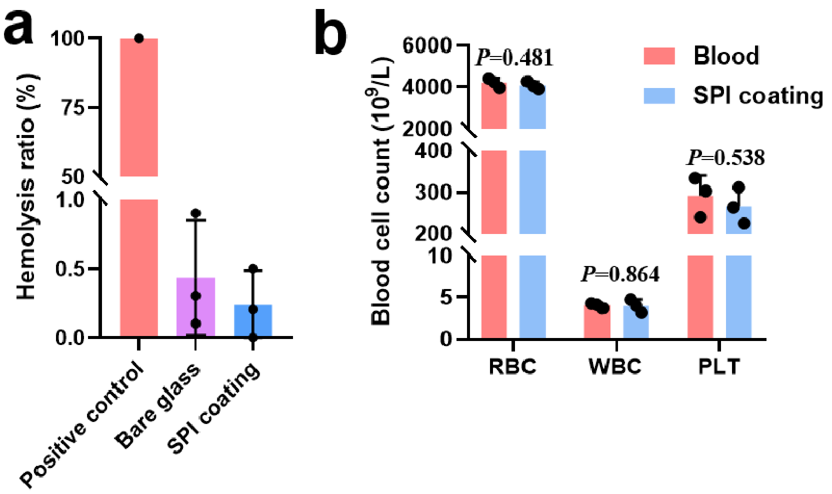

Supplementary figure 27: Hemolysis and blood cell count after incubation with SPI coating. (a) Hemolysis ratio of the glass samples before and after SPI coating treatment. The results show that the surfaces will not result in significant hemolysis (<1%) [7]. (b) Comparison of the blood cell count for blood and blood after incubating with SPI-coated glass vial. Unpaired, two-tailed student's t-test was applied for intergroup comparison. All the values are expressed as mean ± SD, n = 3 biologically independent sample. Exact p-values are provided in the corresponding figures, statistical significance was defined as $p < 0.05$. The SPI coating will not result in significant influence on blood cell count.

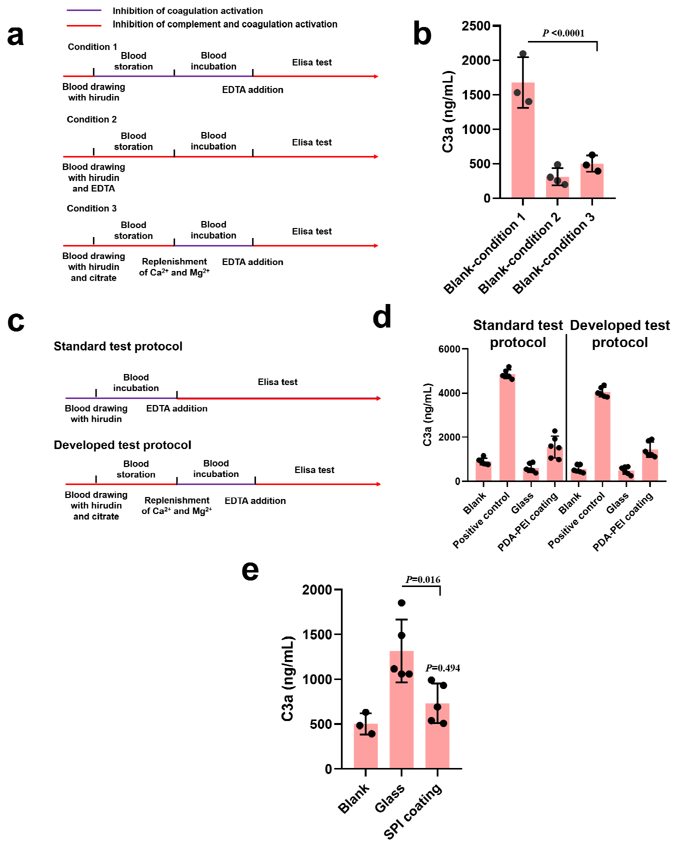

Supplementary figure 28: Complement activation by different coatings-method development. (a) In this work, we developed the synergistic anticoagulation method of hirudin and sodium citrate for evaluating the complement activation, in which hirudin inhibits thrombogenesis without inhibiting complement activation and sodium citrate inhibits both complement activation and coagulation activation, but the inhibitory effect of sodium citrate on the coagulation and complement systems is lost after re-calcification and re-addition of magnesium. (b) The results clearly show that this anticoagulant method can significantly reduce background without inhibiting complement activation during the incubation with materials (n = 3 biologically independent samples for condition 1 and 3, n = 4 biologically independent sample for condition 2). (c) The illustration for standard test protocol and the developed test protocol. For the standard test protocol, the blood was gently collected with vacuum tube without anticoagulant, then the blood was incubated with different surface straightway (blank: whole blood incubated with PP tube), after 30 mins incubation, EDTA was added to terminate the complement activation, and the plasma samples were collected by 2,000 g centrifugation. (d) The C3a generation measured by different test protocols showed similar trend for different substrate (n = 6 biologically independent samples). (e) SPI coating can inhibit the complement activation induced by the glass surface (n = 3 biologically independent samples for blank control, n = 5 biologically independent samples for glass and SPI coating). Multiple comparisons were performed using one-way ANOVA. If significance was determined, post-hoc multiple comparison analysis was conducted with Tukey test. All the values are expressed as mean ± SD. Exact p-values are provided in the corresponding figures, statistical significance was defined as $p < 0.05$. This anticoagulation method will also be used in subsequent complement activation experiments.

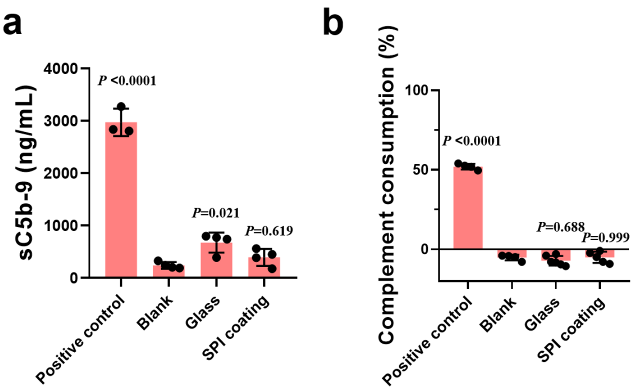

Supplementary figure 29: Complement activation by different surfaces measured by the developed protocol. (a) Concentration of human complement C5b-9 generated after incubating the glass vial or SPI-coated glass vial with whole blood (n = 3 biologically independent samples for positive control, n = 4 biologically independent samples for blank, glass and SPI coating). (b) Effect of the surface of bare glass surface or SPI coated glass surface on complement consumption (evaluated by the sheep erythrocyte based hemolytic assay) (n = 4 biologically independent samples for positive control and blank control, n = 6 biologically independent samples for blank and n = 5 biologically independent samples SPI coating). Multiple comparisons were performed using one-way ANOVA. If significance was determined, post-hoc multiple comparison analysis was conducted with Tukey test. All the values are expressed as mean ± SD. Exact p-values are provided in the corresponding figures, statistical significance was defined as $p < 0.05$.

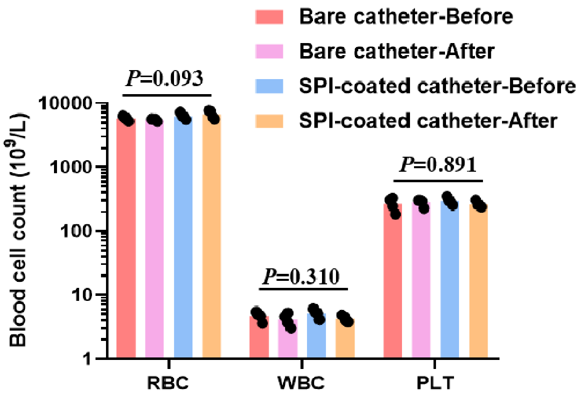

Supplementary figure 30: Comparison of the blood cell count for animals before and after the shunt model experiment. All the values are expressed as mean ± SD, n = 3 biologically independent samples. One-way ANOVA was applied for intra-group comparison for each cell. If significance was determined, post-hoc multiple comparison analysis was conducted with Tukey test. Exact p-values are provided in the corresponding figures, statistical significance was defined as $p < 0.05$. **It is worth noting that for the SPI coating group, the blood sample was obtained from the catheter, whereas for the bare group after treatment, the blood sample was obtained directly from the vessel (the catheter was completely blocked and no blood sample could be collected).** This may indicate that SPI-coated surfaces will not lead to significant coagulation activation, as coagulation activation tends to lead to platelet and neutrophil activation, and these activated cells are more likely to adhere to the thrombus.

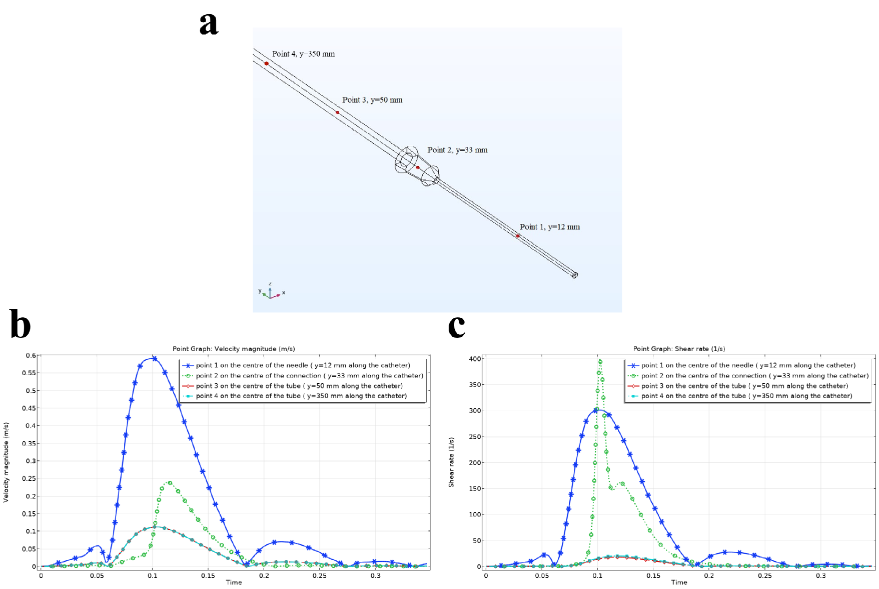

Supplementary figure 31: Computational fluid dynamics (CFD) model for the catheter-indwelling needle connection. The velocity (m/s) and shear rate (1/s) on selected points as shown in (a). (b) The velocity magnitude on different points for the catheter. (c) The shear rate on different points for the catheter. The velocity and shear rate along the catheter remain almost constant along the length of the tube, with the exception of the connector part with indwelling needle. The parameters for the Carreau model were adapted from[27, 28]. Density: 1056 [kg*m^{-3}], zero shear rate viscosity: 0.01937[Pa*s], infinite shear rate viscosity: 0.00345[Pa*s], relaxation time: 1.3954[s], power index 0.4816.

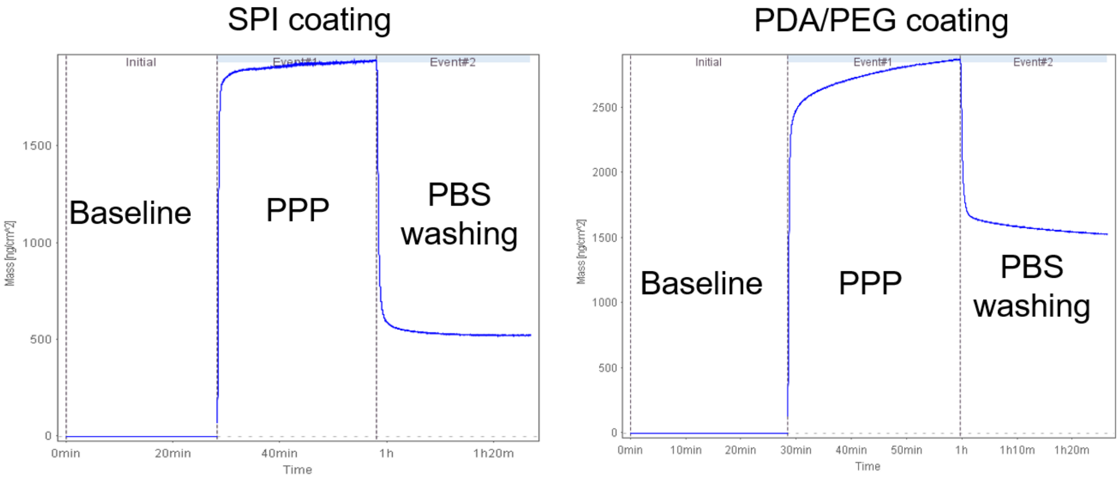

Supplementary figure 32: QCM spectrum for plasma protein adsorption on SPI coating and PDA/PEG coating from human plasma. The loosely bound protein can be washed away by PBS rinsing (flow rate: 100 μL/min, temperature: 37 °C). The experiment was performed independently at least in duplicate and similar results were obtained; the representative results were given here.

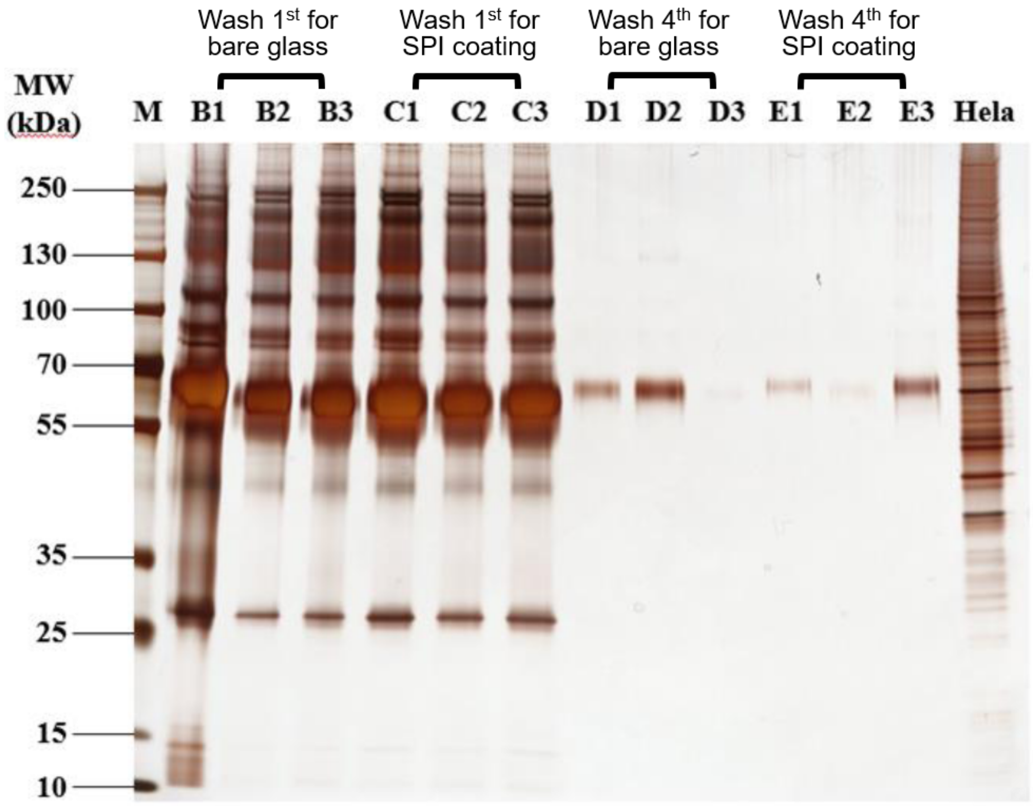

Supplementary figure 33: SDA-PAGE of adsorbed plasma proteins. Protein adsorption amount on bare glass and SPI-coated glass surface after different wash step measured by non-reduction SDS-PAGE. n = 3 biologically independent samples. It can be observed that different washing processes significantly affect the protein adsorption on the material surface. In turn, the loosely bound proteins on the surface can likewise affect the functionality of the surface. Furthermore, the protein adsorption amounts on the bare glass and SPI-coated glass surfaces are not significantly different after the same washing process, which may indicate that the protein composition on the surface plays an important role.

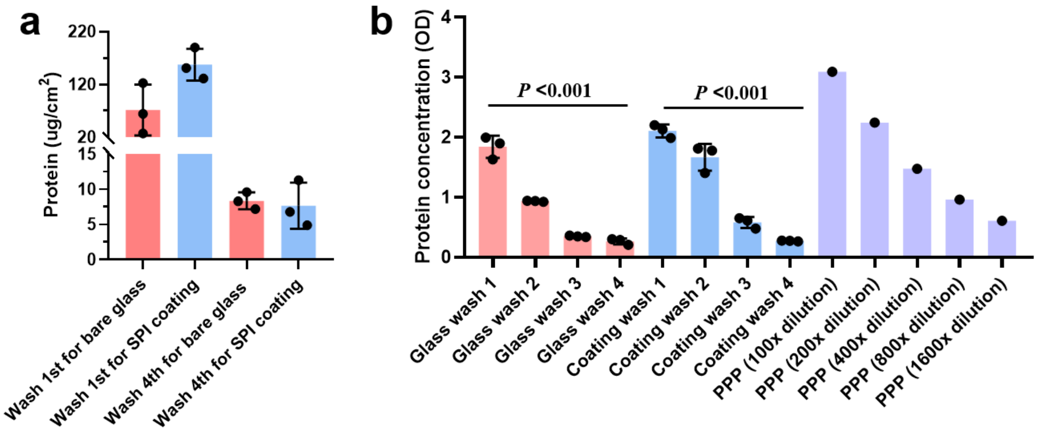

Supplementary figure 34: Protein adsorption on different surfaces from human plasma. The amount of protein adsorption amount on bare glass and SPI-coated glass surface after different wash step (a) and the comparison with that on diluted PPP (b) measured by BCA kit. All the values are expressed as mean ± SD, n = 3 biologically independent samples for different wash steps. One-way ANOVA was applied for intra-group comparison for each sample with different washing process. If significance was determined, post-hoc multiple comparison analysis was conducted with Tukey test. Exact p-values are provided in the corresponding figures, statistical significance was defined as $p < 0.05$. The protein amounts on the bare glass and SPI-coated glass surfaces are not significantly different after the same washing process corresponding with **Fig. S33**. As a reference, the mass of loosely bound protein on the surface is approximately 5 ug/cm^2 using diluted plasma as a reference (assuming a plasma protein concentration of 60 mg/mL).

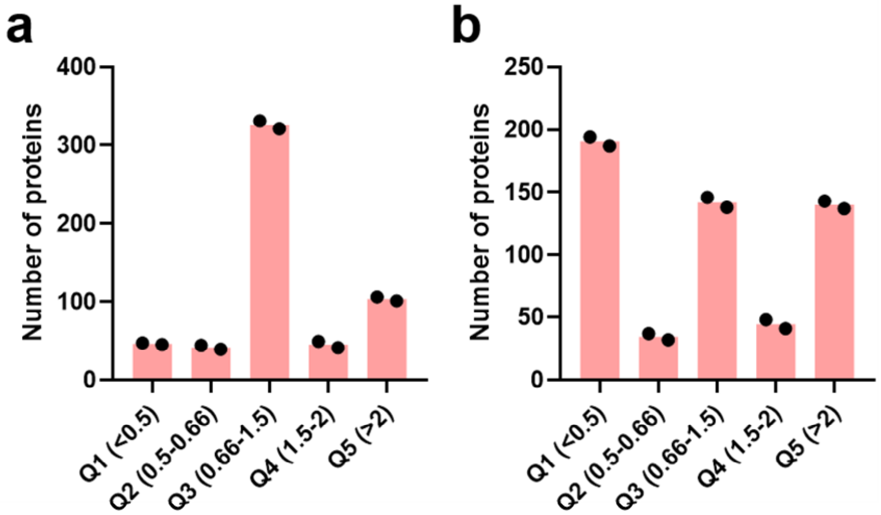

Supplementary figure 35: Protein adsorption from human plasma evaluated using proteomic techniques. Protein composition on bare glass and SPI-coated glass surface after 1st (a) and 4th (b) wash step measured by proteomic techniques. The composition of proteins on the surface changes significantly after different washing processes, which means false conclusions may be obtained if only focus on the tightly adsorbed proteins on the surface. n = 2 biologically independent samples.

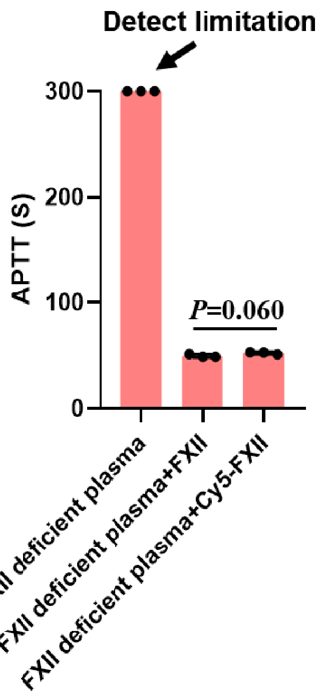

Supplementary figure 36: aPTT for FXII deficient plasma after replenishing with FXII and cy5-labelled FXII. All the values are expressed as mean ± SD, n = 3 biologically independent samples. One-way ANOVA was applied. If significance was determined, post-hoc multiple comparison analysis was conducted with Tukey test. Exact p-values are provided in the corresponding figures, statistical significance was defined as $p < 0.05$.

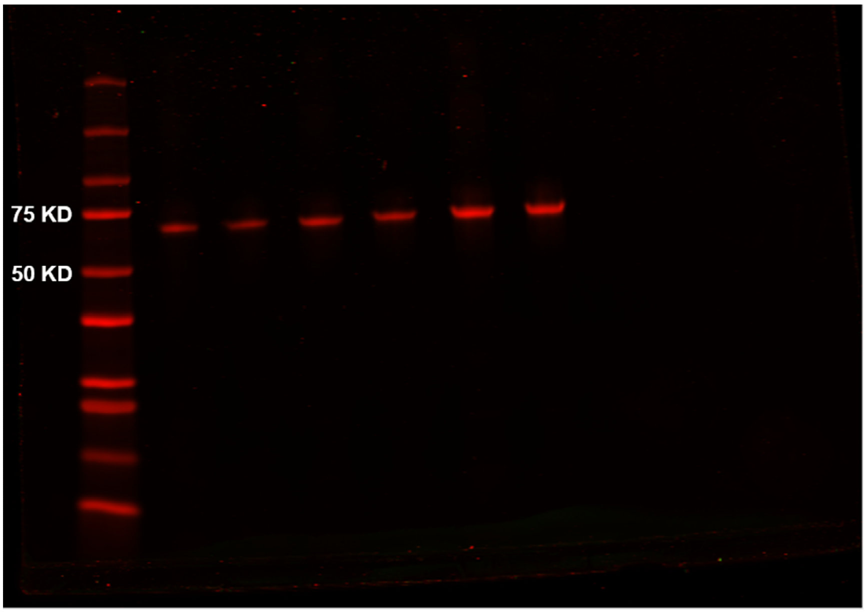

Supplementary figure 37: Reduced SDS-PAGE for FXII and Fluor-labelled FXII. From left to right: marker, FXII (0.5 μg), cy5-labelled FXII (0.5 μg), FXII (1 μg), cy5-labelled FXII (1 μg), FXII (2 μg), and cy5-labelled FXII (2 μg). The non-reductant SDS-PAGE show similar result as reductant SDS-PAGE (data not shown here). Combined with aPTT and SDS-PAGE results, it's safe to say the function of FXII does not change after fluorescence labeling and purification. At least 2 times each experiment was repeated independently with similar results.

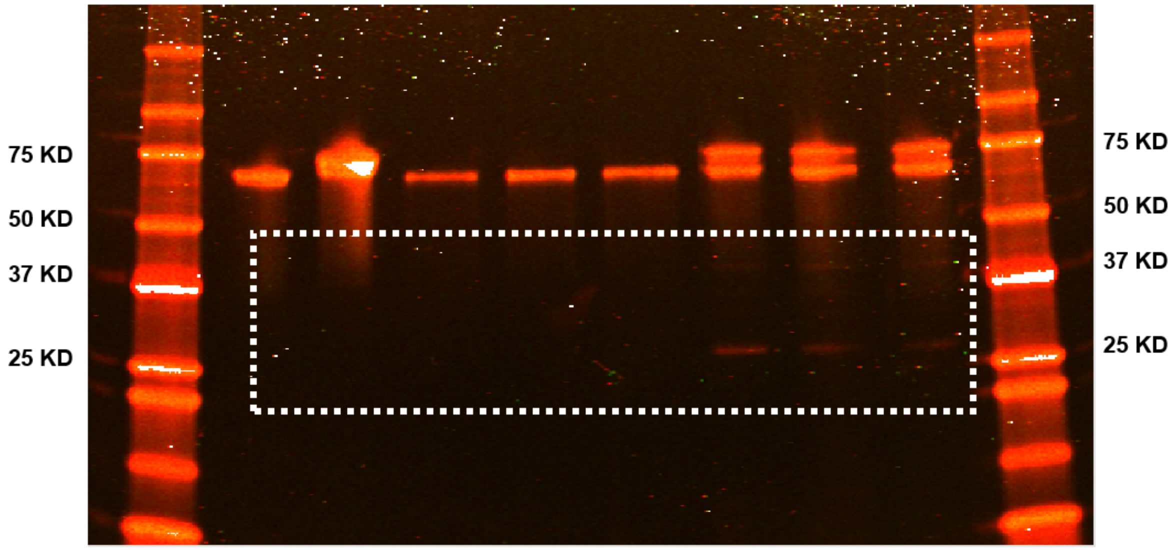

Supplementary figure 38: Reduced SDS-PAGE for the *in vitro* simulation. From left to right: marker, FXII (without incubation, 1 µg), PK (without incubation, 1 µg), FXII (incubated with glass, 0.5 µg), FXII (incubated with HPG-PEG coating, 0.5 µg), FXII (incubated with SPI coating, 0.5 µg), mixture of FXII and PK (0.5 µg of each, incubated with glass), mixture of FXII and PK (0.5 µg of each, incubated with HPG-PEG coating), mixture of FXII and PK (0.5 µg of each, incubated with SPI coating), marker. The distribution of FXIIa and PKa is circled in white. The incubation of FXII with surface cannot lead to the cleavage. Compared to glass and HPG-PEG coatings, SPI coating can significantly reduce the contact activation. At least 2 times each experiment was repeated independently with similar results.

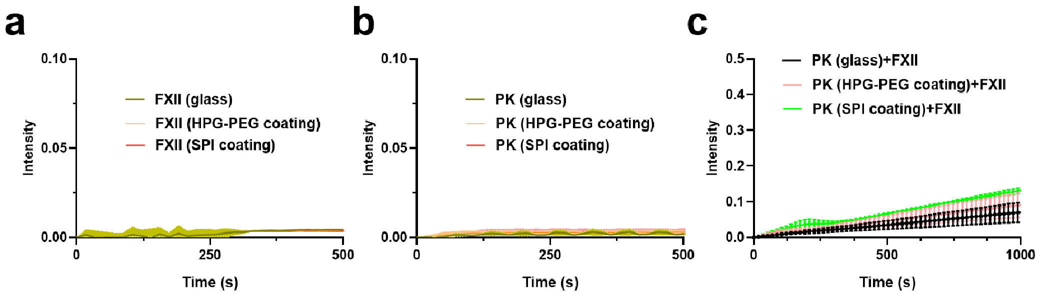

Supplementary figure 39: FXII or PK activation after interaction with different surfaces. (a) FXII incubated with surfaces cannot cleave S-2302 effectively. Combined with SDS-PAGE results, it's safe to say the FXII is not activated after surface incubation. (b) PK incubated with surfaces cannot cleave S-2302 effectively. (c) PK solution (400 nM, final concentration) was first incubated with glass vials before and after HPG-PEG and SPI modification for 10 min, then the solution was transferred to a new PP tube and FXII (400 nM, final concentration) was added and co-incubated for 5 min, then the cleavage efficiency of S-2302 for the mixture was recorded. n = 3 biologically independent samples, all values are expressed as the mean ± s.d.

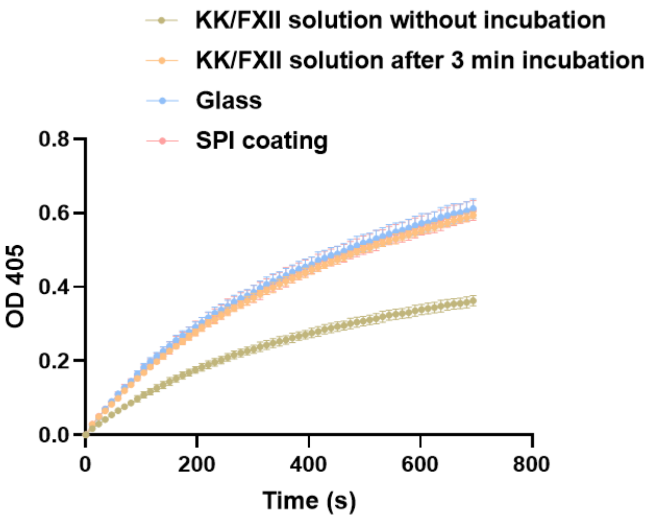

Supplementary figure 40: Cleavage efficiency of S-2302 by an in vitro simulated contact initiation system (3 µg/100 µL of FXII and 1 µg/100 µL KK in PBS buffer) after incubation with different surfaces. n = 3 biologically independent samples, all values are expressed as the mean ± s.d.

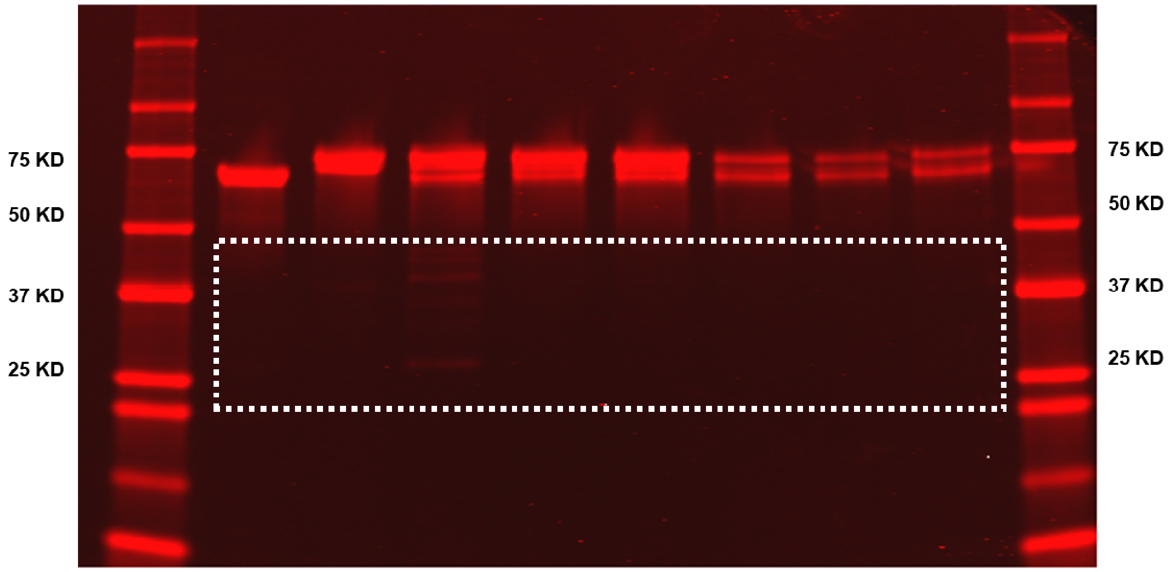

Supplementary figure 41: Reduced SDS-PAGE for the *in vitro* simulation. From left to right: marker, FXII (without incubation, 1 µg), PK (without incubation, 1 µg), mixture of PK and glass incubated FXII (0.5 µg of each), mixture of PK and HPG-PEG coating incubated FXII (0.5 µg of each), mixture of PK and SPI coating incubated FXII (0.5 µg of each), mixture of FXII and glass incubated PK (0.5 µg of each), mixture of FXII and HPG-PEG incubated PK (0.5 µg of each), mixture of FXII and SPI coating incubated PK (0.5 µg of each), marker. Compared to the structural change of FXII induced by the surfaces, the PK-surface interaction does not contribute much to contact activation. At least 2 times each experiment was repeated independently with similar results.

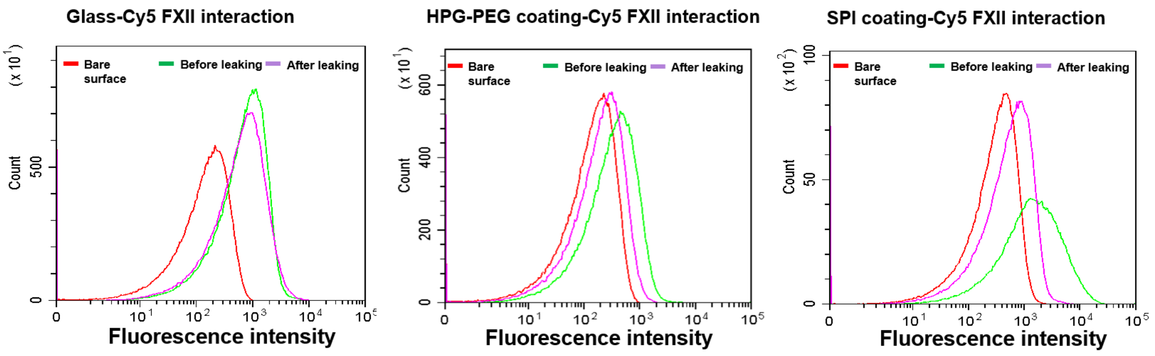

Supplementary figure 42: Fluorescence intensity of externally added Cy5-labeled FXII on the bare, HPG-PEG or SPI coated glass surface before Cy5-labeled FXII adsorption, after Cy5-labeled FXII adsorption, and after Cy5-labeled FXII desorption process (500,000 events were recorded for each sample in FXII-deficient plasma). n = 3 biologically independent samples.

Supplementary tables:

Supplementary table 1: Results from proteomic analysis of the coating incubated with plasma

Relative abundance of top 10 most-abundant proteins identified from normal human plasma (PPP) intensity ratios compared with SPI coating incubated plasma (T-PPP). Proteins were grouped according to biological processes of the blood system, and then ranked by abundance from highest to lowest. Relative abundance of proteins in different plasma was given to facilitate the comparison of difference. n = 2 biologically independent samples, the results are expressed as mean (SD).

	Protein description	Relative abundance	PPP Intensity/T-PPP Intensity
**Acute Phase**			
**1**	Ceruloplasmin	1.02 (0.02)	1.13 (0.10)
**2**	Fibronectin	0.32 (0.14)	0.23 (0.01)
**3**	Alpha-1-acid glycoprotein 1	0.98 (0.05)	1.14 (0.20)
**4**	Angiotensinogen	1.03 (0.18)	1.11 (0.06)
**5**	Alpha-2-macroglobulin	1.23 (0.19)	1.58 (0.13)
**6**	Serum amyloid P-component	0.98 (0.03)	0.94 (0.07)
**7**	Alpha-1-antitrypsin	1.04 (0.03)	1.01 (0.01)
**8**	Haptoglobin	1.38 (0.33)	1.85 (0.02)
**9**	SAA2-SAA4 readthrough	0.94 (0.27)	0.80 (0.09)
**10**	Alpha-1-acid glycoprotein 2	1.25 (0.23)	1.84 (0.31)
**Coagulation**			
**1**	Plasminogen	0.97 (0.01)	0.95 (0.01)
**2**	Antithrombin-III	1.04 (0.09)	1.12 (0.04)
**3**	Kininogen-1	1.06 (0.02)	1.09 (0.01)
**4**	Prothrombin	0.97 (0.09)	1.01 (0.10)
**5**	Histidine-rich glycoprotein	0.99 (0.02)	0.85 (0.01)
**6**	Alpha-2-antiplasmin	1.02 (0.01)	0.93 (0.04)
**7**	Heparin cofactor 2	0.93 (0.01)	1.03 (0.07)
**8**	Plasma kallikrein	1.01 (0.07)	1.00 (0.02)
**9**	von Willebrand factor	0.92 (0.21)	0.73 (0.02)
**10**	Coagulation factor V	1.25 (0.04)	0.95 (0.04)
**Complement**			
**1**	Complement C3	0.93 (0.04)	0.95 (0.02)
**2**	Complement C4-A	0.95 (0.09)	0.94 (0.01)
**3**	Complement factor H	0.92 (0.16)	0.94 (0.08)
**4**	C3/C5 convertase	1.00 (0.12)	1.07 (0.05)
**5**	Plasma protease C1 inhibitor	0.93 (0.12)	0.90 (0.07)
**6**	C4b-binding protein	1.03 (0.05)	1.07 (0.01)
**7**	Complement C5	1.04 (0.01)	1.00 (0.03)
**8**	Complement subcomponent C1r	0.95 (0.15)	0.90 (0.06)
**9**	Complement component C7	0.97 (0.01)	0.95 (0.01)
**10**	Complement factor I	0.99 (0.10)	1.09 (0.21)
**Immunoglobulins**			
**1**	Immunoglobulin heavy constant gamma 1	1.49 (0.31)	2.03 (0.21)

**2**	Immunoglobulin heavy constant alpha 1	1.09 (0.01)	1.20 (0.09)
**3**	Immunoglobulin heavy variable 3-7	1.26 (0.24)	1.44 (0.14)
**4**	Immunoglobulin heavy constant mu	1.40 (0.17)	1.99 (0.01)
**5**	Immunoglobulin heavy constant delta	0.94 (0.22)	0.91 (0.05)
**6**	Immunoglobulin lambda constant 2	1.50 (0.24)	4.22 (2.86)
**7**	Immunoglobulin kappa variable 1-27	1.43 (0.75)	1.88 (0.25)
**8**	Immunoglobulin lambda-like polypeptide 5	1.26 (0.23)	1.54 (0.02)
**9**	Immunoglobulin heavy variable 3-15	1.21 (0.15)	1.41 (0.07)
**10**	Low affinity immunoglobulin gamma Fc region receptor III	0.83 (0.45)	0.75 (0.10)
**Lipoproteins**			
**1**	Apolipoprotein A-I	0.92 (0.04)	0.97 (0.07)
**2**	Apolipoprotein B-100	0.90 (0.01)	0.86 (0.03)
**3**	Apolipoprotein A-IV	0.95 (0.07)	0.88 (0.06)
**4**	Apolipoprotein A-II	1.05 (0.23)	1.22 (0.02)
**5**	Apolipoprotein E	0.92 (0.10)	0.81 (0.03)
**6**	Apolipoprotein C-I	1.14 (0.10)	1.17 (0.11)
**7**	Apolipoprotein(a)	1.00 (0.12)	0.98 (0.01)
**8**	Apolipoprotein C-II	0.93 (0.14)	0.93 (0.01)
**9**	Apolipoprotein D	0.92 (0.02)	0.88 (0.13)
**10**	Apolipoprotein C-III	0.87 (0.21)	0.86 (0.12)

Supplementary table 2: The body weights and temperature of the rabbits.

Rabbit	Body weight (kg)	Abnormal body temperature
Rabbit 1	2.6	N/A
Rabbit 2	2.65	N/A
Rabbit 3	2.63	N/A
Rabbit 4	2.65	N/A
Rabbit 5	2.6	N/A
Rabbit 6	3.1	N/A
Rabbit 7	2.77	N/A
Rabbit 8	2.66	N/A
Rabbit 9	3.12	N/A
Rabbit 10	2.73	N/A

Supplementary table 3: Biological parameters levels for the shunt model treated with bare catheter or SPI-coated catheter before and after treatment. *ALP, alkaline phosphatase; ALT, alanine aminotransferase; AST, aspartate aminotransferase; GGT, gamma-glutamyl transferase; UREA, urea; CREA, serum creatinine; TP, total protein; ALB, albumin; LDH: lactic dehydrogenase; C4, complement component 4; C3, complement component 3; IGA, immunoglobulin A; IGG, immunoglobulin G; IGM, immunoglobulin M; GLU, glucose; CHOL, cholesterol; TG, triglyceride; K, serum potassium; Na, serum sodium; Cl, serum chlorine; P, serum phosphorus; UA, uric acid; HDL, high-density lipoprotein; LDL, low-density lipoprotein; CA, serum calcium; DBIL, direct bilirubin; TBIL, total bilirubin. n = 4 biologically independent samples.

Tests and units	Bare catheter		SPI-coated catheter	
	Before treatment	After treatment	Before treatment	After treatment
**ALP u/L**	154.90	157.18	111.48	108.40
**ALT u/L**	52.75	53.50	41.50	40.00
**AST u/L**	25.98	25.85	20.65	19.05
**GGT u/L**	6.33	6.58	8.30	7.78
**ALB g/L**	34.53	35.33	46.78	45.08
**LDH u/L**	155.00	241.75	67.00	64.00
**UREA mmol/L**	5.19	7.01	6.41	6.74
**CREA umol/L**	53.60	40.65	75.45	76.43
**TP g/L**	51.48	51.35	57.68	55.25
**C4 g/L**	0.04	0.04	0.05	0.05
**IGA g/L**	0.00	0.00	0.00	0.00
**IGG g/L**	3.01	3.00	3.80	3.71
**IGM g/L**	0.17	0.17	0.16	0.16
**C3 g/L**	0.16	0.16	0.21	0.20
**GLU mmol/L**	14.75	15.47	16.79	17.93
**CHOL mmol/L**	0.68	0.95	0.86	0.81
**TG mmol/L**	0.53	0.52	0.49	0.51
**K mmol/L**	3.35	3.17	3.26	3.14
**Na mmol/L**	141.25	142.25	144.50	142.50
**Cl mmol/L**	96.53	93.80	101.85	100.35
**P mmol/L**	2.12	2.61	2.37	2.42
**UA umol/L**	35.00	8.00	13.75	13.00
**HDL mmol/L**	0.41	0.41	0.60	0.57
**LDL mmol/L**	0.32	0.34	0.25	0.25
**CA mmol/L**	2.56	2.50	3.12	3.01
**DBIL umol/L**	0.08	0.13	0.38	0.40
**TBIL umol/L**	0.53	0.43	0.43	0.38

Supplementary table 4: Relative abundance of top 10 most-abundant proteins in adsorbed protein corona from human plasma identified on bare glass and SPI coating after 1st wash. Proteins were grouped according to biological processes of the blood system, and then ranked by abundance from highest to lowest. Relative abundance of proteins at two stages was given to facilitate the comparison of difference. For unique protein existing in each sample, the corresponding relative abundance in another sample, if detected (but not in top 10 most-abundant), was provided. [a], relative abundance of proteins in protein corona on SPI coating after 1st wash; [b], relative abundance of proteins in protein corona on bare glass after 1st wash. n = 2 biologically independent samples.

#	Bare glass after 1st wash		SPI coating after 1st wash	
	**Protein description**	**Relative abundance**	**Protein description**	**Relative abundance**
**Acute Phase**				
**1**	Alpha-2-macroglobulin	1.007	Alpha-2-macroglobulin	1
**2**	Haptoglobin	1.0165	Haptoglobin	0.9885
**3**	Alpha-1-antitrypsin	1.0355	Alpha-1-antitrypsin	0.9655
**4**	Alpha-1-acid glycoprotein 1	1.066	Alpha-1-acid glycoprotein 1	0.931
**5**	Fibronectin	1.016	Fibronectin	0.9885
**6**	Ceruloplasmin	0.95	Ceruloplasmin	1.0675
**7**	Serum amyloid P-component	1.026	Serum amyloid P-component	0.979
**8**	Alpha-1-acid glycoprotein 2	0.8805	Alpha-1-acid glycoprotein 2	1.1495
**9**	SAA2-SAA4 readthrough	1.042	SAA2-SAA4 readthrough	0.961
**10**	LPS-binding protein	0.625	LPS-binding protein	1.4635
**Coagulation**				
**1**	Antithrombin-III	0.958	Antithrombin-III	1.0595
**2**	Plasminogen	1.086	Plasminogen	0.906
**3**	Kininogen-1	1.1185	Kininogen-1	0.867
**4**	Prothrombin	1.0325	Prothrombin	0.9705
**5**	Alpha-2-antiplasmin	0.9075	Alpha-2-antiplasmin	1.1205
**6**	Heparin cofactor 2	0.961	Heparin cofactor 2	1.0555
**7**	Plasma kallikrein	1.268	Histidine-rich glycoprotein	1.6985 (0.42[b])
**8**	Kallistatin	1.0035	Kallistatin	1.006
**9**	Protein S	1.094 (0.89[a])	Coagulation factor XII	1.6605 (0.46[b])
**10**	Carboxypeptidase N subunit 2	0.934 (1.09[a])	Plasma kallikrein	0.69
**Complement**				
**1**	Complement C3	0.9635	Complement C3	1.052
**2**	Complement C4-A	0.9645	Complement C4-A	1.0515
**3**	Complement factor H	0.994	Complement factor H	1.0165
**4**	Plasma protease C1 inhibitor	0.8965	Plasma protease C1 inhibitor	1.1315
**5**	C4b-binding protein	0.9555	C4b-binding protein	1.0635
**6**	Complement C5	1.1315	C3/C5 convertase	0.9755
**7**	C3/C5 convertase	1.028	Complement C5	0.8515
**8**	Complement component C9	1.0585	Clusterin	1.1305
**9**	Clusterin	0.8985	Complement component C9	0.9385
**10**	Complement component C7	1.199	Complement component C7	0.7705
**Immunoglobulins**				
**1**	Immunoglobulin heavy constant gamma 1	0.942	Immunoglobulin heavy constant gamma 1	1.0785
**2**	Immunoglobulin heavy constant alpha 1	0.9095	Immunoglobulin heavy constant alpha 1	1.1155
**3**	Immunoglobulin kappa constant	1.0245	Immunoglobulin heavy constant mu	1.08
**4**	Immunoglobulin heavy constant mu	0.941	Immunoglobulin kappa constant	0.9835
**5**	Immunoglobulin lambda-like polypeptide 5	1.0655	Immunoglobulin lambda-like polypeptide 5	0.9275
**6**	Immunoglobulin heavy constant	0.83	Immunoglobulin heavy constant	1.2115

	gamma 2		gamma 2	
**7**	Immunoglobulin heavy variable 3-7	1.096	Immunoglobulin heavy variable 4-39	1.2275
**8**	Immunoglobulin heavy variable 4-39	0.812	Immunoglobulin heavy variable 3-7	0.8925
**9**	Immunoglobulin lambda constant 3	1.6015 (0.293[a])	Immunoglobulin kappa variable 2D-29	1.188 (0.851[b])
**10**	Immunoglobulin heavy variable 4-4	1.1165 (0.865[a])	Immunoglobulin kappa variable 3-20	1.0575 (0.957[b])
**Lipoproteins**				
**1**	Apolipoprotein A-I	0.904	Apolipoprotein A-I	1.123
**2**	Apolipoprotein B-100	0.875	Apolipoprotein B-100	1.157
**3**	Apolipoprotein A-IV	1.087	Apolipoprotein A-IV	0.905
**4**	Apolipoprotein C-III	1.0695	Apolipoprotein C-III	0.9275
**5**	Apolipoprotein A-II	1.163	Apolipoprotein D	1.153
**6**	Apolipoprotein C-I	1.0425	Apolipoprotein C-I	0.957
**7**	Apolipoprotein E	1.142	Apolipoprotein A-II	0.815
**8**	Apolipoprotein D	0.879	Apolipoprotein E	0.84
**9**	Apolipoprotein C-II	1.2305	Apolipoprotein L1	1.123
**10**	Apolipoprotein L1	0.9045	Apolipoprotein C-II	0.734

Supplementary table 5: Relative abundance of top 10 most-abundant proteins in adsorbed protein corona from human plasma identified on bare glass and SPI coating after 4th wash. Proteins were grouped according to biological processes of the blood system, and then ranked by abundance from highest to lowest. Relative abundance of proteins at two stages was given to facilitate the comparison of difference. For unique protein existing in each sample, the corresponding relative abundance in another sample, if detected (but not in top 10 most-abundant), was provided. [a], relative abundance of corona proteins in SPI coating after 4th wash; [b], relative abundance of proteins in protein corona on bare glass after 1st wash. n = 2 biologically independent samples.

#	Bare glass after 4th wash		SPI coating after 4th wash	
	**Protein description**	**Relative abundance**	**Protein description**	**Relative abundance**
**Acute Phase**				
**1**	Haptoglobin	0.98	Haptoglobin	1.02
**2**	Alpha-2-macroglobulin	1.39	Alpha-2-macroglobulin	0.69
**3**	Alpha-1-antitrypsin	1.42	Alpha-1-acid glycoprotein 1	1.21
**4**	Alpha-1-acid glycoprotein 1	0.76	Alpha-1-antitrypsin	0.66
**5**	Ceruloplasmin	1.49	Ceruloplasmin	0.61
**6**	Fibronectin	1.37	Fibronectin	0.70
**7**	Serum amyloid P-component	1.21	Serum amyloid P-component	0.84
**8**	Alpha-1-acid glycoprotein 2	0.99	Alpha-1-acid glycoprotein 2	1.02
**9**	SAA2-SAA4 readthrough	1.74	Heat shock cognate	1.72
**10**	LPS-binding protein	2.17 (0.06[a])	SAA2-SAA4 readthrough	0.41 (0.12[b])
**Coagulation**				
**1**	Antithrombin-III	1.19	Antithrombin-III	0.85
**2**	Kininogen-1	1.33	Plasminogen	0.95
**3**	Prothrombin	1.59	Kininogen-1	0.74
**4**	Plasminogen	1.07	Prothrombin	0.52
**5**	Heparin cofactor 2	2.05	Alpha-2-antiplasmin	0.92
**6**	Alpha-2-antiplasmin	1.11	Plasma kallikrein	1.00
**7**	Plasma kallikrein	1.01	Coagulation factor XII	1.31
**8**	Kallistatin	1.87 (0.30[a])	Protein S	1.02 (0.99[b])
**9**	Carboxypeptidase N subunit 2	1.83 (0.34[a])	Protein Z	1.25 (0.70[b])
**10**	Coagulation factor XII	0.62	Heparin cofactor 2	0.15
**Complement**				
**1**	Complement C3	1.47	Complement factor H	1.46
**2**	Complement C4-A	1.51	Complement C3	0.63
**3**	Complement factor H	0.45	C4b-binding protein	1.25
**4**	Plasma protease C1 inhibitor	1.88	Complement C4-A	0.59
**5**	C4b-binding protein	0.70	Factor H-related protein 3	1.80 (0.02[b])
**6**	C3/C5 convertase	1.42	Factor H-related protein 1	1.35 (0.58[b])
**7**	Complement C5	1.80 (0.36[a])	C3/C5 convertase	0.67
**8**	Ficolin-3	1.12	Ficolin-3	0.90
**9**	Complement C1r	1.31 (0.75[a])	Plasma protease C1 inhibitor	0.29
**10**	Complement component C9	1.97 (0.22[a])	Complement factor I	1.05 (0.94[b])
**Immunoglobulins**				
**1**	Immunoglobulin heavy constant gamma 1	1.27	Immunoglobulin heavy constant gamma 1	0.79
**2**	Immunoglobulin heavy constant alpha 1	1.26	Immunoglobulin heavy constant alpha 1	0.80
**3**	Immunoglobulin heavy constant gamma 2	1.28	Immunoglobulin lambda constant 2	1.37
**4**	Immunoglobulin heavy constant mu	1.40	Immunoglobulin kappa constant	0.95
**5**	Immunoglobulin kappa constant	1.07	Immunoglobulin heavy constant gamma 2	0.78

**6**	Immunoglobulin lambda constant 2	0.56	Immunoglobulin heavy constant mu	0.68
**7**	Immunoglobulin kappa variable 3-20	1.74 (0.41[a])	Immunoglobulin heavy constant gamma 3	1.59 (0.28[b])
**8**	Immunoglobulin heavy variable 1-18	0.92	Immunoglobulin lambda-like polypeptide 5	1.76 (0.06[b])
**9**	Immunoglobulin kappa variable 2D-29	0.96	Immunoglobulin heavy variable 1-18	1.07
**10**	Immunoglobulin heavy variable 4-4	1.46 (0.64[a])	Immunoglobulin kappa variable 2D-29	1.04
**Lipoproteins**				
**1**	Apolipoprotein A-I	1.63	Apolipoprotein A-I	1.272
**2**	Apolipoprotein B-100	1.84	Apolipoprotein A-II	1.127
**3**	Apolipoprotein A-IV	0.76	Apolipoprotein A-IV	0.932
**4**	Apolipoprotein A-II	0.49	Apolipoprotein E	1.196
**5**	Apolipoprotein C-III	1.22	Apolipoprotein C-II	0.903
**6**	Apolipoprotein E	0.58	Apolipoprotein B-100	1.354
**7**	Apolipoprotein C-I	1.40	Apolipoprotein C-III	1.17
**8**	Apolipoprotein D	0.88	Apolipoprotein D	1.19
**9**	Apolipoprotein C-II	0.31	Apolipoprotein C-I	0.784
**10**	Apolipoprotein M	1.69 (0.45[a])	Apolipoprotein L1	0.897 (1.16[b])

Supplementary references:

1. Travers RJ, Shenoi RA, Kalathottukaren MT, Kizhakkedathu JN, Morrissey JH. Nontoxic polyphosphate inhibitors reduce thrombosis while sparing hemostasis. *Blood* 2014, **124**(22)**:** 3183-3190.
2. Shenoi RA, Kalathottukaren MT, Travers RJ, Lai BFL, Creagh AL, Lange D*, et al.* Affinity-based design of a synthetic universal reversal agent for heparin anticoagulants. *Sci Transl Med* 2014, **6**(260)**:** 260ra150.
3. Ryu JH, Messersmith PB, Lee H. Polydopamine Surface Chemistry: A Decade of Discovery. *ACS Appl Mater Interfaces* 2018, **10**(9)**:** 7523-7540.
4. Mei Y, Yu K, Lo JCY, Takeuchi LE, Hadjesfandiari N, Yazdani-Ahmadabadi H*, et al.* Polymer-Nanoparticle Interaction as a Design Principle in the Development of a Durable Ultrathin Universal Binary Antibiofilm Coating with Long-Term Activity. *ACS Nano* 2018, **12**(12)**:** 11881-11891.
5. Yu K, Andruschak P, Yeh HH, Grecov D, Kizhakkedathu JN. Influence of dynamic flow conditions on adsorbed plasma protein corona and surface-induced thrombus generation on antifouling brushes. *Biomaterials* 2018, **166:** 79-95.
6. Zhu Y, Eggert T, Araujo DJ, Vijayanand P, Ottensmeier CH, Hedrick CC. CyTOF mass cytometry reveals phenotypically distinct human blood neutrophil populations differentially correlated with melanoma stage. *J ImmunoTher Cancer* 2020, **8**(2): e000473.
7. Song X, Ji HF, Li YP, Xiong YQ, Qiu L, Zhong R*, et al.* Transient blood thinning during extracorporeal blood purification via the inactivation of coagulation factors by hydrogel microspheres. *Nat Biomed Eng* 2021, **5**(10)**:** 1143-1156.
8. Yu K, Lai BFL, Foley JH, Krisinger MJ, Conway EM, Kizhakkedathu JN. Modulation of Complement Activation and Amplification on Nanoparticle Surfaces by Glycopolymer Conformation and Chemistry. *ACS Nano* 2014, **8**(8)**:** 7687-7703.
9. Yeh CHJ, Dimachkie ZO, Golas A, Cheng A, Parhi P, Vogler EA. Contact activation of blood plasma and factor XII by ion-exchange resins. *Biomaterials* 2012, **33**(1)**:** 9-19.
10. Lee HA, Ma YF, Zhou F, Hong S, Lee H. Material-Independent Surface Chemistry beyond Polydopamine Coating. *Acc Chem Res* 2019, **52**(3)**:** 704-713.
11. Varga-Szabo D, Pleines I, Nieswandt B. Cell adhesion mechanisms in platelets. *Arterioscler Thromb Vasc Biol* 2008, **28**(3)**:** 403-412.
12. Ruggeri ZM. Platelet Adhesion under Flow. *Microcirculation* 2009, **16**(1)**:** 58-83.
13. Liu XD, Xu T, Jiang CJ, Li YP, Su BH, Zhao WF*, et al.* Ultraporous Polyquaternium-Carboxylated Chitosan Composite Hydrogel Spheres with Anticoagulant, Antibacterial, and Rapid Endotoxin Removal Profiles for Sepsis Treatment. *Biomacromolecules* 2022, **23**(9)**:** 3728-3742.
14. Weber C, Morsbach S, Landfester K. Possibilities and Limitations of Different Separation Techniques for the Analysis of the Protein Corona. *Angew Chem-Int Edit* 2019, **58**(37)**:** 12787-12794.
15. Ji HF, Li YP, Su BH, Zhao WF, Kizhakkedathu JN, Zhao CS. Advances in Enhancing Hemocompatibility of Hemodialysis Hollow-Fiber Membranes. *Adv Fiber Mater* 2023, **5:** 1198-1240.
16. Kizhakkedathu JN, Conway EM. Biomaterial and cellular implants: foreign surfaces where immunity and coagulation meet. *Blood* 2022, **139**(13)**:** 1987-1998.
17. Heestermans M, Naudin C, Mailer RK, Konrath S, Klaetschke K, Jamsa A*, et al.* Identification of the factor XII contact activation site enables sensitive coagulation diagnostics. *Nat Commun* 2021, **12**(1)**:** 5596-5612.
18. Shang XQ, Chen H, Castagnola V, Liu K, Boselli L, Petseva V*, et al.* Unusual zymogen activation patterns in the protein corona of Ca-zeolites. *Nat Catal* 2021, **4**(7)**:** 607-614.
19. Chen J, Wang AH, Liu B, Zhou Y, Luo P, Zhang ZC*, et al.* Quantitative Lysine Reactivity Profiling Reveals Conformational Inhibition Dynamics and Potency of Aurora A Kinase Inhibitors. *Anal Chem* 2019, **91**(20)**:**

13222-13229.
20. Schmaier AH. The contact activation and kallikrein/kinin systems: pathophysiologic and physiologic activities. *J Thromb Haemost* 2016, **14**(1)**:** 28-39.
21. Leslie DC, Waterhouse A, Berthet JB, Valentin TM, Watters AL, Jain A*, et al.* A bioinspired omniphobic surface coating on medical devices prevents thrombosis and biofouling. *Nat Biotechnol* 2014, **32**(11)**:** 1134-1140.
22. Smith RS, Zhang Z, Bouchard M, Li J, Lapp HS, Brotske GR*, et al.* Vascular Catheters with a Nonleaching Poly-Sulfobetaine Surface Modification Reduce Thrombus Formation and Microbial Attachment. *Sci Transl Med* 2012, **4**(153)**:** 153ra132.
23. Yau JW, Stafford AR, Liao P, Fredenburgh JC, Roberts R, Brash JL*, et al.* Corn trypsin inhibitor coating attenuates the prothrombotic properties of catheters in vitro and in vivo. *Acta Biomaterialia* 2012, **8**(11)**:** 4092-4100.
24. Zhou JJ, Penna M, Lin ZX, Han YY, Lafleur RPM, Qu YJ*, et al.* Robust and Versatile Coatings Engineered via Simultaneous Covalent and Noncovalent Interactions. *Angew Chem-Int Edit* 2021, **60**(37)**:** 20225-20230.
25. Mei Y, Yu K, Yazdani-Ahmadabadi H, Lange D, Kizhakkedathu JN. Hydrophilic Polymer-Guided Polycatecholamine Assembly and Surface Deposition. *Acs Applied Materials & Interfaces* 2022, **14**(34)**:** 39577-39590.
26. Biran R, Pond D. Heparin coatings for improving blood compatibility of medical devices. *Advanced Drug Delivery Reviews* 2017, **112:** 12-23.
27. Ai LS, Yu HY, Dai WD, Hale SL, Kloner RA, Hsiai TK. Real-Time Intravascular Shear Stress in the Rabbit Abdominal Aorta. *IEEE Trans Biomed Eng* 2009, **56**(6)**:** 1755-1764.
28. Di Stefano I, Koopmans DR, Langille BL. Modulation of arterial growth of the rabbit carotid artery associated with experimental elevation of blood flow. *J Vasc Res* 1998, **35**(1)**:** 1-7.
