# Supplementary material for: Antithrombotic coating with sheltered positive charges prevents contact activation by controlling factor XII–biointerface binding
Source: Nat Mater. 2024 Nov 12;24(4):626–36. doi: 10.1038/s41563-024-02046-0 (PMC11961369; doi:10.1038/s41563-024-02046-0)
Supplement: Supplementary file 2 — Reporting Summary [file 41563_2024_2046_MOESM2_ESM.pdf]

nature portfolio

Corresponding author(s): Weifeng Zhao, Jayachandran N Kizhakkedathu

Last updated by author(s): Oct 1, 2024

Reporting Summary

Nature Portfolio wishes to improve the reproducibility of the work that we publish. This form provides structure for consistency and transparency in reporting. For further information on Nature Portfolio policies, see our Editorial Policies and the Editorial Policy Checklist.

Statistics

For all statistical analyses, confirm that the following items are present in the figure legend, table legend, main text, or Methods section.

n/a	Confirmed	
☐	☒	The exact sample size (*n*) for each experimental group/condition, given as a discrete number and unit of measurement
☐	☒	A statement on whether measurements were taken from distinct samples or whether the same sample was measured repeatedly
☐	☒	The statistical test(s) used AND whether they are one- or two-sided<br>*Only common tests should be described solely by name; describe more complex techniques in the Methods section.*
☐	☒	A description of all covariates tested
☐	☒	A description of any assumptions or corrections, such as tests of normality and adjustment for multiple comparisons
☐	☒	A full description of the statistical parameters including central tendency (e.g. means) or other basic estimates (e.g. regression coefficient) AND variation (e.g. standard deviation) or associated estimates of uncertainty (e.g. confidence intervals)
☐	☒	For null hypothesis testing, the test statistic (e.g. *F*, *t*, *r*) with confidence intervals, effect sizes, degrees of freedom and *P* value noted<br>*Give P values as exact values whenever suitable.*
☒	☐	For Bayesian analysis, information on the choice of priors and Markov chain Monte Carlo settings
☒	☐	For hierarchical and complex designs, identification of the appropriate level for tests and full reporting of outcomes
☒	☐	Estimates of effect sizes (e.g. Cohen's *d*, Pearson's *r*), indicating how they were calculated

Our web collection on statistics for biologists contains articles on many of the points above.

Software and code

Policy information about availability of computer code

Data collection

Bruker Avance AV-300 spectrometer was used to acquire NMR spectra of designed moleculars.
Bruker 670 TensorII with an MCT/A liquid nitrogen cooled detector was used to collect FTIR spectra of designed moleculars and coatings.
Leybold LH Max 200 surface analysis system (Leybold, Cologne, Germany) equipped with a Mg Kα source at a power of 200 W was used to collect XPS spectra of designed coatings.
QCM-D (Biolin Scientific) was used for evaluation of the mass and stability of the coating on the sensor surface.
The coating thickness was obtained from ellipsometer (M-2000V, J.A. Woollam Co., Inc.).
The surface-morphologies were obtained by scanning electron microscopy (SEM, Phenom Pure, Phenom World, Netherlands).
The image of the water droplets on the surfaces were taken with a digital camera (Retiga 1300, Q-imaging Co.).
The surface zeta potentials of the coated substrates were measured using a Zetasizer Nano-ZS90 (Malvern Instruments Ltd., UK).
Blood clotting, pNA generation, and chromogenic substrate based results was detected by UV-VIS at 405 nm in kinetic mode.
A semiautomatic blood coagulation analyzer (CA-50, Sysmex Corporation, Japan) was employed to investigate the influence of SPI coating on the blood coagulation system.
The peptides were subjected to capillary source followed by the timsTOF Pro (Bruker Daltonics) mass spectrometry.
An automatic coagulation analyzer (ACL Elite Pro, werfan) was employed to investigate the activities of coagulation factors after incubating recalcified PPP with bare or SPI-coated glass vial.
3-Laser CytoFLEX Flow Cytometer (Beckman Coulter Life Sciences, Indianapolis, IN, USA) was used for flow cytometry.
The whole blood cell differential counts were measured by an automated hematology cell analyzer (BC-5100, Mindray Bio-Medical Electronics Co., Ltd., Shenzhen, China).
Blood flow simulation using computational fluid dynamics (CFD) modewas was carried out in COMSOL Multiphysics, and an extra fine mesh was generated for the computational domain.

March 2021

Data analysis	Microsoft Excel 2019, OriginPro 2018, Prism Graphpad 7.0, imageJ 1.53t, OMNIC 8, Avantage 5.967, BD FACSCanto II, Q-Sense Dfind, Northern Eclipse software were used. Mass spectra were searched against the human SwissProt database (20422 entries) concatenated with reverse decoy database using MaxQuant 1.6.15.0.

For manuscripts utilizing custom algorithms or software that are central to the research but not yet described in published literature, software must be made available to editors and reviewers. We strongly encourage code deposition in a community repository (e.g. GitHub). See the Nature Portfolio guidelines for submitting code & software for further information.

Data

Policy information about availability of data

All manuscripts must include a data availability statement. This statement should provide the following information, where applicable:

- Accession codes, unique identifiers, or web links for publicly available datasets
- A description of any restrictions on data availability
- For clinical datasets or third party data, please ensure that the statement adheres to our policy

Protein mass spectra were searched against the human SwissProt database (20422 entries) concatenated with reverse decoy database; proteome ID: UP000005640; release numbers: 2021_01/2021_01). Proteomic data, including raw data and search results have been deposited in the ProteomeXchange database with dataset identifier "PXD054476 (for Fig. 3b and 5b-c) and PXD054293 (for Fig. 6f)". All other data supporting the findings of this study are available within the article and its supplementary files.

Human research participants

Policy information about studies involving human research participants and Sex and Gender in Research.

Reporting on sex and gender	All blood samples were collected from healthy donors, and gender differences were not considered.
Population characteristics	Fresh human blood was collected from healthy, unmedicated donors aged 20-30 using vacutainer blood collection tubes, with no gender preference.
Recruitment	University of British Columbia and the West China Hospital announced the recruitment of human research participants. These volunteers were recruited by voluntary registration and disease-history screening. Volunteers with no recent use of medications affecting the coagulation system and no history of coagulation disorders were selected, as these factors could influence the experimental results regarding the material's interaction with the coagulation system.
Ethics oversight	University of British Columbia (the protocol for blood donations was approved by the University of British Columbia's clinical ethics board. UBC Ethics approval no: H10-01896, H20-00084 and H18-02553) and West China Hospital, Sichuan University (GB/T 16886.4-2003/ISO 10993-4:2002, General Administration of Quality Supervision, Inspection and Quarantine of the People's Republic of China, Standardization Administration of the People's Republic of China).

Note that full information on the approval of the study protocol must also be provided in the manuscript.

Field-specific reporting

Please select the one below that is the best fit for your research. If you are not sure, read the appropriate sections before making your selection.

☒ Life sciences ☐ Behavioural & social sciences ☐ Ecological, evolutionary & environmental sciences

For a reference copy of the document with all sections, see nature.com/documents/nr-reporting-summary-flat.pdf

Life sciences study design

All studies must disclose on these points even when the disclosure is negative.

Sample size	For animal experiments, we analyzed samples with a minimum of 4 rabbits to determine statistical similarity or differences between groups. No statistical methods were used to pre-determine the sample size. Our sample sizes are similar to those reported in previous publications:<br><br>Qiu H, Tu QF, Gao P, Li XY, Maitz MF, Xiong KQ, et al. Phenolic-amine chemistry mediated synergistic modification with polyphenols and thrombin inhibitor for combating the thrombosis and inflammation of cardiovascular stents. Biomaterials, 269 (2021) 120626<br>Leslie DC, Waterhouse A, Berthet JB, Valentin TM, Watters AL, Jain A, et al. A bioinspired omniphobic surface coating on medical devices prevents thrombosis and biofouling. Nat Biotechnol 2014, 32(11): 1134-1140
Data exclusions	No data acquired for quantitative analysis was excluded from the study.

Replication	Unless otherwise mentioned, at least 4 replications were performed for all the tests. All our attempts at replication were successful with similar results.
Randomization	All New Zealand rabbits were randomly assigned to experimental groups for all experiments.
Blinding	The establishment of animal experimental models and data collection were conducted by the same team, which was blinded to the sample groups. Samples from different groups were randomly assigned to operators for experimentation. During the subsequent blood tests, these samples were pooled and numbered in a manner that did not reveal their group characteristics. The analysis involving blood coagulation parameters, SEM, blood count assay, computational fluid dynamics (CFD) model, biological parameters were not blinded as the results comes from computational and mathematical analysis which is not effected by individual interpretation.

Reporting for specific materials, systems and methods

We require information from authors about some types of materials, experimental systems and methods used in many studies. Here, indicate whether each material, system or method listed is relevant to your study. If you are not sure if a list item applies to your research, read the appropriate section before selecting a response.

Materials & experimental systems

n/a	Involved in the study
☐	☒ Antibodies
☐	☒ Eukaryotic cell lines
☒	☐ Palaeontology and archaeology
☐	☒ Animals and other organisms
☒	☐ Clinical data
☒	☐ Dual use research of concern

Methods

n/a	Involved in the study
☒	☐ ChIP-seq
☐	☒ Flow cytometry
☒	☐ MRI-based neuroimaging

Antibodies

Antibodies used	Anti-CD42-fluorescein isothiocyanate (FITC, BD, biosciences, 555473); anti-CD62P-phycoerythrin (PE, BD, biosciences, 550561); allophycocyanin (APC) anti-human CD15 (Biolegend, 301908); FITC anti-human CD11b (Biolegend, 301330) for flow cytometry and laser scanning confocal microscope measurements. Antibodies for Human Bradykinin (BK) (Abcam, USA, ab136936), Thrombin-antithrombin (TAT) complex (Human TAT kit, Abcam, USA, ab108907), IL-6 (Thermo Fisher, 88-7066-22), FVIIa level (Human Factor VIIa Chromogenic Activity Assay Kit, ab137995), C3a and C5b-9 generation (Complement C3a Human ELISA Kit, Fisher scientific, 50-112-5293; Complement C5b-9 Human ELISA Kit, Quidel, San Diego, CA, A020(QI)) and FDP level (FDP ELISA Kit, Abbexa Ltd., ABX151504-96TESTS) were provided by the respective vendors along with their kits.
Validation	Validation of Flow cytometry of human platelets for Anti-CD42-fluorescein isothiocyanate (FITC, BD, biosciences, 555473) and anti-CD62P-phycoerythrin (PE, BD, biosciences, 550561) can be found in:https://www.bdbiosciences.com/content/dam/bdb/products/global/reagents/flow-cytometry-reagents/research-reagents/single-color-antibodies-ruo/555xxx/5554xx/555472_base/pdf/555472.pdf, and https://www.bdbiosciences.com/content/bdb/paths/generate-tds-document.cn.550561.pdf#:~:text=Anti-Human%20CD62P%20(Cat.%20No.%20550561;%20solid.<br>Validation of Flow cytometry of human neutrophils for forallophycocyanin (APC) anti-human CD15 (Biolegend, 301908); FITC anti-human CD11b (Biolegend, 301330) can be found in: https://d1spbj2x7qk4bg.cloudfront.net/en-gb/products/apc-anti-human-cd15-ssea-1-antibody-3702?displayInline=true&filename=APC%20anti-human%20CD15%20(SSEA-1)%20Antibody.pdf&leftRightMargin=15&pdf=true&topBottomMargin=15&v=20240910063028, and https://d1spbj2x7qk4bg.cloudfront.net/en-gb/products/fitc-anti-human-cd11b-antibody-8299?displayInline=true&filename=FITC%20anti-human%20CD11b%20Antibody.pdf&leftRightMargin=15&pdf=true&topBottomMargin=15&v=20240814063131.<br>Validation of Elisa test of human BK (Abcam, USA, ab136936) and TAT (Human TAT kit, Abcam, USA, ab108907) can be found in: https://www.abcam.com/en-us/products/elisa-kits/bradykinin-elisa-kit-ab136936, and https://www.abcam.com/en-us/products/elisa-kits/human-thrombin-antithrombin-complex-elisa-kit-tat-ab108907.<br>Validation of Elisa test of human IL-6 (Thermo Fisher, 88-7066-22), FVIIa level (Human Factor VIIa Chromogenic Activity Assay Kit, ab137995), C3a and C5b-9 generation (Complement C3a Human ELISA Kit, Fisher scientific, 50-112-5293; Complement C5b-9 Human ELISA Kit, Quidel, San Diego, CA, A020(QI)) and FDP level (FDP ELISA Kit, Abbexa Ltd., ABX151504-96TESTS) can be found in: https://www.thermofisher.com/elisa/product/IL-6-Human-Uncoated-ELISA-Kit-with-Plates/88-7066-22#documents-container, https://www.abcam.com/en-ca/search/page?facets.categoryType=Assay%20Kits&productSorting=&resourceSorting=relevance&keywords=factor%20viiia%20assay, https://www.fishersci.com/shop/products/human-c3a-platinum-elisa-kit-4/501125293#:~:text=eBioscience%20Human%20C3a%20Platinum%20ELISA%20Kit:, https://www.quidelortho.com/ca/en/products/microvue-assays/microvue-sc5b-9-plus-eia#1-item-c59d5a7bd7-tab, and https://www.abbexa.com/human-fdp-elisa-kit, respectively.

Eukaryotic cell lines

Policy information about cell lines and Sex and Gender in Research

Cell line source(s)	EA.hy926 cells were purchased from American Type Culture Collection (ATCC, CRL-2922) and used up to a passage number of 50. Human vascular smooth muscle cells (HVSMC, ATCC, PCS-100-012) were used to evaluate the SMC proliferation on SPI coating.

Authentication	The EA. hy926 cells were authenticated at the time of purchase from ATCC.<br><br>Electron photomicrographs of EA. hy926 cells demonstrate cytoplasmic distribution of Weibel-Palade bodies and tissue-specific organelles, characteristics of differentiated endothelial cell functions such as angiogenesis, homeostasis/thrombosis, blood pressure and inflammation.<br><br>Cell lines were not authenticated for Human vascular smooth muscle cells.
Mycoplasma contamination	The cell lines were not tested for mycoplasma contamination
Commonly misidentified lines (See ICLAC register)	No cells used in this study are commonly misidentified lines

Animals and other research organisms

Policy information about studies involving animals; ARRIVE guidelines recommended for reporting animal research, and Sex and Gender in Research

Laboratory animals	Healthy New Zealand White Rabbits (aged approximately 4 mouths and weighing about 2.5–3.5 kg, Laboratory Animal Center of West China Hospital Science Park, Sichuan University)
Wild animals	No wild animals were used in this study.
Reporting on sex	Gender differences were not considered
Field-collected samples	No field-collected samples were used in this study
Ethics oversight	This study was conducted in accordance with the National Institutes of Health Guide for the care and use of laboratory animals (NIH Publications No. 8023, revised 1978). This experiment conformed to the legal requirement in China and was approved by the ethical committee (No. K2016027) of West China Hospital, Sichuan University.

Note that full information on the approval of the study protocol must also be provided in the manuscript.

Flow Cytometry

Plots

Confirm that:

☒ The axis labels state the marker and fluorochrome used (e.g. CD4-FITC).

☒ The axis scales are clearly visible. Include numbers along axes only for bottom left plot of group (a 'group' is an analysis of identical markers).

☐ All plots are contour plots with outliers or pseudocolor plots.

☒ A numerical value for number of cells or percentage (with statistics) is provided.

Methodology

Sample preparation	Human platelets and neutrophils were used for analysis, details are given in the supplementary information. For protein corona tests, glass microspheres (Sigma) with a diameter of 3 um were used.
Instrument	BD FACSCanto II flow cytometer
Software	CytExpert 2.5
Cell population abundance	For platelet and neutrophil activation tests, a total of 10,000 events were collected for each samples for platelet and neutrophil activation tests. For protein corona tests, a total of 500,000 events were collected for each samples.
Gating strategy	The CD42-gated platelets were used for platelet activation test;the CD15-gated neutrophils were used for neutrophil activation tests; for the protein corona tests, there is no gating strategy since the significantly higher number of microspheres compared to impurities in the plasma

☒ Tick this box to confirm that a figure exemplifying the gating strategy is provided in the Supplementary Information.
